# A new generation of 1,8-diaminocarbazole building blocks for the construction of fluorescent anion receptors†

**DOI:** 10.1039/d4ra05420b

**Published:** 2024-09-19

**Authors:** Maria L. Korczak, Krystyna Maslowska-Jarzyna, Michał J. Chmielewski

**Affiliations:** a Faculty of Chemistry, Biological and Chemical Research Centre, University of Warsaw Żwirki i Wigury 101 Warsaw 02-089 Poland mchmielewski@chem.uw.edu.pl

## Abstract

We describe the synthesis of a new generation of 1,8-diaminocarbazole building blocks for the construction of anion receptors and fluorescent sensors. These new building blocks feature mildly electron-withdrawing ester substituents at positions 3 and 6 of the carbazole core, which improve anion affinities and significantly enhance solubilities, without compromising fluorescent response. To demonstrate the advantages of the new building blocks, three of them were converted into model diamide receptors R1–R3. The resulting ester-substituted receptors showed greatly improved solubilities and fluorescent response in comparison to their 3,6-dichloro-substituted predecessors, while retaining very high affinity and selectivity for oxyanions, particularly dihydrogen phosphate, even in partially aqueous solutions. In view of these promising results and the known synthetic versatility of primary amines, we envisage that the new building blocks will be useful for the construction of various classes of fluorescent anion receptors with improved solubility, affinity, and fluorescent response.

## Abstract 2

New, ester-substituted diaminocarbazole building blocks are proposed for making anion receptors with significantly improved solubility, tunability, affinity, and fluorescent response.

## Graphical abstract

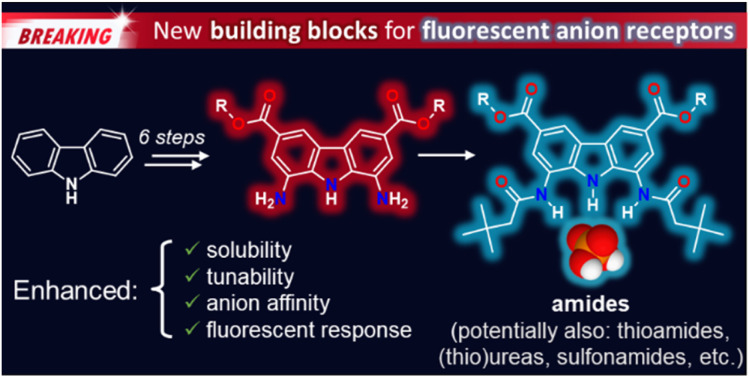

## Introduction

Synthetic molecules that strongly and selectively bind and detect anionic species hold great promise for numerous applications across scientific, industrial, and medicinal domains.^1^ Accordingly, considerable research efforts have been devoted to the development of supramolecular receptors and sensors capable of detecting various anions. Over the last 20 years, 1,8-diaminocarbazoles have emerged as particularly versatile building blocks for the construction of fluorescent and colorimetric anion sensors, owing to their powerful carbazole fluorophore and chromophore directly coupled to the anion binding site.^2,3^ Thanks to their rigid skeleton, which facilitates preorganization of the appended hydrogen bond donors, as well as to their appropriate geometry, the amide, thioamide, urea and thiourea derivatives of 1,8-diaminocarbazoles exhibit exceptionally high affinity towards Y-shaped oxyanions.^3–6^ Accordingly, 1,8-bis(amido)- and 1,8-bis(urea)carbazoles have been intensely explored for the recognition of phosphates,^7–11^ carboxylates,^3,4,12–16^ and sulfate.^5,6,17^ As a step towards practical applications, they were also used for the preparation of ion-selective electrodes,^18–20^ infrared sensors,^21^ and fluorescent sensors that selectively detect sulphate anions in mineral water.^17^ Furthermore, in recent years bis(amido)- and bis(thioamido)carbazoles have also been shown to act as potent transmembrane transporters of various biologically relevant anions.^22–26^

Despite all the advantages mentioned above, there are two main drawbacks that hamper further development of 1,8-diaminocarbazole-based anion receptors, sensors, and transporters. First, they are usually poorly soluble in most organic solvents and aqueous–organic mixtures.^3^ Second, tuning their properties by variation of substituents in the carbazole core is synthetically challenging and, in practice, restricted to positions 3 and 6. What makes it even more problematic, many electron-withdrawing substituents, introduced to improve the affinity of receptors to anions, reduce their fluorescence response.^27^

**Fig. 1:**
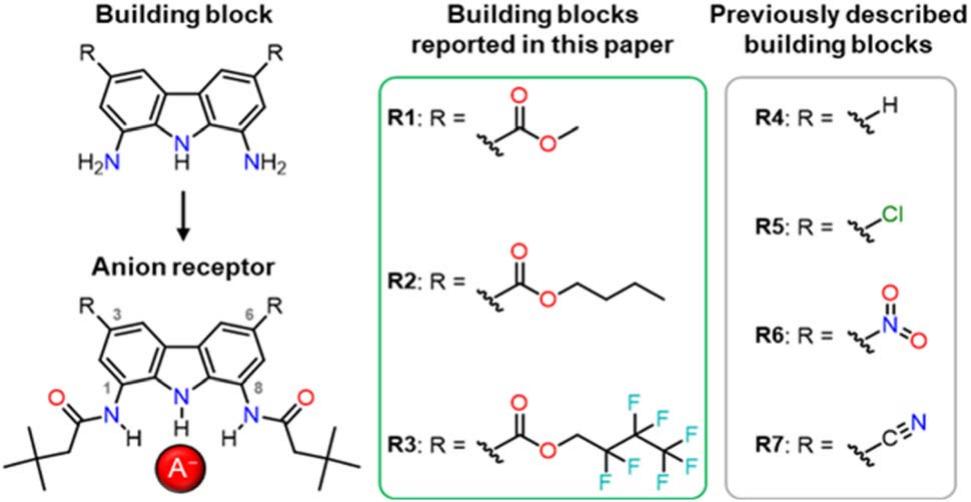
Various 1,8-diaminocarbazole building blocks for the construction of anion receptors, sensors, and transporters.

In our previous studies, we tried to solve the solubility problem by varying the substituents in the amide arms of 1,8-diamidocarbazole receptors.^3^ We noticed that the introduction of *t*-butyl substituents, well known as solubilising groups, as well as various other straight and branched alkyl chains, led to only very limited success in improving the solubility of the receptors in organic solvents. In another study, we introduced electron-withdrawing nitro- and cyano-substituents at positions 3 and 6 of the carbazole core (Fig. 1).^27^ This increased the anion binding strength of the receptors and endowed them with colorimetric anion sensing capability, but at the same time compromised their fluorescent response. Also, due to their high acidity, receptors R6 and R7 (Fig. 1) were easily deprotonated, even by moderately basic anions, such as H_2_PO_4_^−^.

The effect of substituents at positions 3 and 6 on the properties of carbazole-based anion receptors has also been studied by other research teams.^9,19^ For example, Leito and co-workers introduced *t*-Bu groups into positions 3 and 6, what improved the solubility of the receptors in organic solvents,^28^ but lowered their anion binding constants^29^ due to the electron-donating character of the alkyl substituents.

Considering the above, we envisaged that a promising approach to improving the solubility of carbazole-based receptors without compromising their anion recognition properties might be the introduction of electron-withdrawing ester groups into positions 3 and 6 of the carbazole skeleton. Ester groups have an additional advantage that their alkoxy moieties could be easily varied, what might be utilised to modulate the properties of such receptors without developing their synthesis from scratch. In this paper, we describe the synthesis of a new generation of 1,8-diaminocarbazole building blocks for the construction of anion receptors and sensors, featuring ester substituents for improved solubility, tuneability, and fluorescent response. We present also the synthesis and anion recognition studies of the first model receptors derived from these building blocks. Encouragingly, our studies show that one of these model receptors, R1, is a strong and selective turn-ON fluorescent sensor for dihydrogen phosphate, even in highly competitive, partially aqueous solutions.

## Results and discussion

### Synthesis

**Scheme 1:**
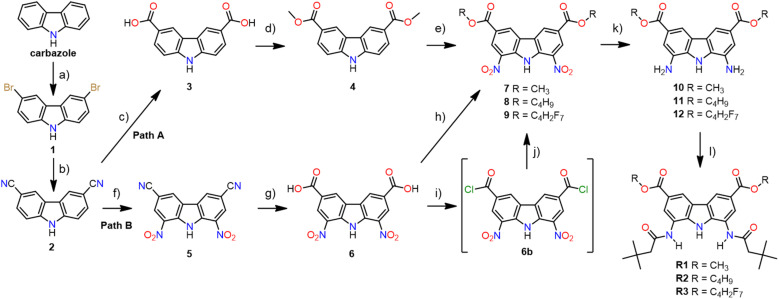
The synthesis of receptors R1–R3: (a) NBS, DMF, −5 °C, 1 h, 57%; (b) Zn(CN)_2_, dppf, Zn, Zn(OAc)_2_, Pd_2_(dba)_3_ (cat.), DMF, H_2_O, 110 °C, 72 h, 97%; (c) 2 M NaOH, CuI, 100 °C, 24 h, 98%; (d) MeOH, 95% H_2_SO_4_ (cat.) reflux, 24 h, 90%; (e) 65% HNO_3_, 95% H_2_SO_4_ (cat.), 0 °C, 0.5 h, 26%; (f) 100% HNO_3_, Ac_2_O, AcOH, 110 °C, 49%; (g) H_2_O, AcOH, 95% H_2_SO_4_ (cat.), 110 °C, 16 h, 88%; (h) MeOH, 95% H_2_SO_4_ (cat.), reflux, 24 h, 78% for 7 or BuOH, 95% H_2_SO_4_ (cat.), 135 °C, 20 h, 90% for 8; (i) SOCl_2_, DMF, 60 °C, 4 h; (j) 2,2,3,3,4,4,4-heptafluorobutyl alcohol, DCM, TEA, 85%; (k) H_2_, 0.3 bar, 10% Pd/C (cat.), AN, RT, 7 h for 10 or 5 h for 11 & 12, 77% for 10, 89% for 11, 87% for 12; (l) *t*-butylacetyl chloride, TEA, THF, RT, 1 h, 66% for R1, 56% for R2, 73% for R3.

Since efficient, chromatography-free, and multigram-scale synthesis of carbazole-3,6-dicarboxylic acid 3 from commercially available and inexpensive carbazole (Scheme 1, steps a–c) had already been developed by Weseliński *et al.*,^30^ we initially decided to use diacid 3 as a starting point of our synthetic investigations. Esterification of 3 with various alcohols, followed by nitration at positions 1 and 8 and reduction of the nitro groups, was expected to yield the desired building blocks 10–12 (Scheme 1, path A). Unfortunately, however, while esterification of 3 with MeOH was easy and high yielding (90%), the subsequent nitration of the dimethyl ester 4 proved to be very problematic due to the non-repeatability of the procedure and the low yields of the desired product (3–26%). Since this step would have to be optimised separately for each ester, we decided to test path B instead (Scheme 1, path B), because in path B the problematic nitration of positions 1 and 8 occurs earlier (at step 3 rather than at step 5), and hence there is no need to optimise its conditions for each targeted receptor. In fact, the nitration of 3,6-dicyanocarbazole 2 had previously been described by us,^27^ and now we have successfully scaled this procedure up, from 3 to 12 mmol of the substrate.

In path B, the key step is the hydrolysis of 3,6-dicyano-1,8-dinitrocarbazole 5 to diacid 6, because this is where the synthetic path branches out into individual esters. Unfortunately, alkaline hydrolysis of dinitrile 5, under the same conditions that led to nearly quantitative hydrolysis of dinitrile 2, resulted in extensive decomposition and hence low yields of 6. Therefore, as an alternative, acidic conditions were developed for this transformation. The best results were obtained after heating 5 to reflux (approx. 116 °C) in a mixture of sulfuric acid, acetic acid, and water (2 : 1 : 1 v/v), for 16 h. Using this method, the desired 1,8-dinitrocarbazole-3,6-dicarboxylic acid 6 was obtained in 88% yield, after repeated crystallisation from hot DMSO. The esterification of 6 with appropriate alcohols, used as both reagents and solvents, led to diesters 7 and 8 in 78% and 90% yield, respectively. Diester 9 was obtained from the same substrate, but using a different method, due to the higher cost of 2,2,3,3,4,4,4-heptafluorobutyl alcohol. Thus, diacid 6 was first converted to acid chloride 6b (in a reaction with thionyl chloride), and then reacted with 2,2,3,3,4,4,4-heptafluorobutyl alcohol to give the desired diester 9 in 85% yield. To selectively reduce the two nitro groups in 7–9 without affecting the ester substituents, we used 0.3 bar of H_2_ in the presence of 10% Pd/C as a catalyst, in acetonitrile as a solvent. The same method was used by us before for the reduction of 3,6-dichloro-1,8-dinitrocarbazole.^2^ Thus, the desired building blocks 10, 11, and 12 were obtained in 77%, 89%, and 87% yield, respectively. Finally, acylation of 10–12 with *t*-butylacetyl chloride gave the desired diamide receptors R1–R3 in acceptable yields (66% for R1, 56% for R2, 73% for R3). Chromatographic purification of the new receptors was significantly easier in comparison to that of other diamidocarbazoles, due to their improved solubility and hence expanded range of available eluents.

Solubility assessment of the final receptors R1–R3 was conducted in various solvents, including DCM, ACN, EA, DMF, and DMSO, and compared to the solubility of the previously described diamides R4–R7. Overall, esters R1–R3 exhibited significantly higher solubility than the carbazole-based receptors R4–R7. While receptors R4–R7 showed appreciable solubility only in DMF and DMSO, the ester-substituted receptors R1–R3 demonstrated good solubility in a broader range of solvents. For example, semi-quantitative solubility studies in ethyl acetate revealed that R3 dissolves at approximately 33.4 mg per 100 g of solvent, compared to only 4.4 mg for the 3,6-dichloro-substituted receptor R5 (see ESI, Section 5.1†). Even in DMSO, the difference was immediately apparent: the ester-substituted receptors dissolved much more readily, upon simple mixing, while other derivatives required ultrasounds and heating to achieve full dissolution.

### Anion binding studies

**Fig. 2:**
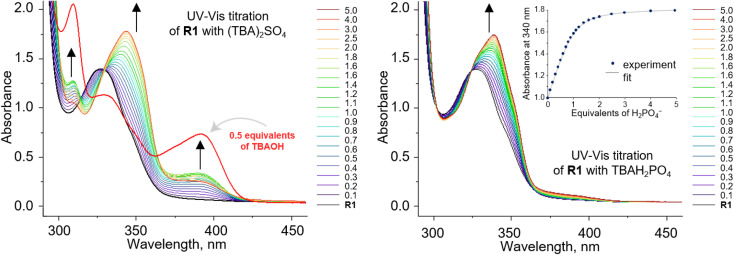
UV-vis titration of 0.1 mM R1 with (TBA)_2_SO_4_ (left) and TBAH_2_PO_4_ (right) in DMSO/0.5% H_2_O. Deprotonation of R1 occurs in the presence of sulfates.

To investigate the anion recognition properties of the new receptors, we selected five anions with diverse shapes, solvation energies, and basicities as model guests: Cl^−^, PhCOO^−^, H_2_PO_4_^−^, CH_3_COO^−^, and SO_4_^2−^. The presence of integrated chromophores and fluorophores in the carbazole receptors allows their binding properties to be conveniently studied using various techniques, including NMR, UV-vis, and fluorescence titrations. Given that the stability constants of halide complexes with 1,8-diamidocarbazoles are typically low, we employed ^1^H NMR titration to measure the binding affinity of R1 to chloride. On the contrary, the stability constants of 1,8-diamidocarbazole complexes with oxyanions typically exceed the range that could be reliably determined by ^1^H NMR titrations. Therefore, UV-vis titrations were used to quantify the binding in these cases (Fig. 2).

The stability constants of the complexes of R1–R3 with various anions are presented in Table 1 and compared with the previously published data for receptors R4–R7. For the model receptor R1, the binding constants follow the order 
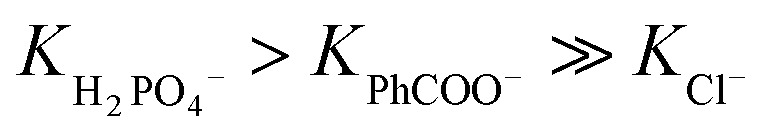
, as was the case for the previously reported diamidocarbazoles. The selectivity of R1 for H_2_PO_4_^−^ over Cl^−^, expressed as the ratio of the respective binding constants, exceeds 700, while the selectivity for PhCOO^−^ over Cl^−^ reaches 170. The affinities of R1 to dihydrogen phosphate, benzoate, and chloride are similar to those of R5, suggesting similar electron-withdrawing strength of ester and chlorine substituents. Differences are apparent, however, in the case of the more basic acetate and sulfate anions. CH_3_COO^−^ and SO_4_^2–^ appear to cause slight deprotonation of R1, as inferred from the comparison of the UV-vis and fluorescence titrations of R1 with TBA salts of sulfate and acetate and with TBAOH (new peaks appear in the UV-vis spectra, with absorbance maxima at 309 and 392 nm, Fig. 2, left, and ESI 3.11†). This behaviour contrasts with R5, which does not undergo appreciable deprotonation under similar conditions.^3,31^ It is worth noting, however, that H_2_PO_4_^−^ does not deprotonate R1 (Fig. 2, right), even though it does so with the highly acidic receptors R6 and R7 (Table 1).

**Table 1:** Logarithms of binding constants (*K*_a_) for receptors R1–R7 with various anions in DMSO/0.5% H_2_O^a^^,^^b^

Anion	Logarithms of binding constants, log *K*_a_						
	R1^c^, –COOCH_3_	R2^c^, –COOC_4_H_9_	R3^c^, –COOC_4_H_2_F_7_	R4^d^, –H	R5^d^, –Cl	R6^d^, –NO_2_	R7^d^, –CN
Cl^−^e	2.09	n.d.	n.d.	1.68	2.20	2.49	2.54
H_2_PO_4_^−^	4.94	4.72	5.19^f^	4.01	4.91	5.40^f^	Deprotonation
PhCOO^−^	4.33	4.20	4.01^f^	3.67	4.47	Deprotonation	Deprotonation
CH_3_COO^−^	Deprotonation	n.d.	n.d.	4.07	4.95	n.d.	n.d.
SO_4_^2–^	Deprotonation	n.d.	n.d.	n.d.	n.d.	n.d.	n.d.

a n.d. = not determined.

b Tetrabutylammonium salts were used as anion sources, 298 K.

c Fitting errors are provided in the ESI.

d Value from ref. 27.

e DMSO-*d*_6_/0.5% H_2_O was used instead of DMSO/0.5% H_2_O.

f Estimated complexation constant, disregarding partial deprotonation.

The alkoxy groups on the rear side of the carbazole cleft seem to have minor influence on anion affinities. For instance, replacing methoxy groups in R1 with butoxy groups in R2 slightly decreased the receptor's affinity for both anions (from log *K*_a_ = 4.94 to log *K*_a_ = 4.72 for H_2_PO_4_ and from log *K*_a_ = 4.33 to log *K*_a_ = 4.20 for benzoate, Table 1). Conversely, substituting the methoxy groups with –OCH_2_(CF_2_)_2_CF_3_ in R3 appears to increase the affinity for H_2_PO_4_ (from log *K*_a_ = 4.94 to log *K*_a_ = 5.19) and decrease the affinity for benzoate (from log *K*_a_ = 4.33 to log *K*_a_ = 4.01). However, R3 seems to undergo slight deprotonation in the presence of both anions, so the respective apparent binding constants should be interpreted with caution.

The very high oxyanion affinity in DMSO/0.5% H_2_O prompted us to increase the amount of water in the solvent mixture. In the presence of 10% H_2_O in DMSO, dihydrogen phosphate and benzoate still bind to R1 appreciably, giving 
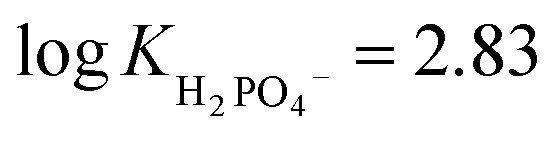
 and log *K*_PhCOO^−^_ = 2.81, while acetate and sulfate partially deprotonate R1 (see ESI 3.7–3.10†).

### Fluorescent anion sensing

**Fig. 3:**
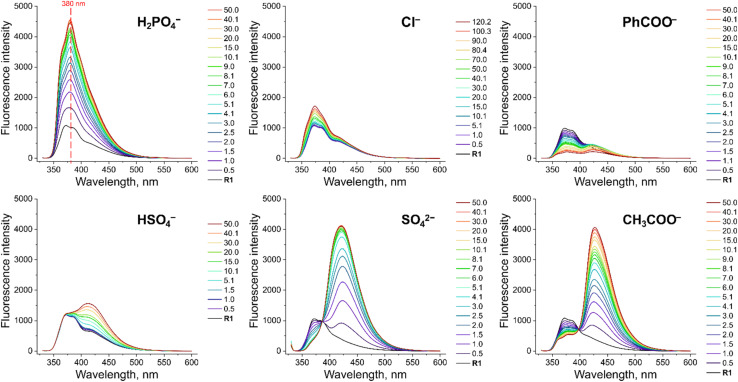
Fluorescence spectra of 10 μM R1 in the presence of various TBA salts in DMSO/0.5% H_2_O at 298 K. EX WL: 324 nm.

**Fig. 4:**
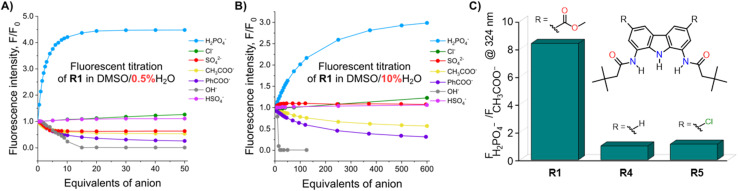
The fluorescence intensity of 10 μM solution of R1 at 380 nm in the presence of various anions in (A) DMSO/0.5% H_2_O and (B) DMSO/10% H_2_O at 298 K. EX WL: 324 nm. (C) The fluorescence intensity ratios of phosphate and acetate complexes of receptors R1, R4 and R5 in the presence of 50 equiv. of the anions in DMSO/0.5% H_2_O. EX WL: 324 nm, EM WL: 380 nm.

To investigate the fluorescent sensing capabilities of the new receptors, we performed fluorescent titrations of R1 with the same set of model anions, starting again in DMSO/0.5% H_2_O (Fig. 3). For chloride, a relatively small (up to 50%) and quasi-linear increase in fluorescence emission was observed, in accord with the rather low affinity of R1 to this anion (emission maximum at 371 nm). Notably, the fluorescence increase did not reach plateau even after the addition of 100 equivalents of chloride. In contrast, benzoate, which binds much more strongly to R1, led to a significant decrease in fluorescence intensity at 380 nm, reducing it to 20% of its initial value. Strikingly, dihydrogen phosphate induced a huge fluorescent turn-ON response, resulting in 450% increase in fluorescence at 380 nm (Fig. 3 and 4A). Other oxyanions, such as hydrogensulfate, sulfate, and acetate, gave a very different fluorescent response, with minor changes at 380 nm and the emergence of a new peak at 427 nm, likely due to deprotonation of R1 (Fig. 3; see also Fig. S58† for fluorescence titration with TBAOH).

Somewhat surprisingly, similar behaviour was observed in DMSO/10% H_2_O – a significantly more competitive medium, in which the investigated oxyanions are more strongly hydrated, and therefore less basic (Fig. 4B). Such selectivity for H_2_PO_4_^−^ over CH_3_COO^−^ was not observed for the previously published carbazole-based receptors R4 and R5 (Fig. 4C). The high selectivity likely results from the finely fitted acidity of R1, which is not deprotonated by H_2_PO_4_^−^, but deprotonates in the presence of the competing sulfate and acetate anions. Thus, our results demonstrate that diamidocarbazole receptors can be tailored to target specific anions through strategic modification of their substituents at positions 3 and 6, yielding highly selective fluorescent anion sensors.

### Determination of H_2_PO_4_^−^ in water using receptor R1

Dihydrogen phosphate (H_2_PO_4_^−^) plays a critical role in various biochemical reactions^32^ and physiological processes,^33^ including signal transduction,^34^ gene regulation,^35^ and energy storage.^36^ Abnormal levels of phosphate in biological fluids are linked to several diseases, such as vascular calcification, hypertension, vitamin D deficiency, hyperparathyroidism, and tumour growth.^37–39^ Sensitive and selective fluorescent detection of phosphate could thus be very useful in biology and medicine, with potential applications in studying phosphate metabolism *in vivo*, cell imagining^32,40^ and medical diagnostics. Additionally, selective phosphate sensors could be useful also in agriculture and environmental protection – for instance, in monitoring phosphorus fertilizers in soil and in surface waters, where excessive phosphorous may cause eutrophication and algal blooms.^41^ However, despite its significance, selective sensors for dihydrogen phosphate are still rare.^42^

**Fig. 5:**
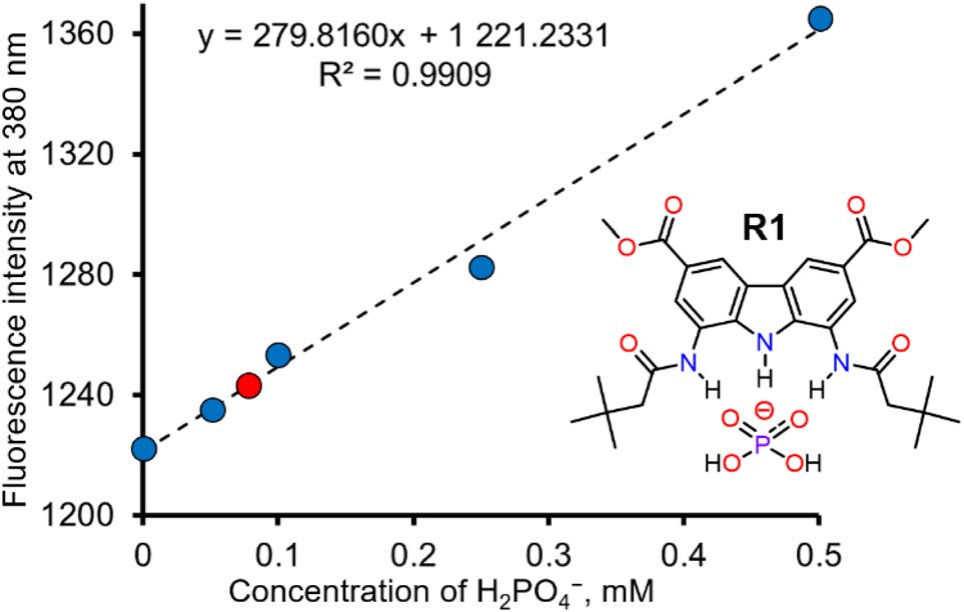
Determination of H_2_PO_4_^−^ in real water sample using selective sensor R1.

In view of the exceptional affinity and selective fluorescent response of R1 toward H_2_PO_4_^−^, we decided to explore its ability to quantify H_2_PO_4_^−^ in aqueous samples. Since simple, hydrogen bonding and charge neutral receptors like R1 do not effectively bind anions in pure water, all the investigated aqueous solutions were diluted tenfold with DMSO. Unlike in the case of fluorescence titrations described above, this time we used sodium salt of dihydrogen phosphate, because it is more relevant to real-life applications. The addition of aqueous NaH_2_PO_4_ led to a vivid increase in the fluorescence intensity of R1. The first 4 solutions were used to construct a calibration curve, shown in Fig. 5. A linear correlation was observed between the fluorescence of R1 and the concentration of dihydrogen phosphate in the concentration range of 0 to 0.5 mM H_2_PO_4_^−^ (Fig. 5). Using this calibration curve (correlation coefficient *R* = 0.9954), we successfully determined the concentration of dihydrogen phosphate in the fifth solution (0.075 mM), with a relative error of 3.7% (see ESI, Section 5†). The limit of detection (LOD) for this method was estimated to be 0.02 mM (see ESI, Section 5†).

## Conclusions

In summary, we have developed the synthesis of a new generation of diaminocarbazole building blocks for the construction of fluorescent anion receptors. The new building blocks offer improved solubility, affinity, and fluorescent response owing to ester substituents at positions 3 and 6 of the carbazole core. The advantages of the new building blocks were demonstrated through the synthesis of three model diamidocarbazole receptors. One of these receptors was shown to have a strong turn-ON fluorescent response for dihydrogen phosphate, even in partially aqueous solutions.

In the future, the improved solubility of ester-substituted carbazoles is expected to enable the construction of receptors with significantly more sophisticated molecular structure, such as macrocycles, cryptands, catenanes, and rotaxanes, while their selective fluorescent response promises utility in sensing applications. Furthermore, the possibility of varying alkoxy moieties offers tunability for specific applications, such as anion sensing in water, anion transport across lipid bilayers, or selective extraction of anions from aqueous phase. Altogether, our findings showcase diaminocarbazoles as privileged building blocks for the construction of diverse families of fluorescent anion receptors for various applications.

## Author contributions

M. L. K.: investigation (synthesis, ^1^H NMR titrations, UV-vis titrations, fluorescence titrations); data analysis; manuscript writing. K. M.-J.: conceptualisation; investigation (UV-vis titrations, fluorescence titrations); data analysis; visualisation; manuscript writing and editing. M. J. C.: conceptualisation; funding acquisition; supervision; manuscript writing and editing. All authors have read and agreed to the published version of the manuscript.

## Data availability

The data supporting this article have been included as part of the ESI.†

## Conflicts of interest

There are no conflicts to declare.

## Supplementary Material

RA-014-D4RA05420B-s001
