# Supplementary material for: A new generation of 1,8-diaminocarbazole building blocks for the construction of fluorescent anion receptors
Source: RSC Adv. 2024 Sep 19;14(41):29883–9. doi: 10.1039/d4ra05420b (PMC11411501; doi:10.1039/d4ra05420b)
Supplement: RA-014-D4RA05420B-s001 [file RA-014-D4RA05420B-s001.pdf]

Supplementary Information (SI) for RSC Advances.
This journal is © The Royal Society of Chemistry 2024

Electronic Supplementary Information (ESI)

A new generation of 1,8-diaminocarbazole building blocks for the construction of fluorescent anion receptors

Maria L. Korczak[a], Krystyna Maslowska-Jarzyna[a] and Michał J. Chmielewski*[a]

[a] *Faculty of Chemistry, Biological and Chemical Research Centre, University of Warsaw*
Żwirki i Wigury 101, 02-089 Warszawa, Poland
E-mail: mchmielewski@chem.uw.edu.pl

Contents

1 **General information** ... 3

1.1 Materials ... 3

1.2 Instruments and methods ... 4

2 **Synthetic procedures** ... 5

2.1 Compounds **1**-**3** ... 5

2.2 Diester **4** ... 6

2.3 3,6-Dicyano-1,8-dinitrocarbazole **5** ... 7

2.4 1,8-Dinitrocarbazole-3,6-dicarboxylic acid **6** ... 8

2.5 Compound **7** ... 10

2.6 Compound **8** ... 12

2.7 Compound **9** ... 14

2.8 Compound **10** ... 17

2.9 Compound **11** ... 19

2.10 Compound **12** ... 21

2.11 Receptor **R1** ... 24

2.12 Receptor **R2** ... 26

2.13 Receptor **R3** ... 28

3 **Binding studies of receptor R1** ... 31

3.1 General procedure for ^{1}H NMR titrations ... 31

3.2 General procedure for UV-vis titrations ... 31

3.3 Data fitting ... 32

3.4 ^{1}H NMR titration of **R1** with TBACl. ... 33

3.5 UV-vis titration of **R1** with TBACl. ... 36

3.7 UV-vis titration of **R1** with TBAPhCOO. ... 37

3.8 UV-vis titration of **R1** with $TBACH_3COO$. ... 41

3.9 UV-vis titration of **R1** with $TBAH_2PO_4$. ... 46

3.10 UV-vis titration of **R1** with $(TBA)_2SO_4$. ... 51

3.11 UV-vis titration of **R1** with TBAOH. ... 55

3.12 UV-vis titration of **R1** with $TBAHSO_4$. ... 56

3.13 UV-vis titration of **R2** with $TBAH_2PO_4$. ... 57

3.14 UV-vis titration of **R2** with TBAPhCOO. ... 60

3.15 UV-vis titration of **R3** with $TBAH_2PO_4$. ... 63

3.16 UV-vis titration of **R3** with TBAPhCOO. ... 66

4 **Fluorescence studies of R1** ... 69

5 **Determination of $H_2PO_4^-$ in water using R1** ... 71

5.1 Determination of solubility of receptors **R1**, **R2**, **R3** and **R5**. ... 73

1 General information

1.1 Materials

All solvents and reagents were commercially available and used as received unless otherwise stated.

Alfa Aesar: tetra-n-butylammonium sulfate ($(TBA)_2SO_4$; 50% w/w aq. soln., 41723).

Acros Organics: hydrogen chloride (HCl; pure, 1N solution in diethyl ether, 368461000).

Chempur: methanol (MeOH, 116219904, > 99.8%).

Deutero: DMSO-d_6 (C_2D_6SO, 99.8% D).

Euriso-top: DMSO-d_6 + 0.03%TMS v/v (> 99.8% D), chloroform-*d* ($CDCl_3$, 99.80% D, D007HAG), acetone-d_6 (C_3D_6O, 99.80% D).

Honeywell: methanol (MeOH; ≥ 99.8% pure p.a., 32213).

Linegal Chemicals: dichloromethane (DCM, pure p.a., 50-8124.4, freshly distilled), ethyl acetate (50-451000, dried and freshly distilled), hexane - fraction from kerosene (50-1009, freshly distilled).

POCH S.A.: acetic acid (AcOH; 99.5-99.9%, pure p.a.-basic, BA8760114), acetic anhydride (Ac_2O; ACS reagent, pure p.a., 693870115), nitric acid (HNO_3; 65%, pure p.a., BA9603115), hydrochloric acid (HCl; 35-38%, pure p.a., BA5283115), sulfuric acid (H_2SO_4, min. 95%, pure p.a., BA5000115), sodium hydroxide (NaOH; 98.8%, pure p.a.-basic, BA0981118), potassium hydroxide (KOH, 85%, pure p.a. basic, BA6800113), triethylamine anhydrous (TEA; pure p.a., 848930117), diethyl ether ($C_4H_{10}O$; 99.5%, pure p.a.-basic, stab. with BHT, BA4210114), tetrahydrofuran (THF, pure p.a.-basic, BA8200118).

Sigma-Aldrich: acetone (puriss. p.a., ≥ 99.5% by GC, 32201), acetonitrile (ACN; ≥ 99.9%, 34998), 3,3-dimethylbutyryl chloride (*tert*-butylacetyl chloride; 99%, B88802), methanol (MeOH; HPLC grade, ≥ 99.9%, 34860), nitric acid (fuming HNO_3; extra pure, 100%, 1004551000), dimethyl sulfoxide (DMSO; anhydrous, ≥99.9%, 276855), tetrabutylammonium chloride (TBACl; ≥ 99.0%, 86852), 2,2,3,3,4,4,4-heptafluoro-1-butanol ($C_4H_3F_7O$; 98%, H1604), diethylene glycol methyl ether ($C_5H_{12}O_3$; ≥ 99.0%, 579548), n-butanol (n-BuOH; 99.9%, 537993), activated charcoal (100 mesh particle size powder; 242276), palladium on activated charcoal (Pd/C; 10% Pd basis, 75990), thionyl chloride ($SOCl_2$; ≥ 99%, 230464), copper(I) iodide (CuI; purum, ≥99.5%, 03140), tetrabutylammonium benzoate (TBAPhCOO; ≥ 99.0%, 86837), tetrabutylammonium phosphate monobasic ($TBAH_2PO_4$; ≥ 99.0% (T), 86833), sodium hydroxide solution (2M NaOH, Titripur, 1091361000), tetrabutylammonium hydroxide 30-hydrate (TBAOH; ≥ 99.0%, 86859), tetrabutylammonium acetate ($TBACH_3COO$; ≥ 99.0%, 86835).

TCI: 2,2,3,3,4,4,4-heptafluoro-1-butanol ($C_4H_3F_7O$; > 98.0%, H0548).

VWR: dimethylformamide (DMF, for HPLC, 83635320, > 99.90%).

1.2 Instruments and methods

Nuclear magnetic resonance (NMR) spectroscopy

NMR spectra were recorded using Agilent NMR (^{1}H: 400 MHz, ^{13}C: 100 MHz) or Bruker AM-500 (^{1}H: 500 MHz, ^{13}C: 126 MHz) spectrometers at ambient temperature in $(CD_3)_2SO$, $CDCl_3$ or $(CD_3)_2CO$. Chemical shifts, δ, are reported in parts per million (ppm) and coupling constants, J, are given in ertz (Hz). The NMR spectra were referenced to the solvent residual signal (^{1}H: δ_{DMSO} = 2.500 ppm, $\delta_{chloroform}$ = 7.260 ppm, $\delta_{acetone}$ = 2.050 ppm, ^{13}C: δ_{DMSO} = 39.50 ppm, $\delta_{chloroform}$ = 77.16 ppm, $\delta_{acetone}$ = 29.84 ppm). Data are reported as follows: chemical shift, multiplicity (s – singlet, bs – broad singlet, d – doublet, t – triplet, dd – doublet of doublets, dt – doublet of triplets, etc.), coupling constant and integration.

Mass spectrometry

The ESI-MS spectra were obtained using API 3000 (Applied Biosystems) and AutoSpec Premier (Waters) mass spectrometers with methanol as the spray solvent.

Elemental analysis

Elemental analysis was performed using an UNIcube elementar analyser from Elementar.

TLC

TLC was carried out on Merck silica gel 60 F_{254} plates.

Preparative chromatography

Preparative chromatography was done manually on Merck silica gel 60 (230-400 mesh) or with the aid of Teledyne ISCO CombiFlash instrument using RediSep normal-phase silica flash columns.

2 Synthetic procedures

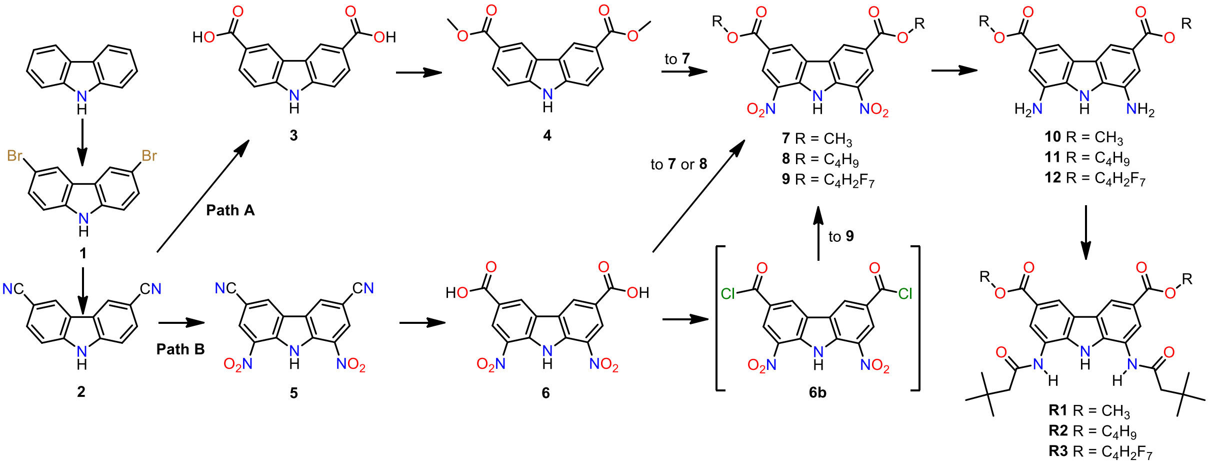

Scheme S1. Synthetic pathways to the new 1,8-diaminocarbazole-based building blocks **10-12** and model receptors **R1**-**R3**.

2.1 Compounds 1-3

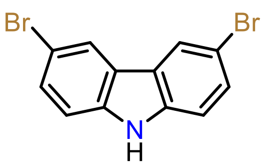

1

$C_{12}H_7Br_2N$ = 325.00

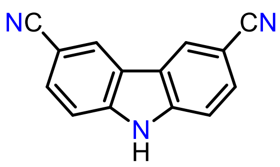

2

$C_{14}H_7N_3$ = 217.23

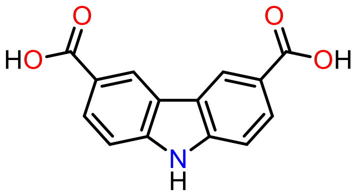

3

$C_{14}H_9NO_4$ = 255.05

Compounds **1**-**3** were obtained according to literature procedures.[1]

[1] Ł. J. Weseliński, R. Luebke, M. Eddaoudi, A convenient preparation of 9H-carbazole-3,6-dicarbonitrile and 9H-carbazole-3,6-dicarboxylic acid, *Synthesis* **2014**, *46*, 596-599.

2.2 Diester 4

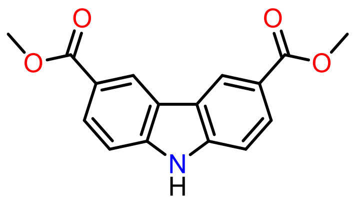

4

$C_{16}H_{13}NO_4$ = 283.28

A 500 ml round-bottom flask was equipped with a magnetic stirring bar and a reflux condenser and charged with diacid **3** (4.15 g, 16.3 mmol), methanol (200 ml), and sulfuric(VI) acid (95%, 7.61 g, 73.7 mmol). The reaction mixture was heated under reflux for 26 h. After this time the mixture was cooled down to room temperature and put in a fridge for 24 h. The precipitate was filtered off and rinsed with cold MeOH (3 × 20 ml). The product was dried in a desiccator over KOH under high vacuum to yield 4.14 g (90%) of **4** as a white solid. ^{1}H NMR in accord with published data.[2]

^{1}H NMR (400 MHz, DMSO-d_6) δ_{DMSO}: 12.10 (s; 1H; NH); 8.91 (d; J = 1.7 Hz; 2H; carbazole CH-4/5); 8.07 (dd; J_1 = 8.86 Hz; J_2 =1.7 Hz; 2H; carbazole CH-2/7); 7.62 (dd; J_1 =8.6 Hz; J_2 = 0.6 Hz; 2H; carbazole CH-1/8); 3.89 (s; 6H, CH_3).

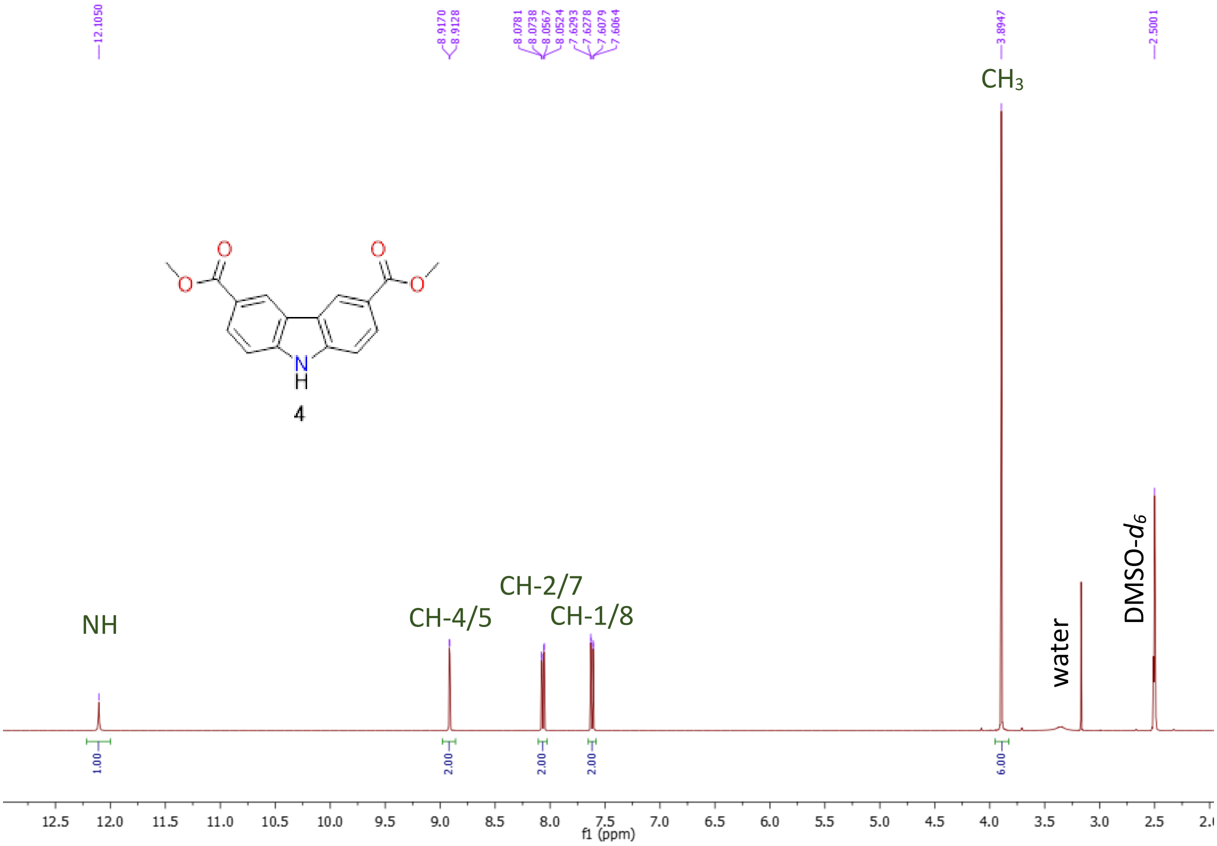

Figure S1. ^{1}H NMR spectrum of diester **4** in DMSO-d_6.

[2] C. A. Rowland, G. P. A. Yap, E. D. Bloch, Novel syntheses of carbazole-3,6-dicarboxylate ligands and their utilization for porous coordination cages, *Dalton Trans.* **2020**, *49*, 16340-16347.

2.3 3,6-Dicyano-1,8-dinitrocarbazole 5

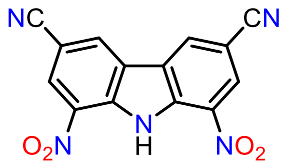

5

$C_{14}H_5N_5O_4$ = 307.03

Compound **5** was obtained as described by us previously,[3] except that the reaction was scaled up from 3 mmol to 12 mmol of the substrate. The product was purified by column chromatography on approx. 100 g of silica, using 0.1% HCl in DCM/hexane (9/1) as an eluent. Fractions containing pure product were combined and evaporated on a rotary evaporator. The solid residue was dried in a desiccator over KOH under high vacuum. Yield: 1.79 g (49%) of **5** as yellow solid.

^{1}H NMR (400 MHz, DMSO-*d*$_6$) δ_{DMSO}: 11.83 (s, 1H; NH), 9.34 (d, *J* = 1.4 Hz, 2H; CH-4/5 or CH-2/7), 9.00 (d, *J* = 1.4 Hz, 2H; CH-4/5 or CH-2/7).

HR MS (TOF MS ES$^-$) m/z calcd. for $C_{14}H_4N_5O_4^-$: 306.0263 found: 306.0262.

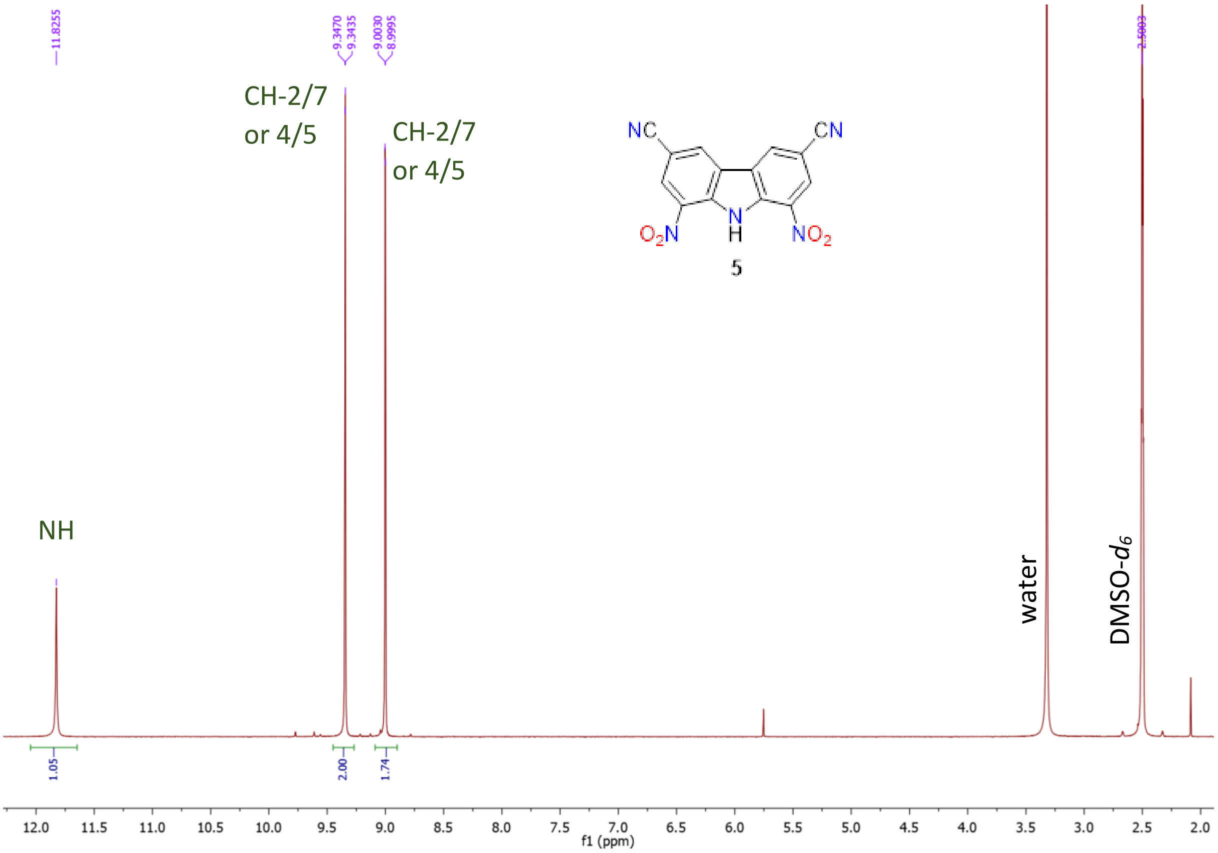

Figure S2. ^{1}H NMR spectrum of 3,6-dicyano-1,8-dinitrocarbazole **5** in DMSO-*d*$_6$.

---

3 K. Maslowska-Jarzyna, M. L. Korczak, J. A. Wagner, M. J. Chmielewski, Carbazole-based colorimetric anion sensors, *Molecules* **2021**, *26*, 3205–3221.

2.4 1,8-Dinitrocarbazole-3,6-dicarboxylic acid **6**

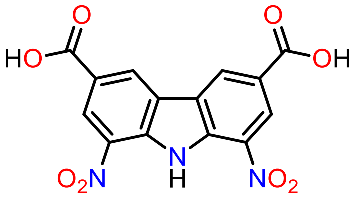

6

$C_{14}H_7N_3O_8$ = 345.22

<u>Alkaline hydrolysis</u>

A 100 ml single-neck round-bottom flask was charged with 3,6-dicyano-1,8-dinitrocarbazole **5** (652 mg, 3.00 mmol), CuI (6.3 mg, 0.03 mmol), NaOH (1.71 g, 42.7 mmol, as a solution in 40 ml of deionized water), and equipped with a stirring bar and a reflux condenser. The reaction mixture was intensively stirred and refluxed for 28 h. After this time the mixture was cooled down to room temperature and deionized water was added until the mixture become homogenous. Then, active carbon (200 mg) was added and the mixture was refluxed for 1 h. After this time, the mixture was filtered through Celite, which was washed with 2M aqueous solution of NaOH. The combined filtrates were acidified with 2M HCl (46.5 ml) to precipitate the product. The suspension was stored in a fridge for 1h, then centrifuged (15 min, 3000 rpm) and the separated solid washed with deionized water (80 ml) acidified with 2M HCl (3 ml). The crude product was redissolved in deionized water and 2M NaOH (55 ml), precipitated again with 2M HCl (70 ml), centrifuged (15 min, 3000 rpm), washed with water, and collected by filtration on a Schott filter funnel G4. The crude product was washed with DMSO (3×5 ml), deionized water (3×5 ml) and methanol (3×5 ml). The thus-obtained solid was dried in an oven at 65 °C for 24 h. Yield: 376 mg (36 %) of a light beige solid.

<u>Acidic hydrolysis</u>

A 250 ml two-neck round-bottom flask was charged with 3,6-dicyano-1,8-dinitrocarbazole **5** (1.657 g, 5.397 mmol), deionized water (30 ml), acetic acid (30 ml), and sulfuric(VI) acid (95%, 60 ml). Then the flask was equipped with a stir bar, reflux condenser and thermometer. The reaction mixture was heated to 110°C for 16 h with intense stirring. After this time the mixture was cooled down, centrifuged (5 min, 3500 rpm) and the thus-collected solid was washed with deionized water (3×30 ml) and re-dissolved in hot DMSO (100 ml). The solution was slowly cooled down to room temperature and deionized water (100 ml) was added. The precipitated product was collected by centrifugation (5 min., 3500 rpm) and washed with deionized water (3×30 ml). ^{1}H NMR spectrum revealed that the hydrolysis was not complete, so the whole procedure was repeated. This time the conversion was full and after washing with deionized water and drying in an oven at 85°C, pure product was obtained as a white solid. Yield: 1.633 g (88%).

^{1}H NMR (500 MHz, DMSO-*d*$_6$) δ_{DMSO}: 13.59 (s, 2H, COOH), 11.63 (s, 1H, NH), 9.55 (d, *J* = 1.4, 2H, CH4/5 or CH2/7), 8.87 (d, *J* = 1.4 Hz, 2H, CH4/5 or CH2/7);

^{13}C NMR (126 MHz, DMSO-*d*$_6$) δ_{DMSO}: 165.66, 135.36, 132.42, 130.25, 125.67, 124.56, 124.25.

HR MS (TOF MS ES$^-$): m/z calcd. For $C_{14}H_6N_3O_8^-$: 344.0155, found: 344.0160;

Elemental Analysis calcd. for $C_{14}H_7N_3O_8$: C, 48.71; H, 2.04; N, 12.17; found: C, 48.60; H, 2.05; N, 12.07.

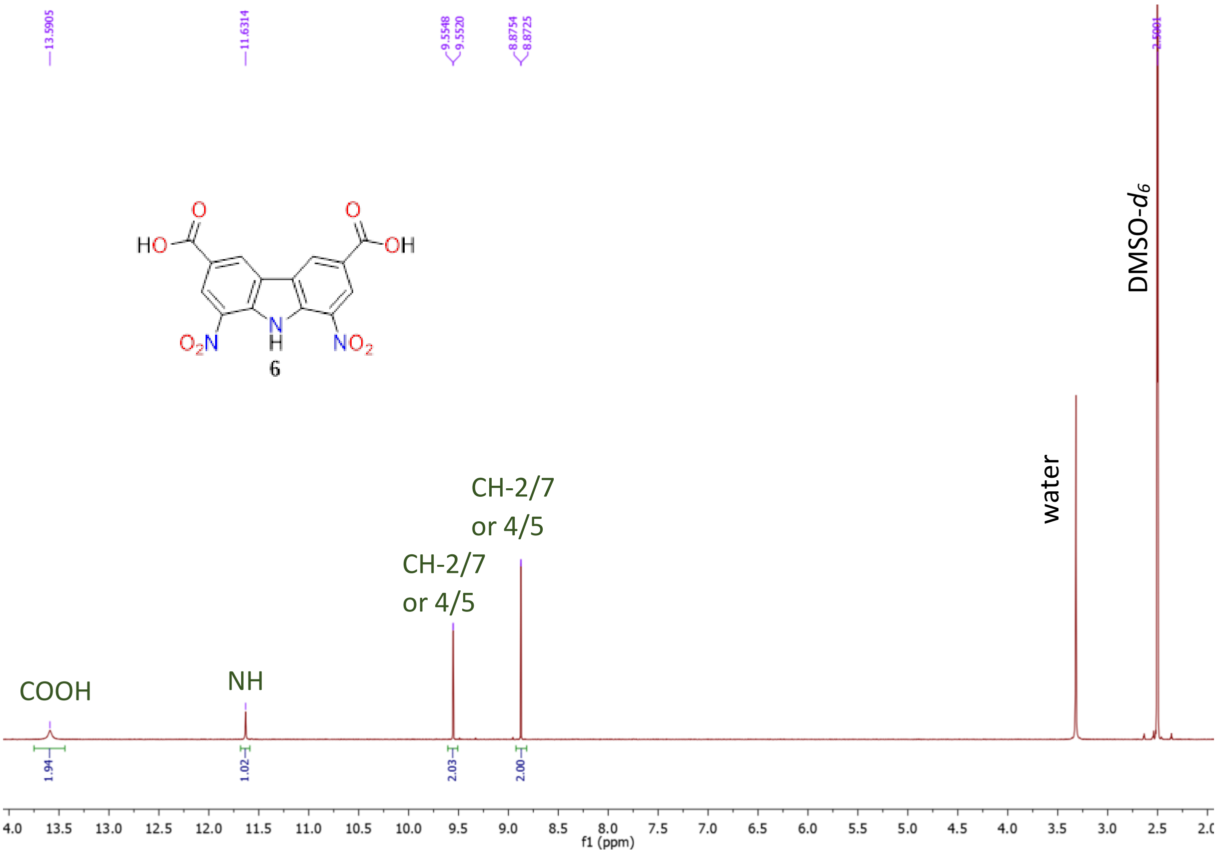

Figure S3. ^{1}H NMR spectrum of **6** in DMSO-d_6.

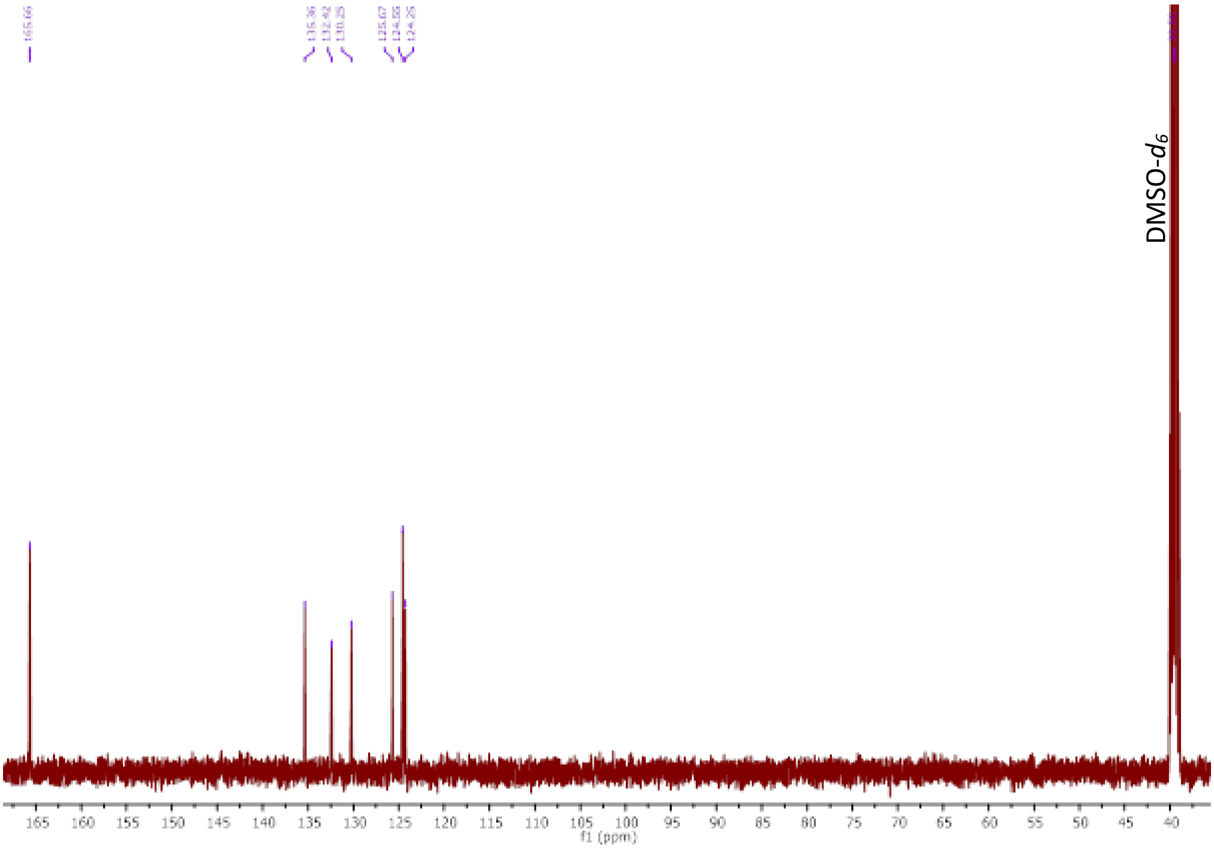

Figure S4. ^{13}C NMR spectrum of **6** in DMSO-d_6.

2.5 Compound **7**

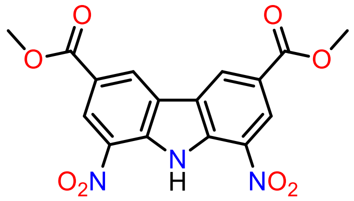

7

$C_{16}H_{11}N_3O_8$ = 373.27

Path A. Nitration of diester **4**

A 50 ml single-neck round-bottom flask equipped with a stirring bar was charged with a solution of diester **4** (405 mg, 1.43 mmol) in concentrated sulfuric acid (95%, 3.98 g, 38.6 mmol). The reaction mixture was stirred and cooled down in an ice bath. After 10 minutes, a cooled-down mixture of nitric acid (65%, 309 mg, 3.19 mmol) and sulfuric acid (95%, 2.52 g, 24.4 mmol) was added drop by drop (approx. one drop per 10 seconds). After the addition was complete, the reaction was quenched with ice (20 g) and mixed well. A precipitate formed, which was collected by filtration on a cold Schott filter funnel G4 and washed with deionized water (3×5 ml). After that the solid was dissolved in dichloromethane, and silica gel (3.33 g) was added to the solution. Volatiles were evaporated on a rotary evaporator and the solid residue was dried under high vacuum. Then, the pre-loaded mixture was separated by column chromatography on 115 g of silica gel, using dichloromethane as an eluent. Fractions containing pure product were combined and evaporated on a rotary evaporator to yield 16-137 mg (3-26 %) of pure **7** as yellow solid.

Path B. Esterification of diacid **6**

A 25 ml single-neck round-bottom flask equipped with a stirring bar was charged with diacid **6** (347 mg, 1.00 mmol), methanol (15 ml), and sulfuric acid (95%, 633 mg, 6.13 mmol). Then the flask was equipped with a reflux condenser. The reaction mixture was intensively stirred and heated to 95°C (temperature measured in oil bath) for 22 h. After this time the mixture was cooled down to room temperature, stored in a fridge overnight and filtrated on a Schott filter funnel G4. The obtained precipitate was washed with cold methanol (3×5 ml). The solid was purified by column chromatography on 40 g of silica gel, using dichloromethane as an eluent. Fractions containing pure product were combined and evaporated on a rotary evaporator. The solid was dried in a desiccator over KOH under high vacuum. Yield 291 mg (78 %) of pure product as a yellow solid.

^{1}H NMR (500 MHz, $CDCl_3$:DMSO-d_6 10:1) δ_{CDCl3}: 11.58 (s; 1H; NH); 9.09 (d; J = 1.4 Hz; 2H; CH-4/5 or CH-2/7); 9.03 (d; J = 1.4 Hz; 2H; CH4/5 or CH2/7); 3.96 (s; 6H, CH_3).

^{13}C NMR (126 MHz, $CDCl_3$:DMSO-d_6 10:1) δ_{CDCl3}: 164.85, 136.02, 132.79, 129.09, 125.20, 124.18, 52.82.

Elemental analysis: calcd. for $C_{16}H_{11}N_3O_8$: C, 51.48; H, 2.97; N, 11.26, found: C, 51.11; H, 2.93; N, 11.02.

HR MS (TOF MS ES$^-$) m/z calcd. for $C_{16}H_{10}N_3O_8^-$: 372.0468, found: 372.0464.

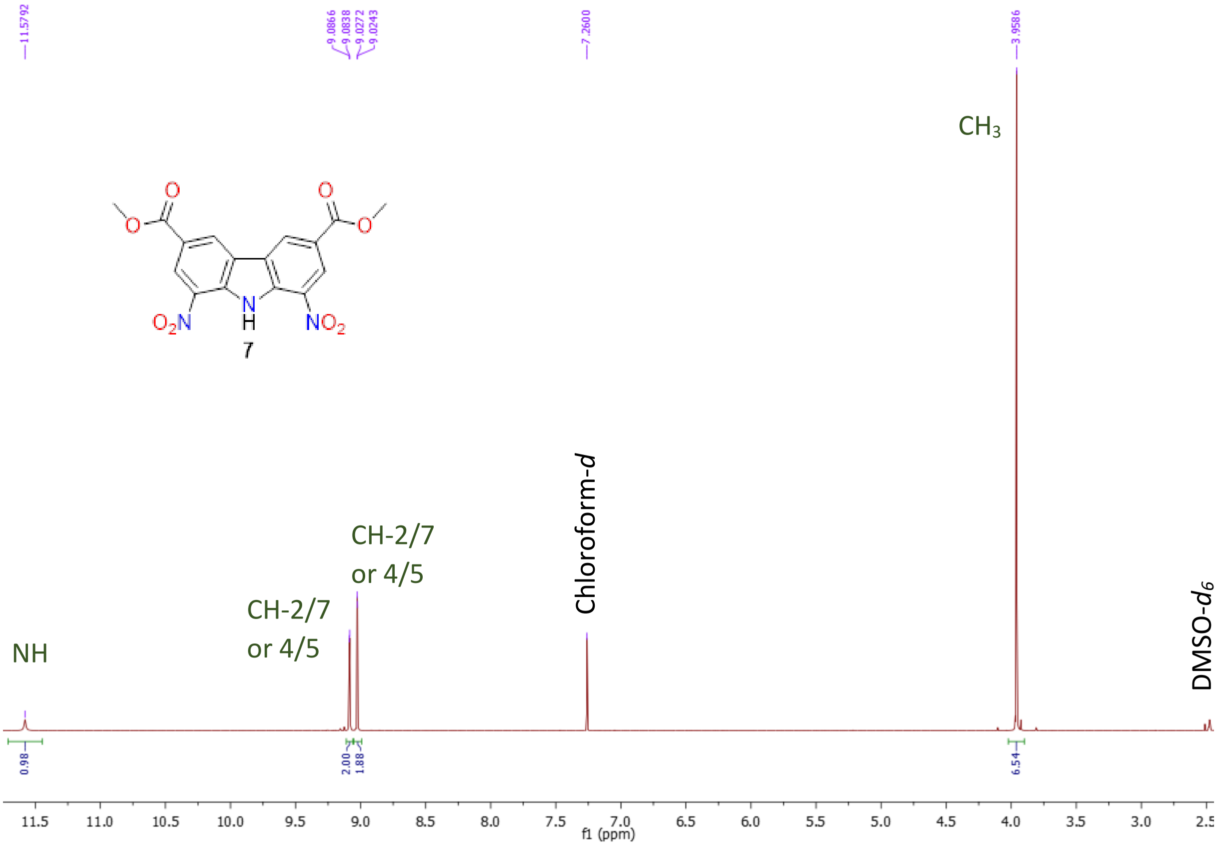

Figure S5. ^{1}H NMR spectrum of **7** in $CDCl_3$:DMSO-*d*$_6$=10:1. Chemical shifts are measured with respect to $CDCl_3$ = 7.26 ppm.

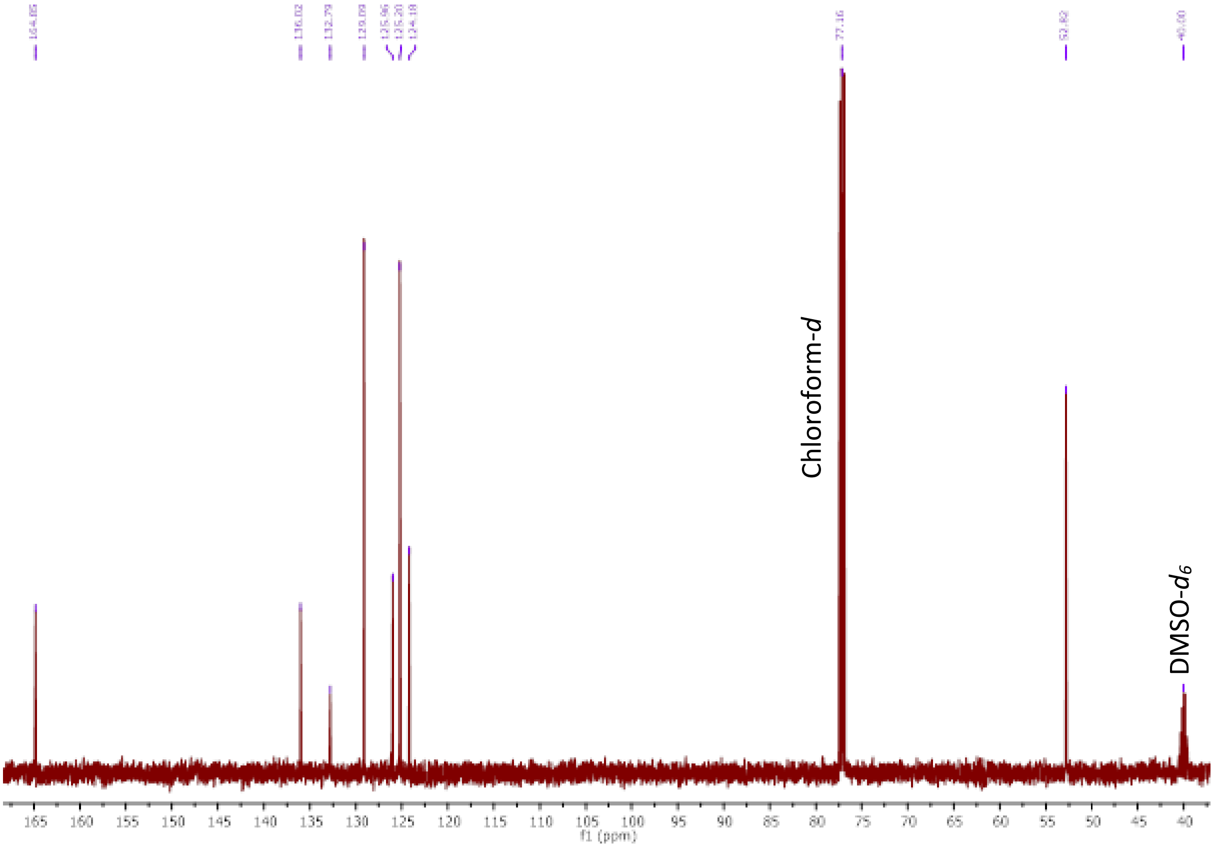

Figure S6. ^{13}C NMR spectrum of **7** in $CDCl_3$:DMSO-*d*$_6$=10:1. Chemical shifts are measured with respect to $CDCl_3$ = 77.16 ppm.

2.6 Compound **8**

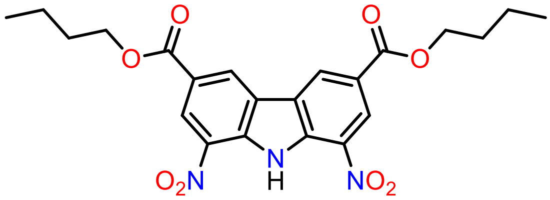

8

$C_{22}H_{23}N_3O_8$ = 457.43

A 25 ml single-neck round-bottom flask was charged with diacid **6** (346.1 mg, 1.00 mmol), n-butanol (10 ml), sulfuric acid (666.7 mg, 95%), and a stirring bar. Then the flask was equipped with a reflux condenser. The reaction mixture was intensively stirred and heated to 130°C (measured in oil bath) for 20 h. After this time the mixture was cooled down to room temperature, stored in a fridge, and filtrated through a Schott filter funnel G4. The obtained precipitate was washed with acetonitrile (3×5 ml). The solid was purified by column chromatography on silica gel (43 g) using gradient elution with 10% (500 ml), 30% (250 ml), and 50% (250 ml) of ethyl acetate in hexane. Fractions containing pure product were combined and evaporated on a rotary evaporator. The solid product was dried in a desiccator over KOH under high vacuum. Yield: 412.0 mg (90 %) of yellow solid.

^{1}H NMR (500 MHz, $CDCl_3$) δ_{CDCl3}: 11.70 (s; 1H; NH); 9.18 (dd; J_1 = 1.4 Hz; J_2 = 0.7 Hz, 2H; CH-4/5 or CH-2/7); 9.15 (d; J = 1.4 Hz ; 2H; CH-4/5 or CH-2/7); 4.47 (t, J = 6.7 Hz, 4H; butyl CH-1); 1.86 (m, 4H; butyl CH-2); 1.54 (m; 4H; butyl CH-3); 1.04 (t, J = 7.4 Hz, 6H; butyl CH-4).

^{13}C NMR (126 MHz, $CDCl_3$) δ_{CDCl3}: 164.80, 136.30, 133.08, 129.26, 126.21, 125.45, 124.84, 66.17, 30.93, 19.44, 13.94.

Elemental analysis: calcd. for $C_{22}H_{23}N_3O_8$: C, 57.76; H, 5.07; N, 9.19, found: C, 57.84; H, 5.13; N, 9.10.

HR MS (TOF MS ES$^-$) m/z calcd. for $C_{22}H_{22}N_3O_8^-$: 456.1407 found: 456.1395.

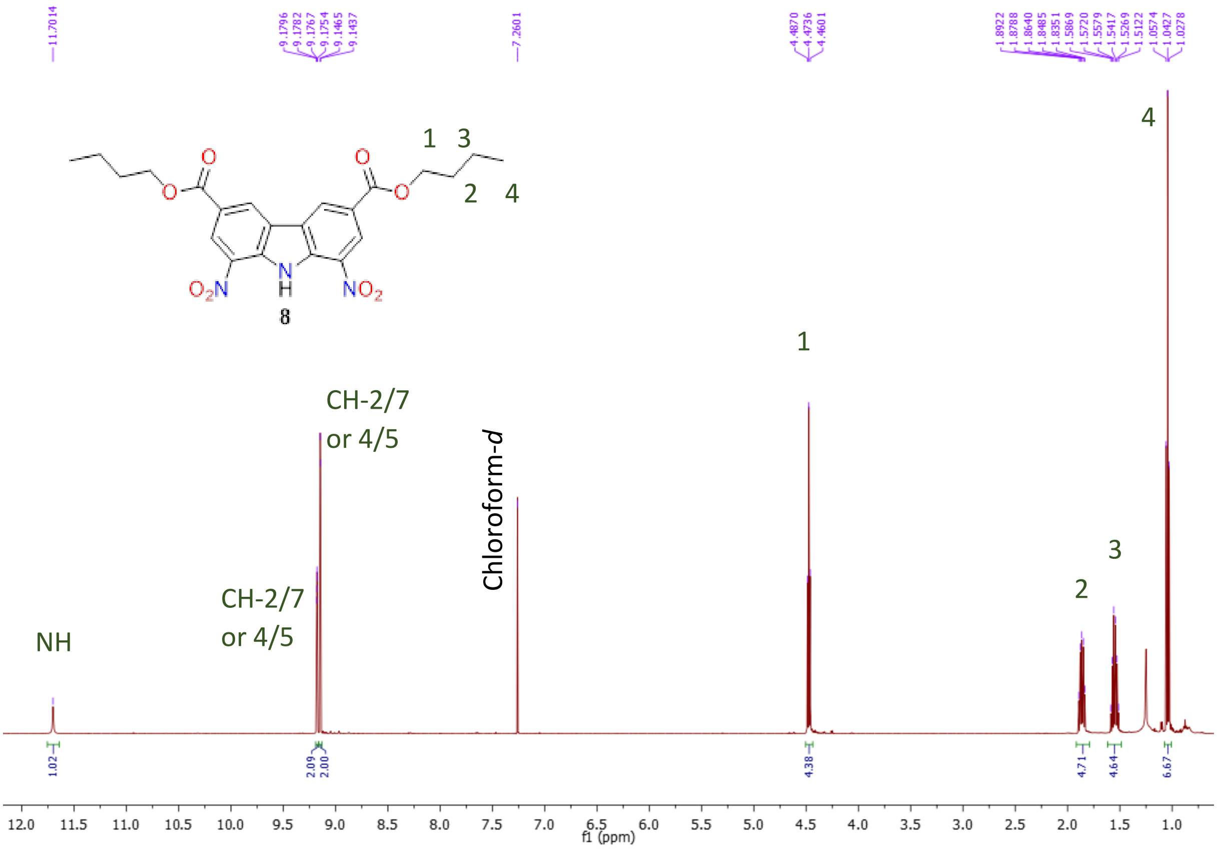

Figure S7. ^{1}H NMR spectrum of **8** in $CDCl_3$.

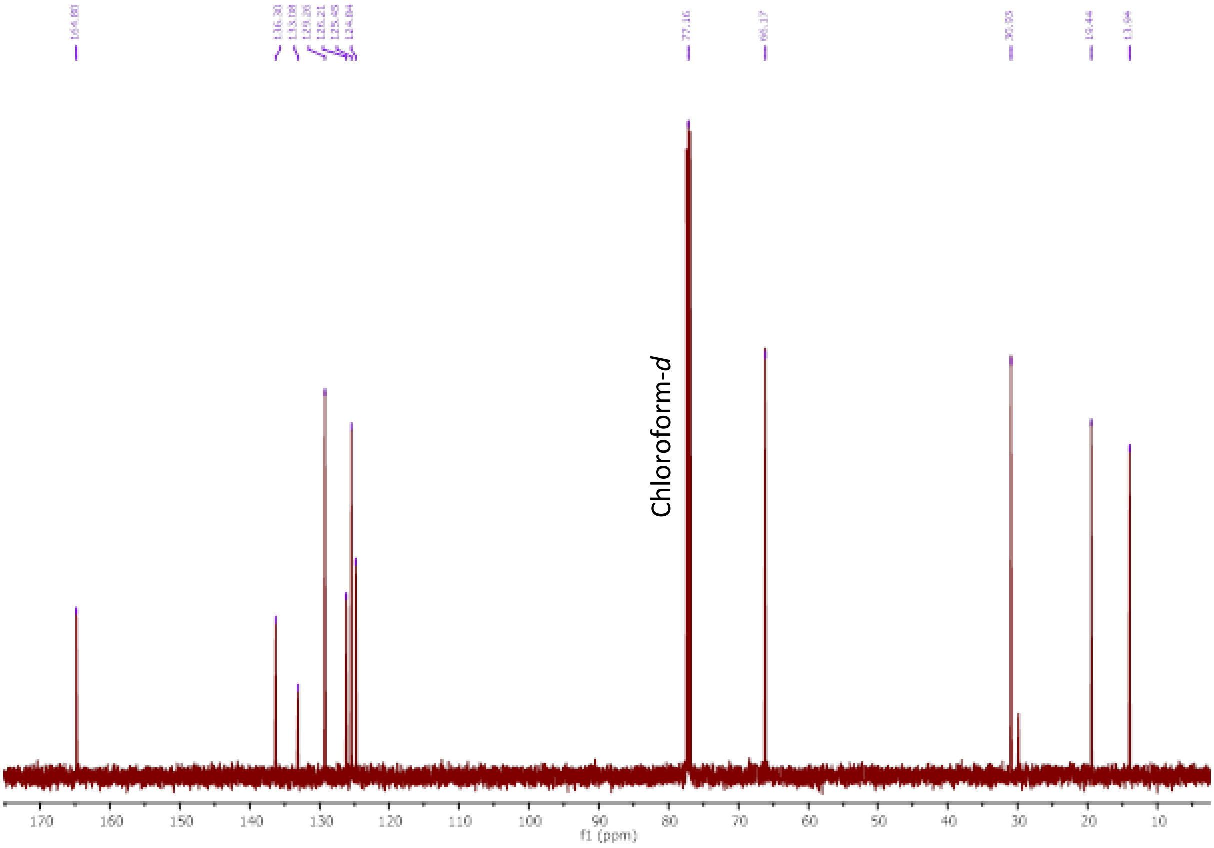

Figure S8. ^{13}C NMR spectrum of **8** in $CDCl_3$.

2.7 Compound **9**

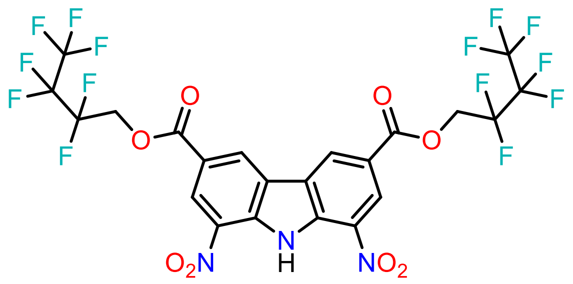

9

$C_{22}H_9F_{14}N_3O_8$ = 709.30

A 10 ml single-neck round-bottom flask was charged with diacid **6** (346 mg, 1.00 mmol) and equipped with a stirring bar. The flask was sealed with a septum and deaerated by sequentially evacuating and refilling with argon three times. Thionyl chloride (3 ml) and three droplets of DMF were added in the flow of argon. Then, still in the flow of argon, the flask was equipped with a reflux condenser connected to a bubbler and the reaction mixture was heated to 60°C (temperature measured in oil bath) with intense stirring for 4 h. After this time the volatiles were evaporated on a rotary evaporator and the solid residue **6b** was further dried under high vacuum. Next, the flask was sealed with a septum and deaerated as before. DCM (3 ml) was added through the septum and the flask was put into an ultrasonic bath for approx. 1 minute. Triethylamine (0.40 ml) and 2,2,3,3,4,4,4-heptafluorobutyl alcohol (0.40 ml, 3.20 mmol) were added. The reaction mixture was intensively stirred for 4h. Then the volatiles were removed on a rotary evaporator and the solid residue was purified by column chromatography using CombiFlash instrument, 4 g cartridge, and eluent's flow of 15 ml/min. Separation was achieved using gradient elution with ethyl acetate in hexane 0->10% (5 min), 10% (20 min), 10->25% (5 min), and 25% (60 min). Fractions containing pure product were combined and evaporated on a rotary evaporator. The solid was dried in a desiccator over KOH under high vacuum. Yield 604 mg (85 %) of yellow solid.

^{1}H NMR (500 MHz, $(CD_3)_2CO$) $\delta_{acetone}$: 11.73 (s; 1H; NH); 9.55 (d; *J* = 1.5 Hz; 2H; CH4/5 or CH2/7); 9.06 (d; *J* = 1.5 Hz ; 2H; CH4/5 or CH2/7); 3.41 (m, 4H; CH_2).

^{19}F NMR (470 MHz, $(CD_3)_2CO$) $\delta_{acetone}$: -81.71 (t; *J* = 9.4 Hz; 6F); -120.66 (m, 4F); -128.10 (m, 4F).

^{13}C NMR (126 MHz, $(CD_3)_2CO$) $\delta_{acetone}$: 206.14, 163.95, 134.17, 131.21, 127.28, 125.90, 123.28, 60.97. The remaining aliphatic carbons were barely visible due to coupling with fluorine atoms.

Elemental analysis: calcd. for $C_{22}H_9F_{14}N_3O_8$: C, 37.25; H, 1.28; F, 37.50; N, 5.92, found: C, 37.17; H, 1.28; F, 37.60; N, 5.94.

HR MS (TOF MS ES$^-$) m/z calcd. for $C_{22}H_8F_{14}N_3O_8^-$: 708.0088 found: 708.0076.

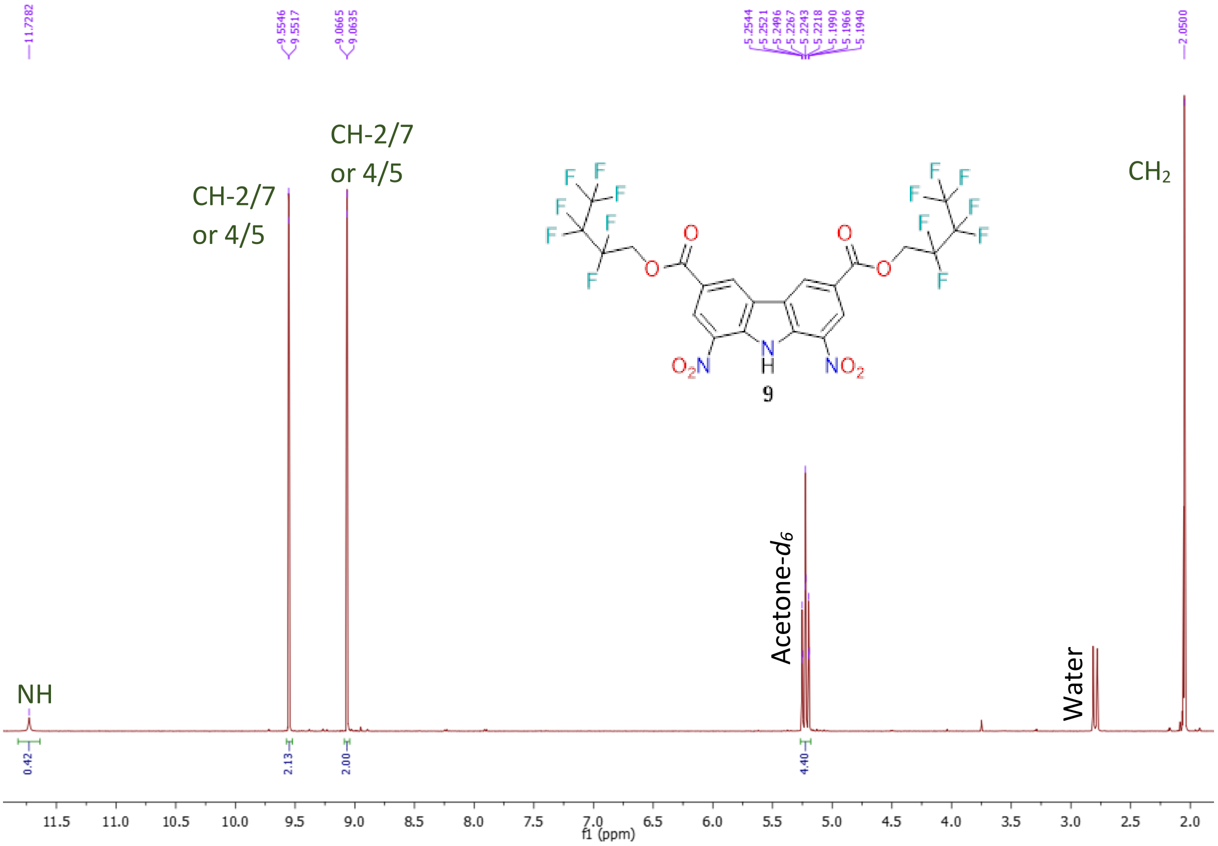

Figure S9. ^{1}H NMR spectrum of **9** in acetone-d_6.

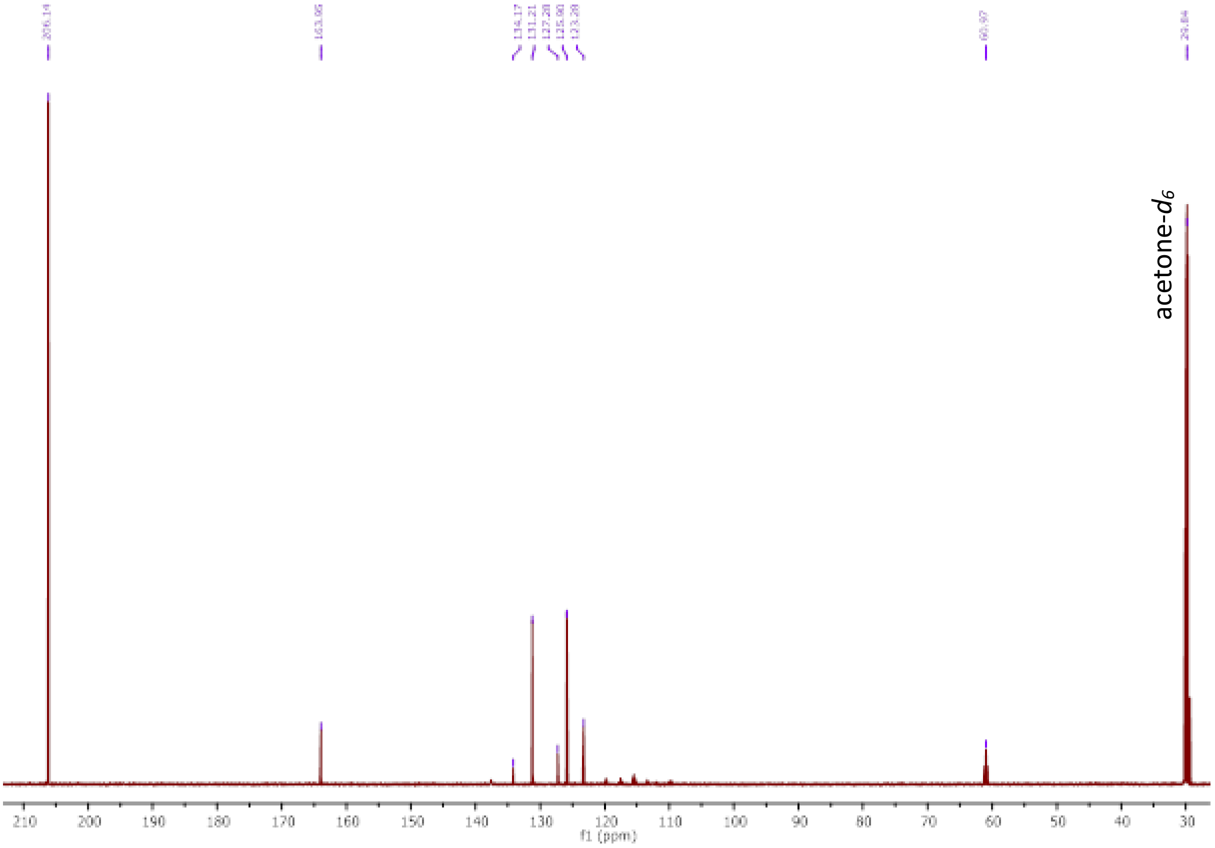

Figure S10. ^{13}C NMR spectrum of **9** in acetone-d_6.

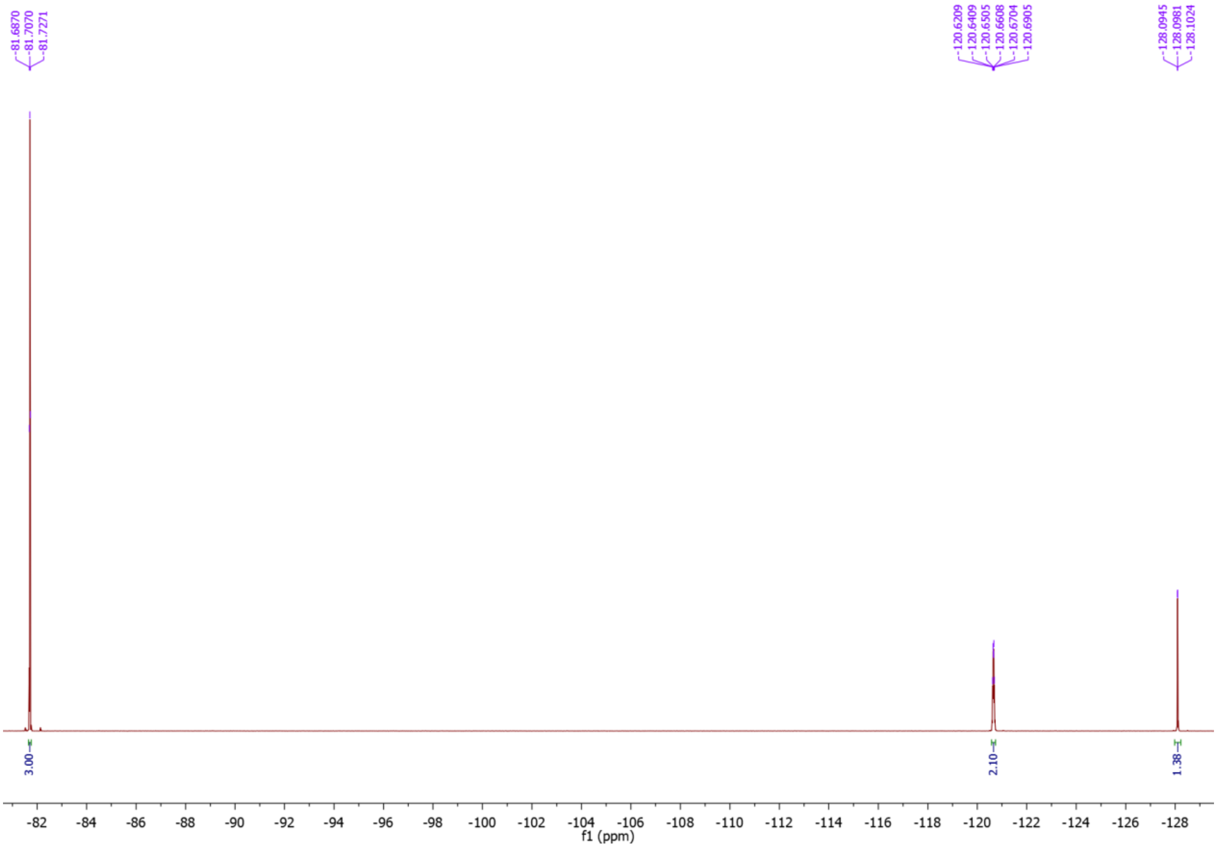

Figure S11. ^{19}F NMR spectrum of **9** in acetone-d_6.

2.8 Compound **10**

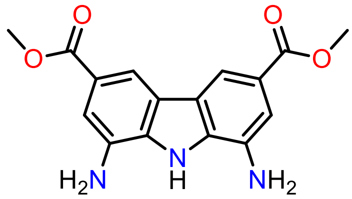

10

$C_{16}H_{15}N_3O_4$ = 313.31

A 25 ml two-neck round-bottom flask was charged with dinitro compound **7** (176 mg, 0.472 mmol) and equipped with a stirring bar. The flask was sealed with septa and deaerated by sequentially evacuating and refilling with argon three times. Then acetonitrile (5 ml) and 10% Pd/C (15.6 mg) were added in a flow of argon. The argon flow was changed to hydrogen and maintained for 30 minutes. To wash out the compounds deposited on the flask walls due to bubbling of hydrogen, another 5 ml of acetonitrile was added. The pressure of hydrogen was set to 0.3 bar and the mixture was intensively stirred for 7 hours. After this time DMSO (5 ml) was added *via* a syringe to dissolve the obtained product and the reaction mixture was filtrated using 0.2 µm PTFE filter under argon atmosphere. Deoxygenated water (50 ml) was added immediately, in the flow of argon, to yield a pale red precipitation. The solid was filtered out on a Schott filter funnel G4 and dried in a desiccator over KOH under high vacuum. Yield 121 mg (77 %) of brown solid. To obtain analytically pure sample for analyses, compound **12** was purified by column chromatography with 5% MeOH in DCM as an eluent.

^{1}H NMR (500 MHz, DMSO-d_6) δ_{DMSO}: 11.15 (s; 1H; NH); 8.07 (d; *J* = 1.4 Hz; 2H; CH4/5 or CH2/7); 7.36 (d; *J* = 1.5 Hz ; 2H; CH4/5 or CH2/7); 5.34 (s, 4H; NH_2); 3.85 (s, 6H; CH_3).

^{13}C NMR (126 MHz, DMSO-d_6) δ_{DMSO}: 167.27, 133.76, 132.05, 123.10, 121.72, 111.54, 109.98, 51.62.

HR MS (TOF MS ES$^+$) m/z calcd. for $C_{16}H_{16}N_3O_4^+$: 314.1141, found: 314.1129.

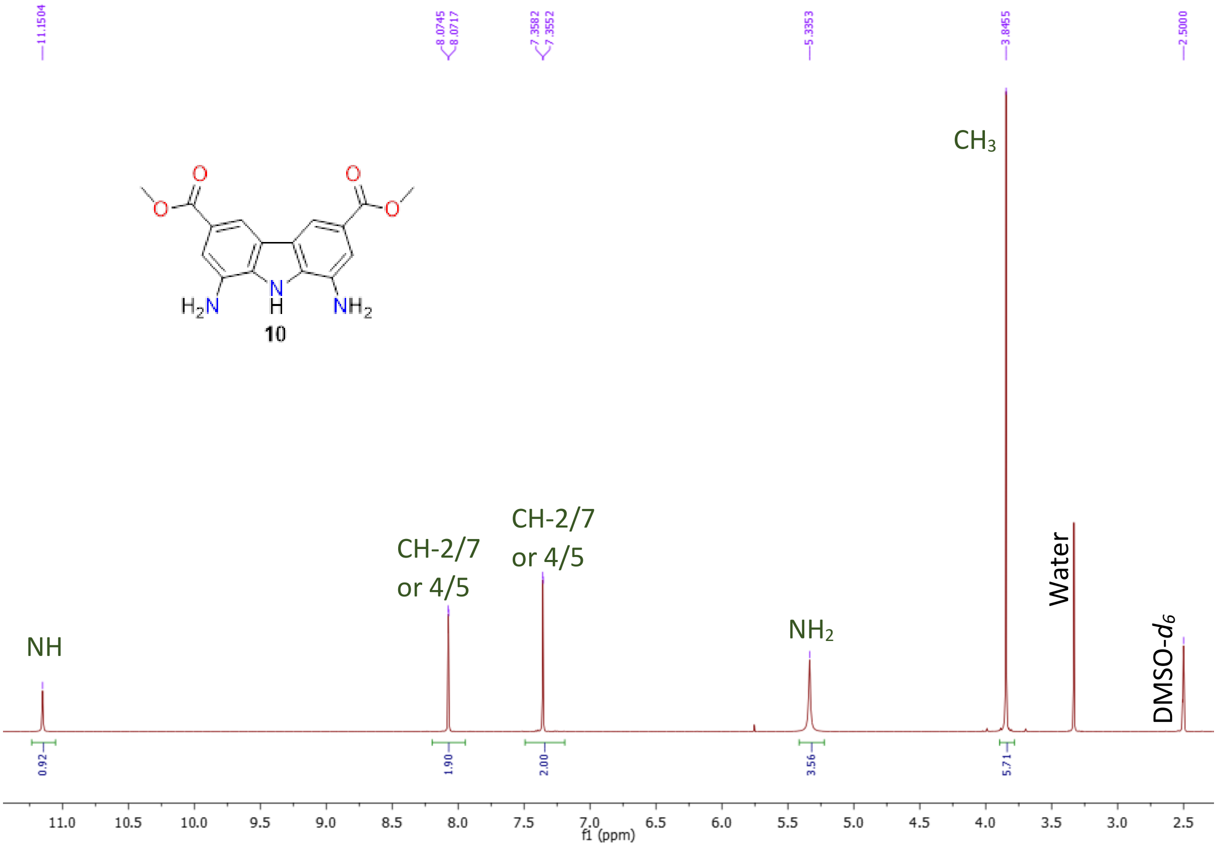

Figure S12. ^{1}H NMR spectrum of **10** in DMSO-d_6.

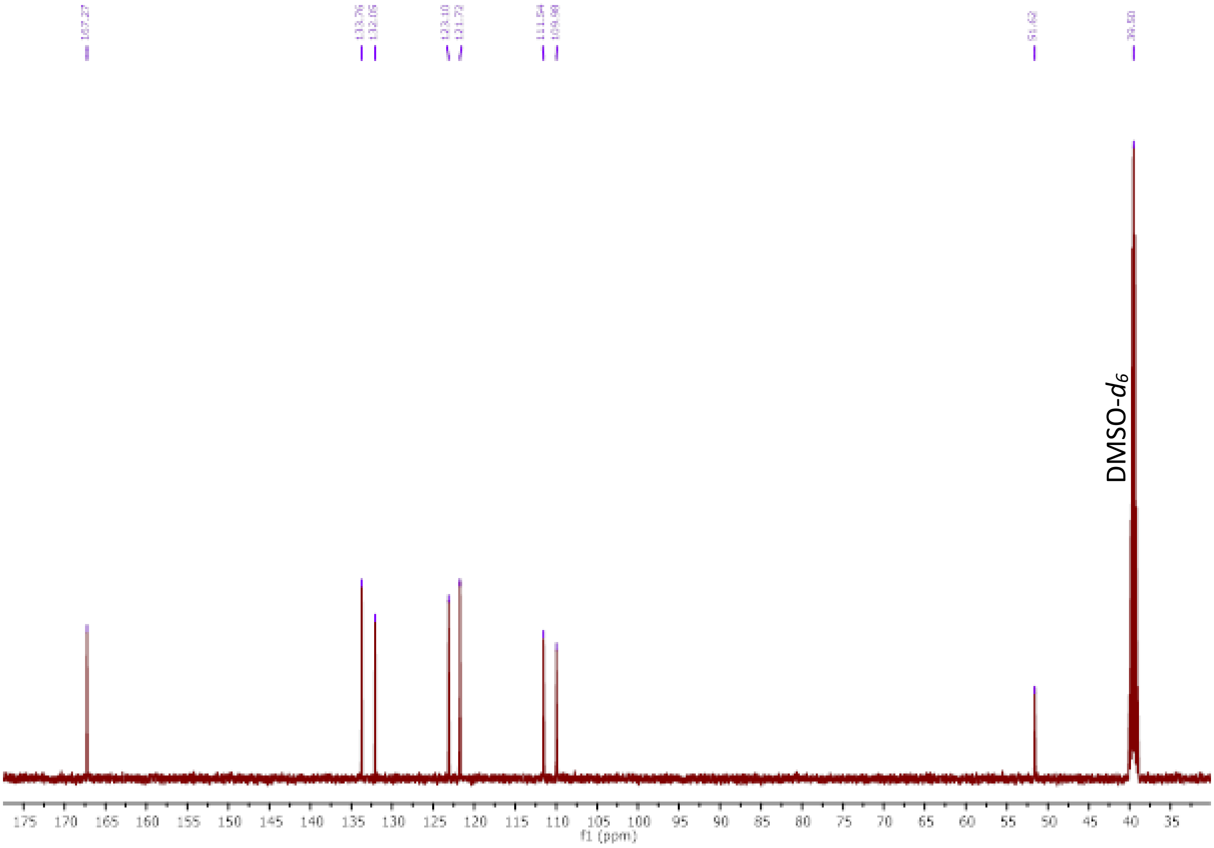

Figure S13. ^{13}C NMR spectrum of **10** in DMSO-d_6.

2.9 Compound **11**

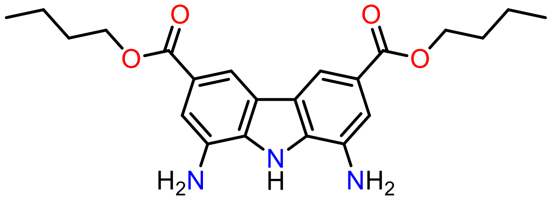

11

$C_{22}H_{27}N_3O_4$ = 397.47

A 25 ml two-neck round-bottom flask was charged with the dinitro compound **8** (229 mg, 0.501 mmol) and equipped with a stirring bar. The flask was sealed with septa and deaerated by sequentially evacuating and refilling with argon three times. Then acetonitrile (5 ml) and 10% Pd/C (15.3 mg) were added in a flow of argon. The argon flow was changed to hydrogen and maintained for 30 minutes. To wash out the compounds deposited on the flask walls due to bubbling of hydrogen, another 5 ml of acetonitrile was added. The pressure of hydrogen was set to 0.3 bar and the mixture was intensively stirred for 5 hours. After this time DMF (10 ml) was added *via* a syringe to dissolve the obtained product and the reaction mixture was filtrated using 0.2 µm PTFE filter under argon atmosphere. Deoxygenated water (50 ml) was added immediately, in the flow of argon, to yield a white precipitation. The solid was filtered out on a Schott filter funnel G4 and dried in a desiccator over KOH under high vacuum. Yield 176 mg (89 %) of white solid.

^{1}H NMR (500 MHz, DMSO-d_6) δ_{DMSO}: 11.13 (s; 1H; NH); 8.05 (d; J = 1.4 Hz, 2H; CH4/5 or CH2/7); 7.35 (d; J = 1.5 Hz ; 2H; CH4/5 or CH2/7); 5.33 (s, 4H, NH_2); 4.27 (t; J_1 = 6.6 Hz; J_2 = 6.6 Hz, 4H); 1.72 (m, 4H); 1.46 (m, 4H); 0.96 (t; J_1 = 7.4 Hz; J_2 = 7.4 Hz, 6H; CH-3).

^{13}C NMR (126 MHz, DMSO-d_6) δ_{DMSO}: 166.85, 133.73, 132.04, 123.07, 122.02, 111.43, 109.96, 63.77, 30.48, 18.85, 13.67.

Elemental analysis: calcd. for $C_{22}H_{27}N_3O_4$: C, 66.48; H, 6.85; N, 5.10.57, found: C, 66.57; H, 6.95; N, 10.32.

HR MS (TOF MS ES$^-$) m/z calcd. for $C_{22}H_{26}N_3O_4^-$: 396.1923 found: 396.1918.

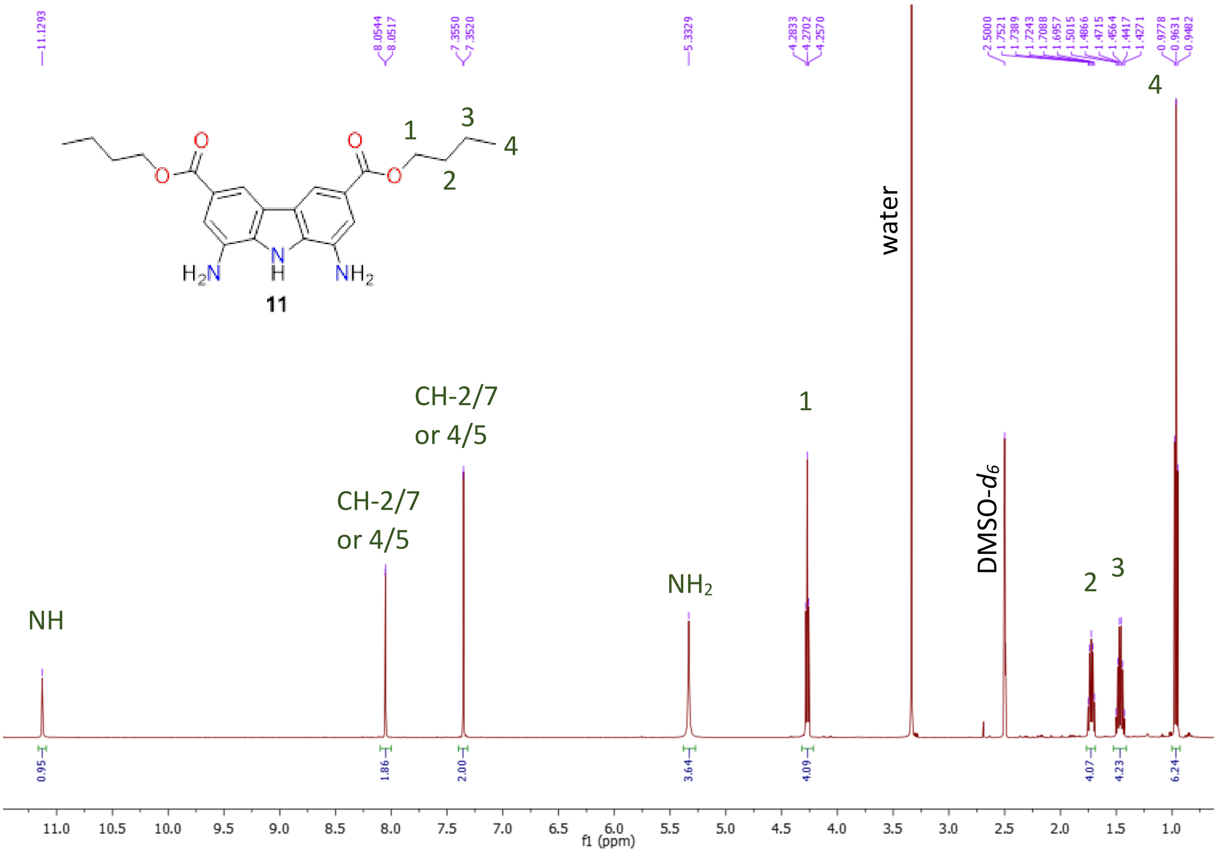

Figure S14. ^{1}H NMR spectrum of **11** in DMSO-d_6.

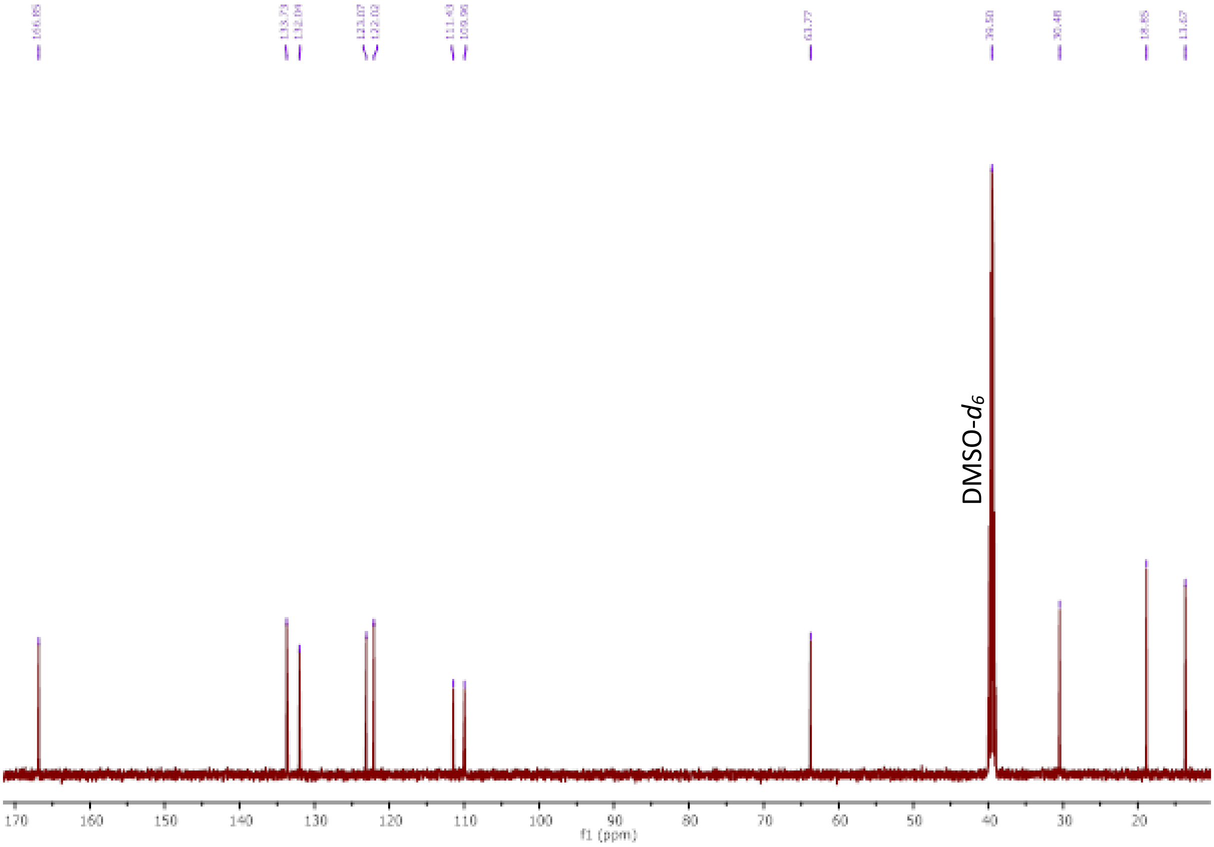

Figure S15. ^{13}C NMR spectrum of **11** in DMSO-d_6.

2.10 Compound **12**

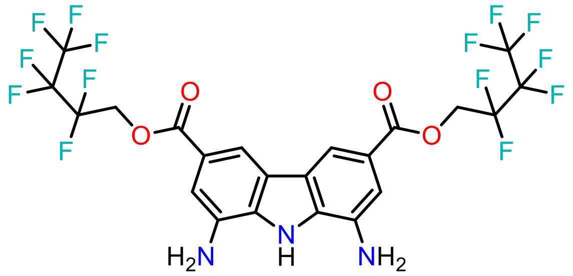

12

$C_{22}H_{13}F_{14}N_3O_4$ = 649.33

A 25 ml two-neck round-bottom flask was charged with dinitro compound **9** (355 mg, 0.500 mmol) and equipped with a stirring bar. The flask was sealed with septa and deaerated by sequentially evacuating and refilling with argon three times. Then acetonitrile (5 ml) and 10% Pd/C (16.4 mg) were added in a flow of argon. The argon flow was changed to hydrogen and maintained for 30 minutes. To wash out the compounds deposited on the flask walls due to bubbling of hydrogen, another 5 ml of acetonitrile was added. The pressure of hydrogen was set to 0.3 bar and the mixture was intensively stirred for 5 hours. After this time DMF (10 ml) was added *via* a syringe to dissolve the obtained product and the reaction mixture was filtrated using 0.2 µm PTFE filter under argon atmosphere. Deoxygenated water (50 ml) was added immediately, in the flow of argon, to yield a white precipitation. The solid was filtered out on a Schott filter funnel G4 and dried in a desiccator over KOH under high vacuum. Yield 281 mg (87 %) of white solid.

^{1}H NMR (500 MHz, DMSO-*d*$_6$) δ_{DMSO}: 11.34 (s; 1H; NH); 8.06 (d; *J* = 1.4 Hz, 2H; CH4/5 or CH2/7); 7.37 (d; *J* = 1.55 Hz; 2H; CH4/5 or CH2/7); 5.47 (s, 4H, NH_2); 5.10 (t; *J* = 13.9 Hz, 4H; CH_2).

^{13}C NMR (126 MHz, DMSO-*d*$_6$) δ_{DMSO}: 165.06, 134.31, 132.61, 122.99, 119.98, 111.69, 109.97, 59.03. The remaining aliphatic carbons were barely visible due to coupling with fluorine atoms.

^{19}F NMR (470 MHz, DMSO-*d*$_6$) δ_{DMSO}: -80.51 (t; *J* = 9.0 Hz; 6F); -119.13 (m, 4F); -127.09 (m, 4F)

Elemental analysis: calcd. for $C_{22}H_{13}F_{14}N_3O_4$: C, 40.69; H, 2.02; F, 40.96; N, 6.47, found: C, 40.84; H, 2.19; F, 40.94; N, 6.37.

HR MS (TOF MS ES$^+$) m/z calcd. for $C_{22}H_{14}F_{14}N_3O_4^+$: 650.0761 found: 650.0746.

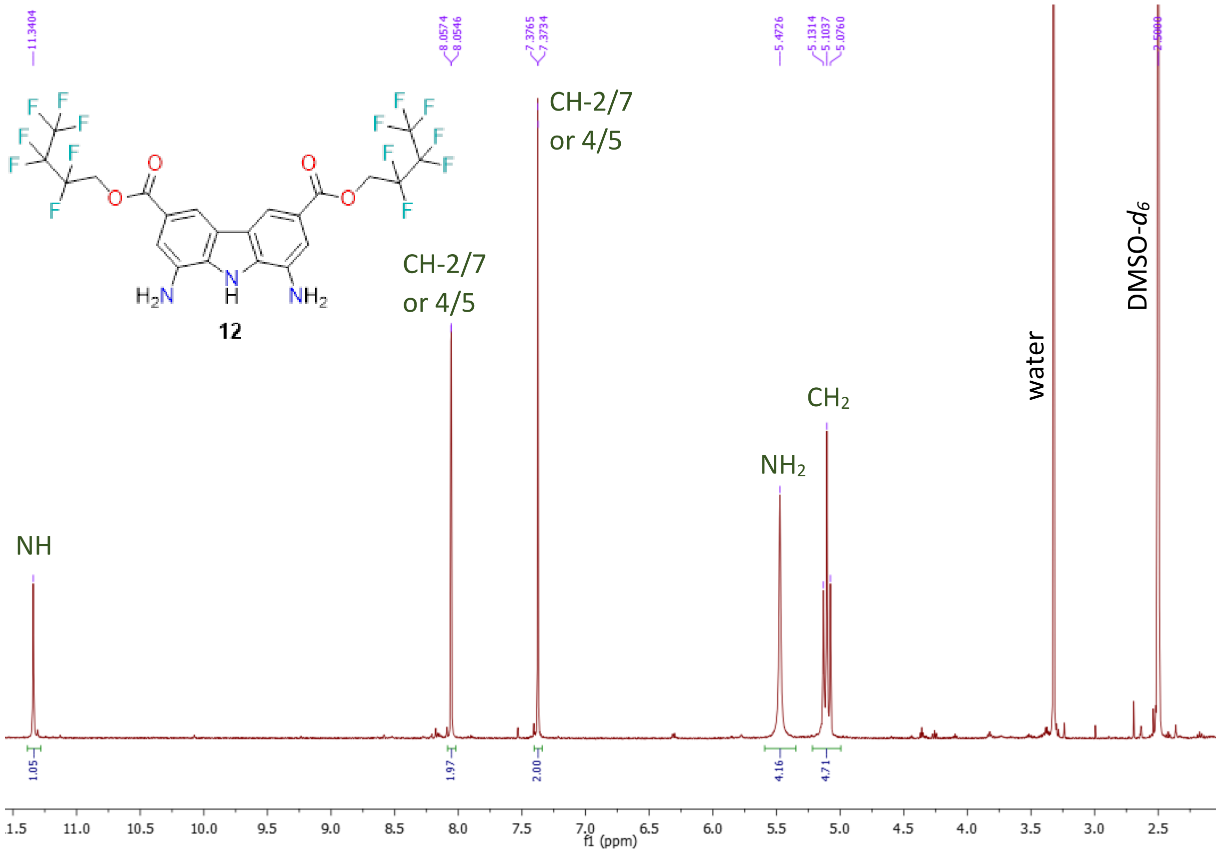

Figure S16. ^{1}H NMR spectrum of **12** in DMSO-d_6.

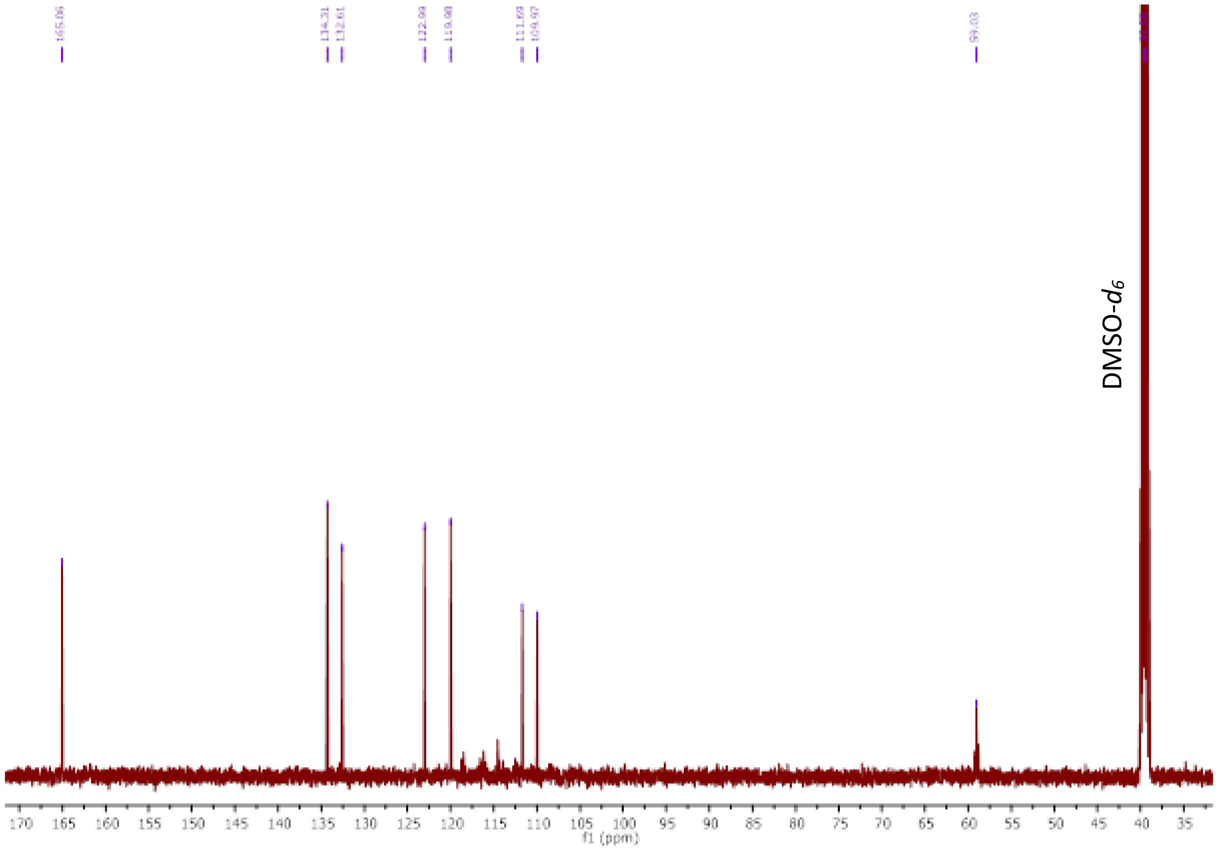

Figure S17. ^{13}C NMR spectrum of **12** in DMSO-d_6.

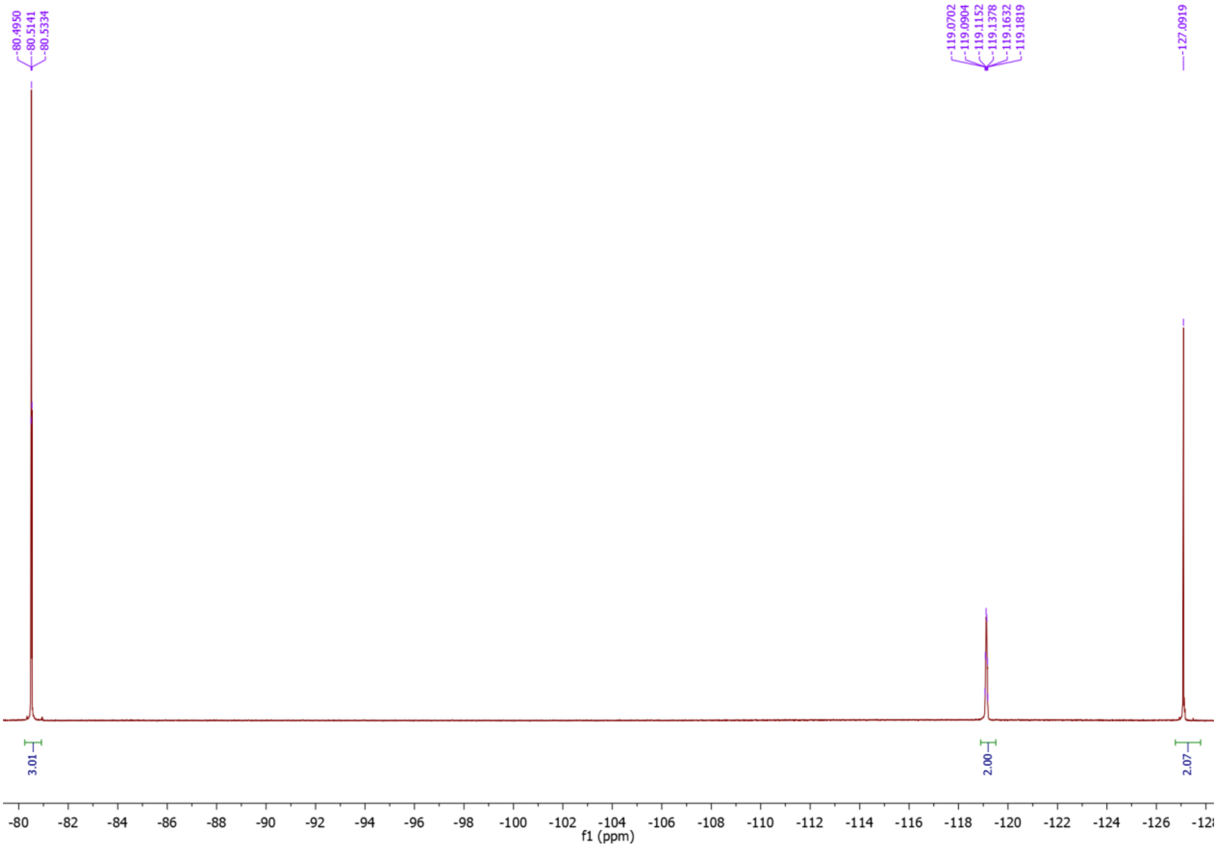

Figure S18. ^{19}F NMR spectrum of **12** in DMSO-d_6.

2.11 Receptor **R1**

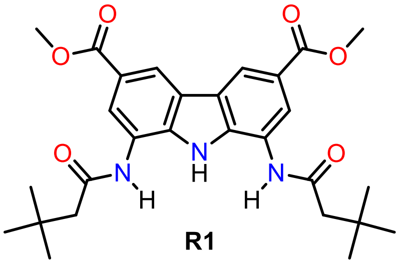

R1

$C_{28}H_{35}N_3O_6$ = 509.59

A 50 ml single-neck round-bottom flask was charged with diamine **10** (94.8 mg, 0.300 mmol) and equipped with a stirring bar. The flask was sealed with a septum and deaerated by sequentially evacuating and refilling with argon three times. Then dry THF (20 ml) was added in a flow of argon, followed by triethylamine (0.09 ml, 67 mg, 0.66 mmol) and *t*-butylacetyl acid chloride (0.09 ml, 89 mg, 0.66 mmol). The reaction mixture was intensively stirred for 1h. After this time the volatiles were evaporated on a rotary evaporator. The solid residue was suspended in water (5 ml), filtered through a Schott filter funnel G4 and then further washed with water (2 × 5 ml) and diethyl ether (3 × 5 ml). The product was dried in a desiccator over KOH under high vacuum. Yield 101 mg (66 %) of white solid.

^{1}H NMR (500 MHz, DMSO-*d*$_6$) δ_{DMSO}: 10.90 (s; 1H; carbazole NH); 10.20 (s; 2H; amide NH); 8.74 (s; 2H; CH4/5 or CH2/7); 8.15 (s; 2H; CH4/5 or CH2/7); 3.91 (s, 6H, CH_3); 2.36 (s; 4H; CH_2); 1.11 (s; 18H; *t*-butyl).

^{13}C NMR (126 MHz, DMSO-*d*$_6$) δ_{DMSO}: 170.51, 166.42, 135.32, 124.16, 123.26, 121.53, 120.33, 119.05, 51.99, 49.02, 30.91, 29.66.

HR MS (TOF MS ES$^+$) m/z calcd. for $C_{28}H_{35}N_3O_6Na^+$: 532.2424 found: 532.2417.

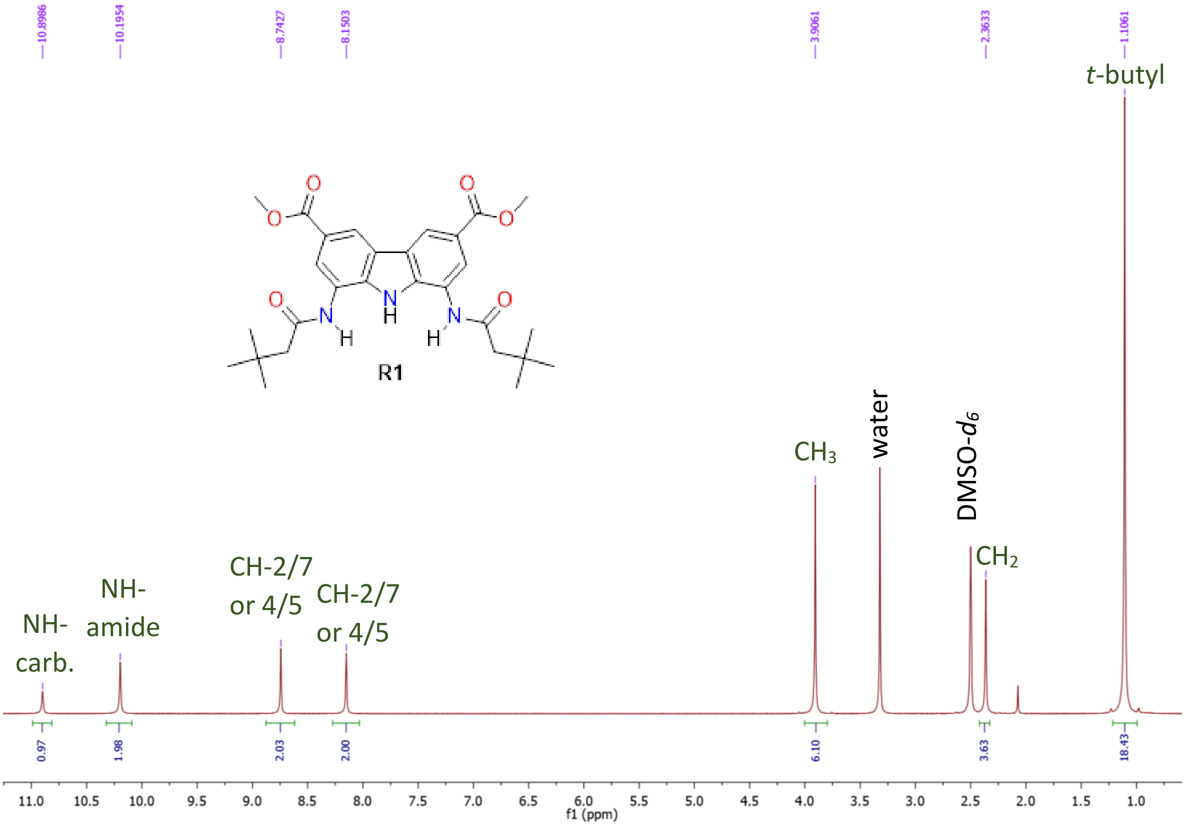

Figure S19. ^{1}H NMR spectrum of **R1** in DMSO-*d*$_6$.

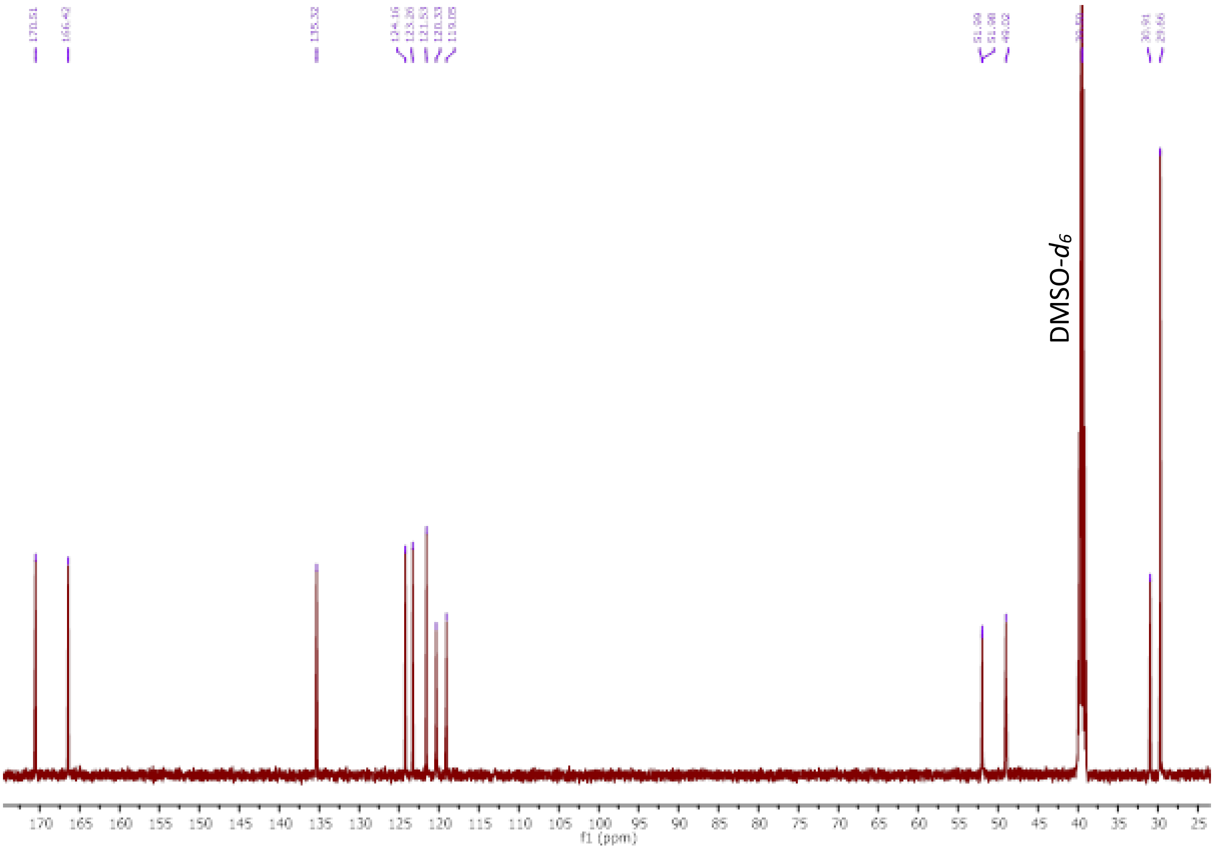

Figure S20. ^{13}C NMR spectrum of **R1** in DMSO-d_6.

2.12 Receptor **R2**

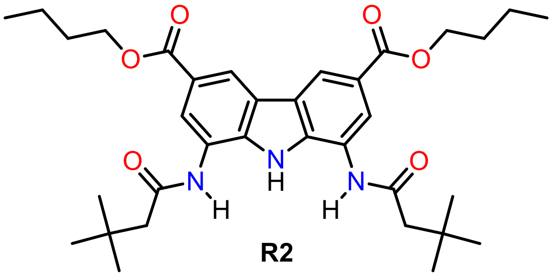

R2

$C_{34}H_{47}N_3O_6$ = 593.35

A 50 ml single-neck round-bottom flask was charged with diamine **11** (117 mg, 0.300 mmol) and equipped with a stirring bar. The flask was sealed with a septum and deaerated by sequentially evacuating and refilling with argon three times. Then dry THF (20 ml) was added in a flow of argon, followed by triethylamine (0.09 ml, 67 mg, 0.66 mmol) and *t*-butylacetic acid chloride (0.09 ml, 89 mg, 0.66 mmol). After this time the volatiles were evaporated on a rotary evaporator. The solid residue was suspended in diethyl ether (5 ml), filtered through a Schott filter funnel G4 and then further washed with diethyl ether (2 × 5 ml) and MeOH (3 × 5 ml). The solid was dried in a desiccator over KOH under high vacuum. Yield 99.7 mg (56 %) of white solid**.**

^{1}**H NMR** (500 MHz, DMSO-d_6) δ_{DMSO}: 10.85 (s; 1H; carbazole NH); 10.21 (s, 2H, amide NH); 8.72 (s; 2H; CH4/5 or CH2/7); 8.11 (s, 2H; CH4/5 or CH2/7); 4.33 (t; *J* = 6.6 Hz; 4H); 2.36 (s, 4H); 1.76 (m, 4H); 1.48 (m, 4H); 1.11 (s, 18H, *t*-butyl); 0.97 (m, 6H, CH_3).

^{13}**C NMR** (126 MHz, DMSO-d_6) δ_{DMSO}: 170.50, 165.99, 135.37, 124.18, 123.26, 121.80, 120.38, 118.97, 64.24, 49.00, 30.90, 30.41, 29.66, 18.79, 13.65.

HR MS (TOF MS ES$^-$) m/z calcd. for $C_{34}H_{47}N_3O_6Na^+$: 616.3363 found: 616.3350.

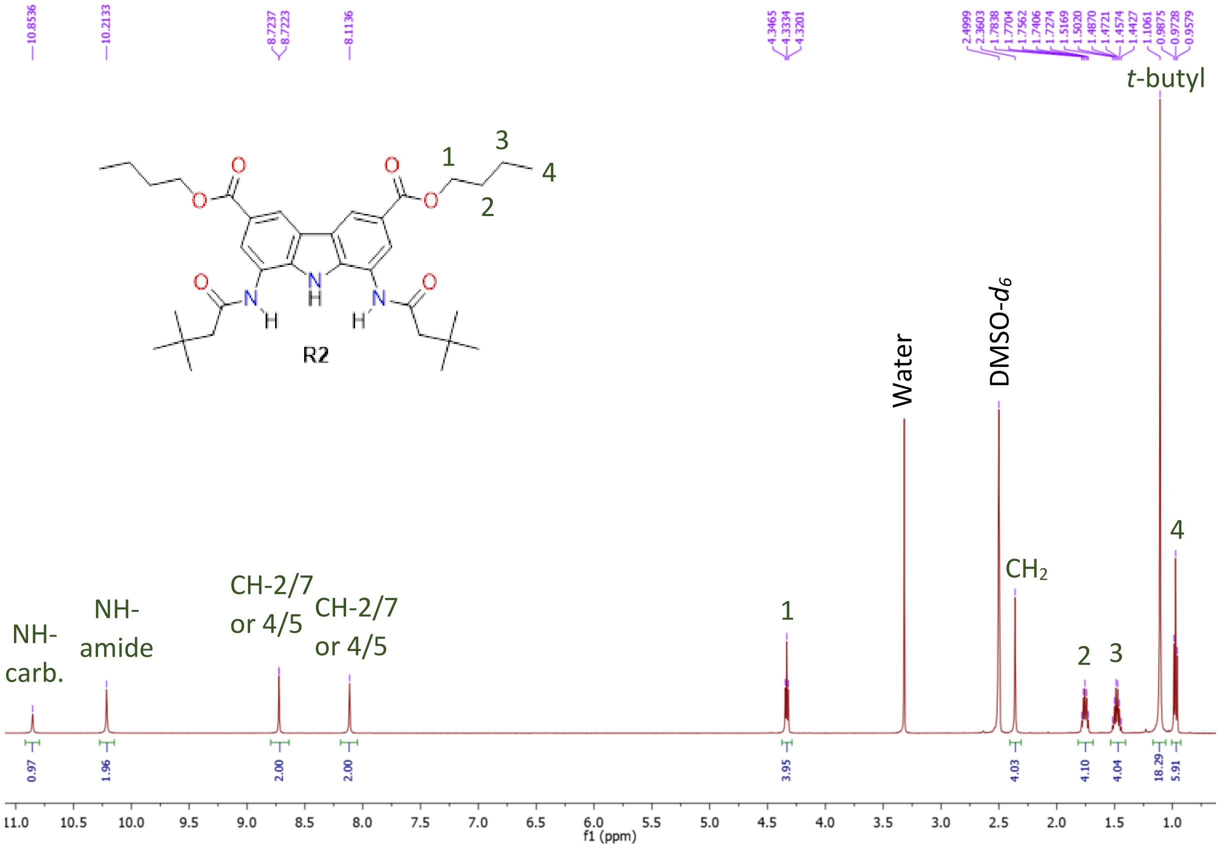

Figure S21. ^{1}H NMR spectrum of **R2** in DMSO-d_6.

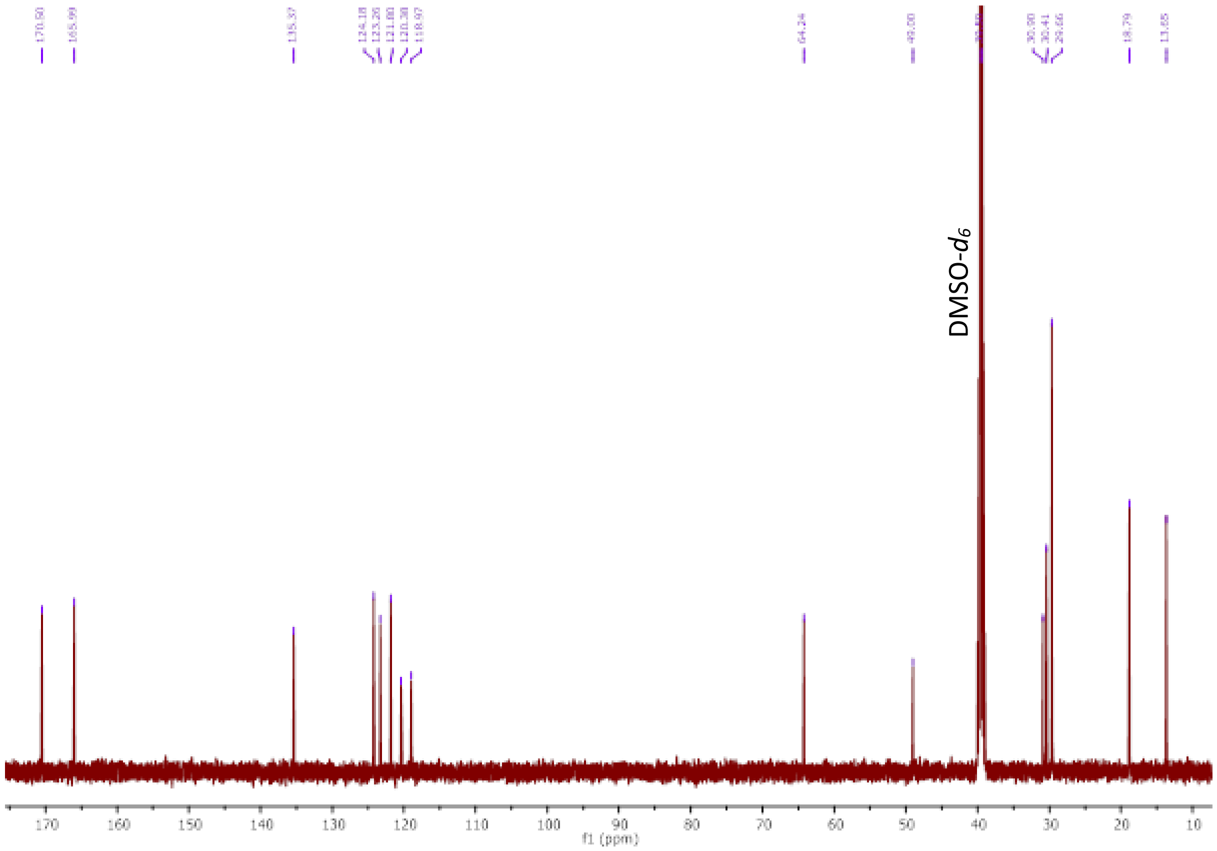

Figure S22. ^{13}C NMR spectrum of **R2** in DMSO-d_6.

2.13 Receptor **R3**

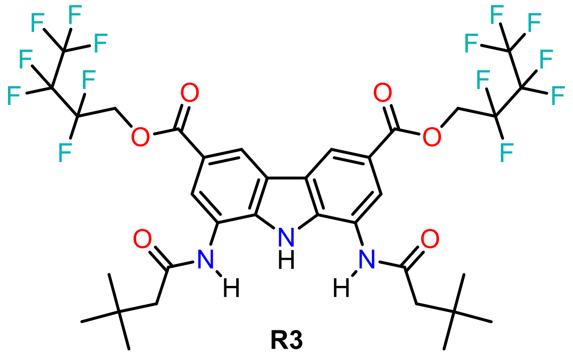

R3

$C_{34}H_{33}F_{14}N_3O_6$ = 845.21

A 50 ml single-neck round-bottom flask was charged with diamine **12** (147 mg, 0.300 mmol) and equipped with a stirring bar. The flask was sealed with a septum and deaerated by sequentially evacuating and refilling with argon three times. Then dry THF (20 ml) was added in a flow of argon, followed by triethylamine (0.09 ml, 67 mg, 0.66 mmol) and *t*-butylacetic acid chloride (0.09 ml, 89 mg, 0.66 mmol). The reaction mixture was intensively stirred for 1h. After this time the volatiles were evaporated on a rotary evaporator. To the solid was added 5 ml of water and then washed on a Schott filter funnel G4 with water (2 × 5 ml) and diethyl ether (3 × 5 ml). The solid was dried in desiccator over KOH under high vacuum. Yield 185 mg (73%) of white solid.

^{1}H NMR (500 MHz, DMSO-*d$_6$*) δ_{DMSO}: 11.10 (s; 1H; carbazole NH); 10.25 (s, 2H, amide NH); 8.73 (s; 2H; CH4/5 or CH2/7); 8.21 (s, 2H; CH4/5 or CH2/7); 5.17 (t, *J* = 13.9 Hz; 4H); 2.37 (s, 4H); 1.11 (s, 18H, *t*-Bu).

^{13}C NMR (126 MHz, DMSO-*d$_6$*) δ_{DMSO}: 170.65, 164.29, 135.92, 124.09, 123.71, 120.64, 119.82, 119.33, 114.45 (t, *J* = 30.4 Hz), 59.33 (t, *J* = 26.8 Hz), 49.01, 30.93, 29.62. The remaining aliphatic carbons were barely visible due to coupling with fluorine atoms.

^{19}F NMR (470 MHz, DMSO-*d$_6$*) δ_{DMSO}: -80.45 (t; *J* = 9.0 Hz; 6F); -119.10 (m, 4F); -127.06 (m, 4F)

HR MS (TOF MS ES$^-$) m/z calcd. for $C_{34}H_{33}N_3O_6F_{14}Na^+$: 868.2044 found: 868.2028.

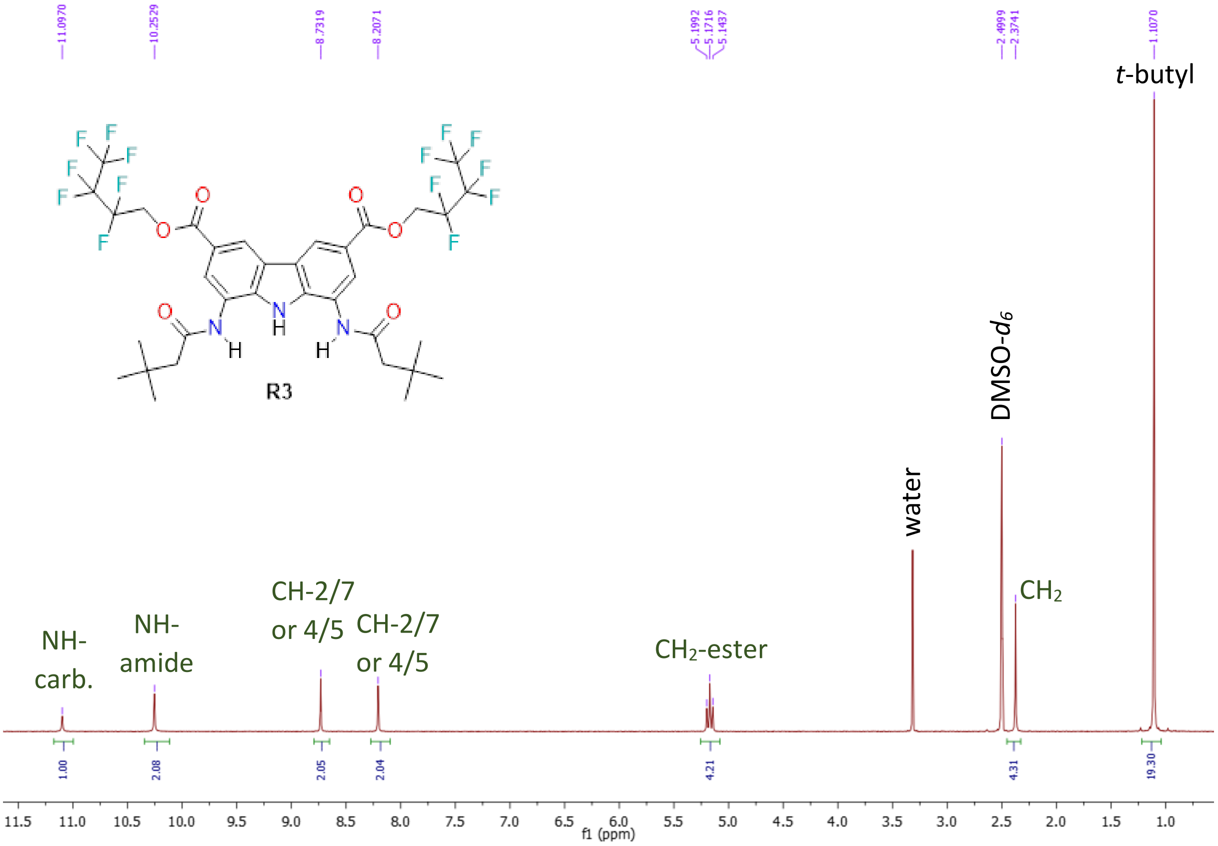

Figure S23. ^{1}H NMR spectrum of **R3** in DMSO-d_6.

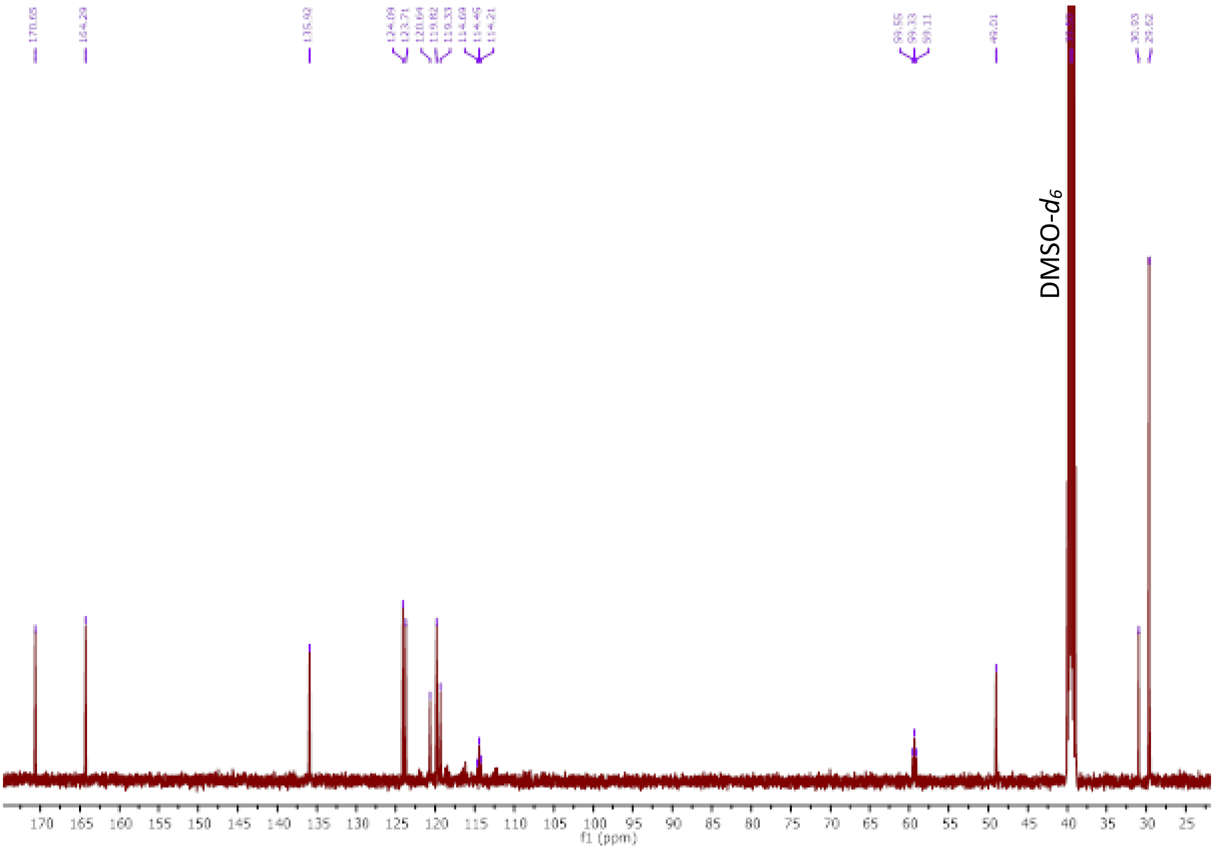

Figure S24. ^{13}C NMR spectrum of **R3** in DMSO-d_6.

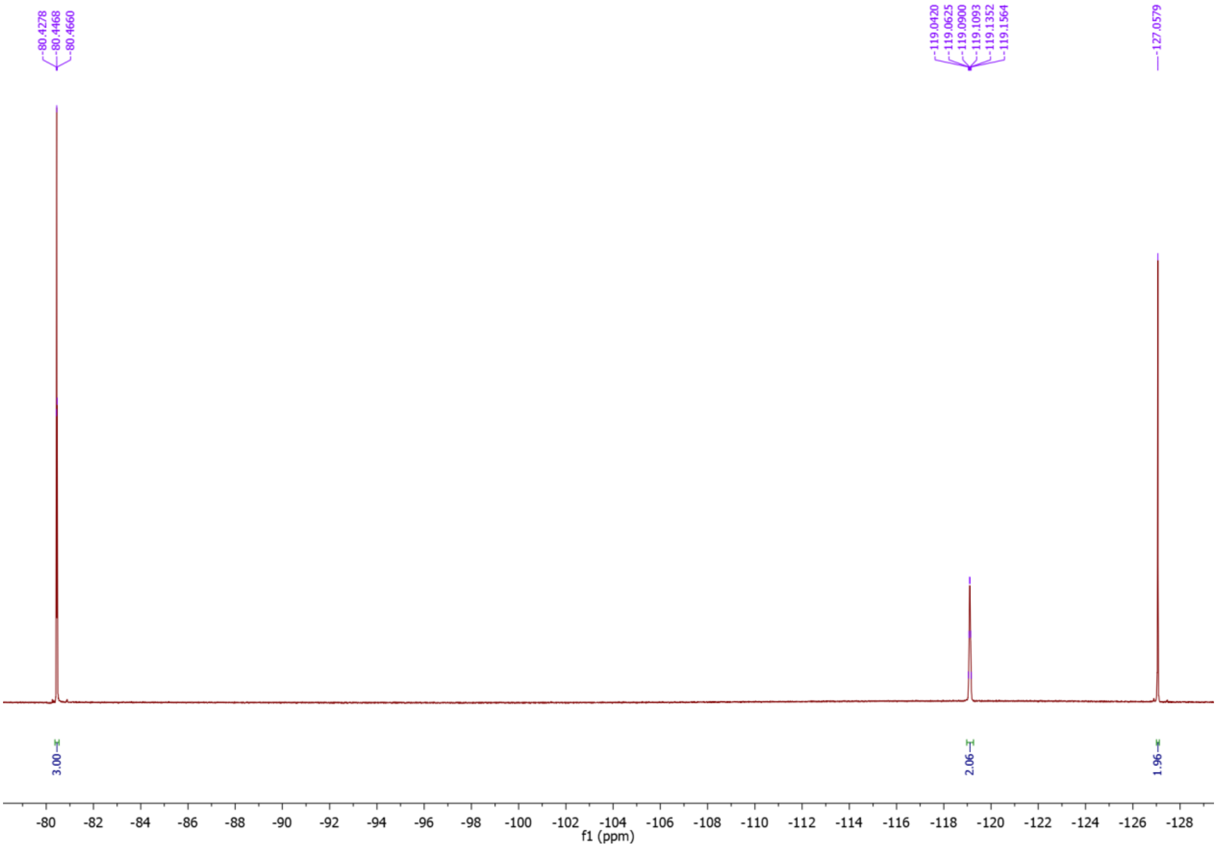

Figure S25. ^{19}F NMR spectrum of **R3** in DMSO-d_6.

3 Binding studies of receptor R1

3.1 General procedure for ^{1}H NMR titrations

Preparation of standard solutions. All reagents were weighted separately on a Mettler Toledo Excellence XA105DU analytical balance (readability 0.01 mg) in screw-capped vials sealed with Teflon-covered septa. DMSO/H_2O mixtures were prepared using Milli-Q water, and their concentrations were expressed as weight-to-weight percentage. All solvent and solution manipulations were performed using Hamilton gas-tight syringes. The titrant was prepared by dissolving TBACl in the receptor solution to avoid receptor dilution during titration. The uncertainty of the concentration of standard solutions was estimated to be ± 2% for receptor, and ± 1% for anion.

^{1}H NMR titration procedure. Titrations were conducted in screw-capped NMR tubes sealed with Teflon-covered septa. Aliquots of the titrant solution were added to the receptor solution, and ^{1}H NMR spectra were recorded after each addition. The NMR spectra were measured using an Agilent 400 MHz spectrometer. More specifically, to a solution of the host (600 µl, 0.01 ± 0.0002 M) in a septum-sealed, screw-capped NMR tube, appropriate aliquots of titrant (0.3 ± 0.003 M TBACl, dissolved in the host solution to avoid dilution) were added using a 25 µl gas-tight microsyringe. Association constants were calculated based on the changes in chemical shifts of the most affected protons of the ligand, as detailed in each specific case below.

3.2 General procedure for UV-vis titrations

Preparation of standard solutions. All reagents were weighted separately using a Mettler Toledo Excellence XA105DU analytical balance (readability 0.01 mg) in screw-capped vials sealed with Teflon-covered septa. DMSO/H_2O mixtures were prepared using Milli-Q water, and their concentrations were expressed as weight-to-weight percentages. All solvent and solution manipulations were conducted using Hamilton gas-tight syringes. Titrants were prepared by dissolving the appropriate TBA salts in the solution of the appropriate receptor (unless specified otherwise) to avoid dilution of the receptor during titration. The uncertainty of the concentration of standard solutions was estimated to be ± 3% for receptors, and ± 2% for anions.

UV-vis titration procedure. To a solution of the host (3 ml, 1×10^{-4} M ± 0.04×10^{-4} M) in a septum-sealed, screw-capped precision cell made of SUPRASIL Quartz (optical path length: 10 mm), appropriate aliquots of the titrant (0.0075 ± 0.000075 M) were added using a 25 µl gas-tight microsyringe. UV-vis spectra were recorded at 25°C using a Thermo Scientific Evolution 300 UV-vis spectrometer.

3.3 Data fitting

The ^{1}H NMR titration data were fitted with BindFit software.[4] Association constant and chemical shifts of 1:1 complexes were set as free parameters for fitting, whereas chemical shifts of the free ligand were constrained to be equal to the experimentally measured values. Association constants derived from independent experiments were averaged using arithmetic mean.

The UV-vis titration data were fitted with HySS software. Association constants were calculated from changes in absorbance at fixed wavelengths. For each titration six wavelengths were chosen in the region of the highest absolute absorbance change, as specified in each case below. Nonlinear curve fitting was carried out using the HypSpec software. Association constants and molar absorption coefficients of complexes were set as free parameters for fitting. Logarithms of association constants were averaged using arithmetic mean from at least two separate experiments.

[4] http://supramolecular.org; a) P. Thordarson, Determining association constants from titration experiments in supramolecular chemistry, *Chem. Soc. Rev.* **2011**, *40*, 1305-1323; b) D. B. Hibbert, P. Thordarson, The death of the Job plot, transparency, open science and online tools, uncertainty estimation methods and other developments in supramolecular chemistry data analysis, *Chem. Commun.* **2016**, *52*, 12792-12805.

3.4 ^{1}H NMR titration of **R1** with TBACl.

^{1}H NMR titration of 0.01 M solution of **R1** in DMSO-d_6/0.5% H_2O with 0.3 M solution of TBA^+Cl^- (dissolved in the solution of **R1**).

Titration of **R1** with TBACl produced the most significant changes in the ^{1}H NMR chemical shifts of the carbazole NH and amide NHs. The data sets for these two signals were therefore simultaneously fitted with the 1:1 model using Bindfit software.

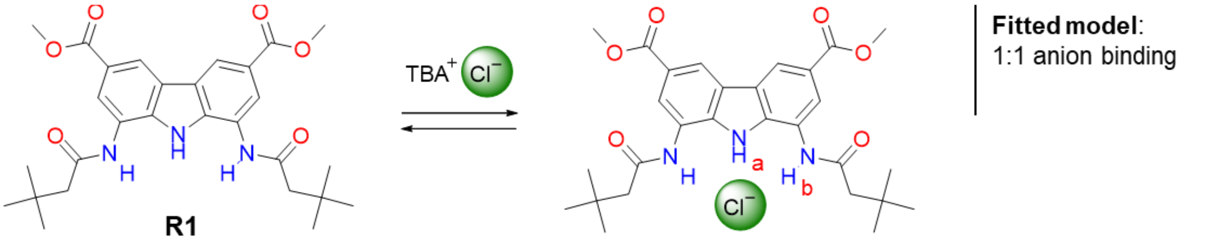

Scheme 1. Chloride binding to **R1**.

Stack of ^{1}H NMR spectra from the titration of R1 with TBACl

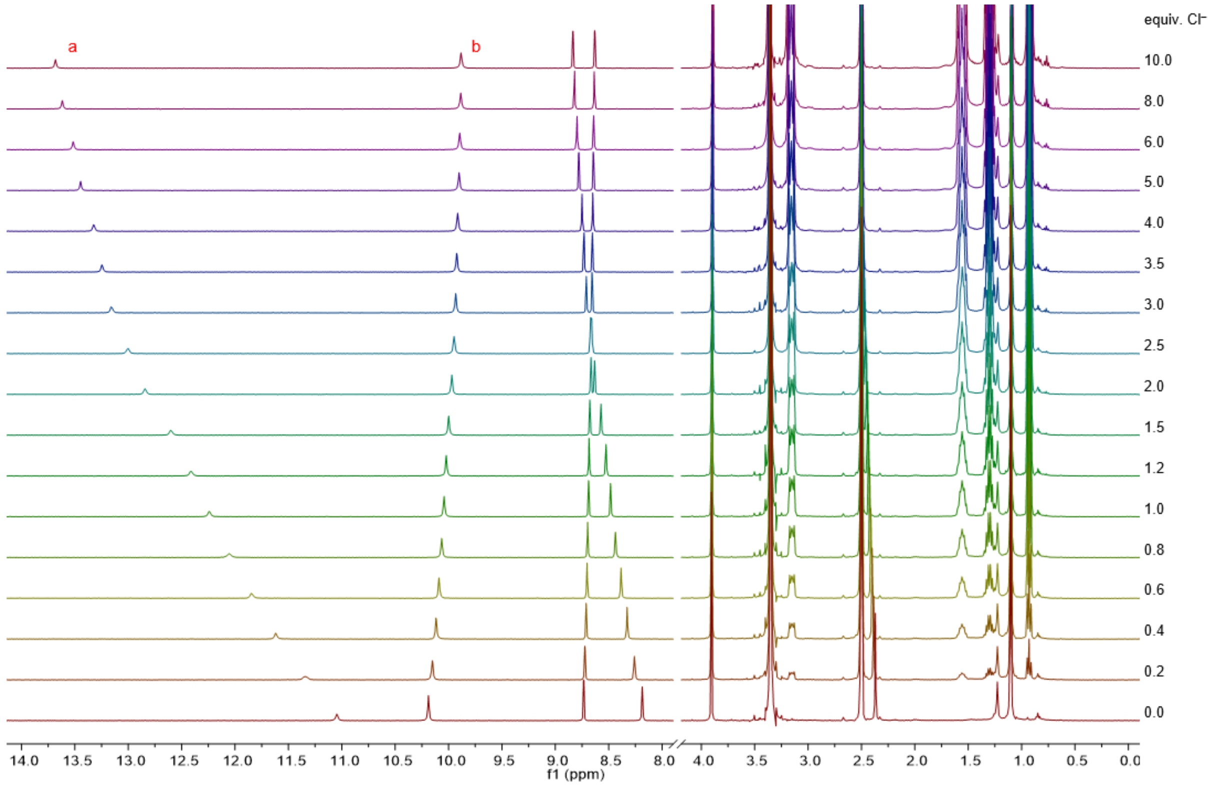

Figure S26. ^{1}H NMR titration of 0.01 M **R1** with TBACl.

Raw data

Added volume of titrant solution [µl]	Equivalents of TBA$^+$Cl$^-$	Chemical Shifts [ppm]	
		NH(amide)	NH(carbazole)
0.0	0.0	10.19080	11.00785
4.0	0.2	10.15305	11.30870
8.0	0.4	10.11970	11.58275
12.0	0.6	10.09095	11.81430
16.5	0.8	10.06470	12.03180
20.5	1.0	10.04315	12.21095
25.0	1.2	10.02250	12.38130
31.5	1.5	10.00050	12.56615
43.0	2.0	9.96885	12.82890
54.5	2.5	9.94825	12.99900
66.5	3.0	9.93220	13.14500
79.0	3.5	9.92175	13.23915
92.5	4.0	9.91315	13.32085
120.0	5.0	9.90145	13.43310
150.0	6.0	9.89365	13.51035
218.0	8.0	9.88510	13.61465
300.0	10.0	9.88155	13.68045

Exemplary ^{1}H NMR titration curves:

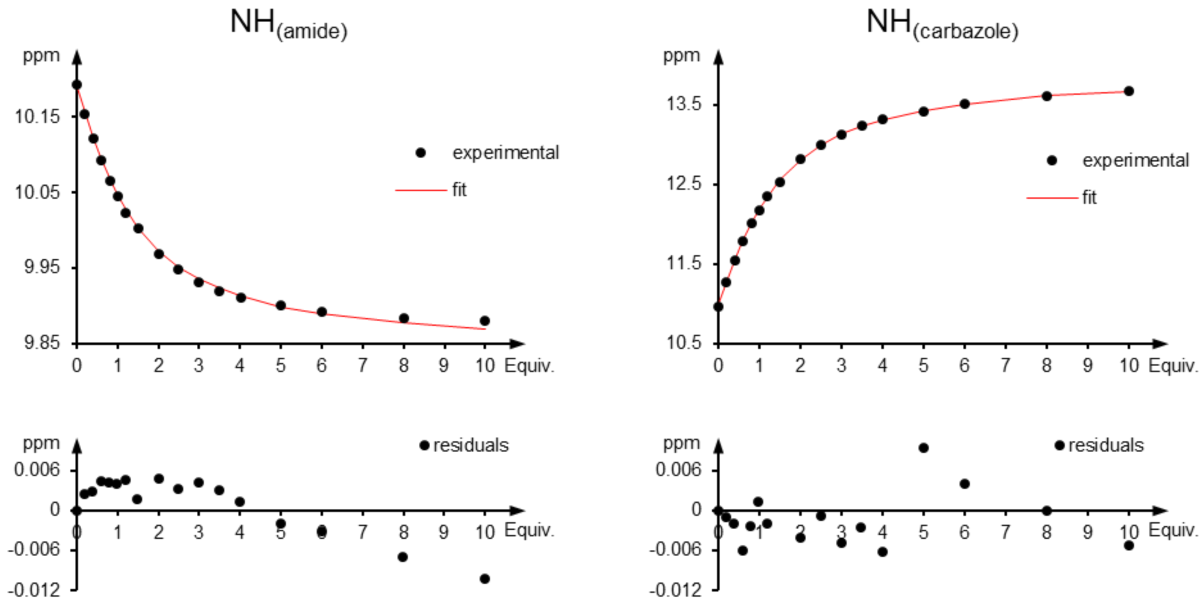

Figure S27. Data points and fitting curves for ^{1}H NMR titration of **R1** (0.01 M in DMSO-d_6/0.5% H_2O) with TBACl.

a) Binding constant K derived from simultaneous fitting of 1:1 model to the two selected protons using BindFit:

K = 119.7 ± 0.7 M^{-1}

b) Binding constant K derived from an independent experiment, repeated according to the same methodology:

K = 124.0 ± 0.8 M^{-1}

c) Chemical shifts derived from simultaneous fitting of 1:1 model to the two selected protons using BindFit:

**^{1}H NMR signal**	**Titration**	**R1** [ppm]	**R1** × $TBA^{+}Cl^{-}$ [ppm]
$NH_{(amide)}$	Titration 1:	10.1932	9.8799
	Titration 2:	10.1884	9.8832
$NH_{(carbazole)}$	Titration 1:	10.9695	13.6793
	Titration 2:	11.0462	13.6816

d) Binding constant averaged from the two experiments:

K = 121.9 M^{-1}

3.5 UV-vis titration of **R1** with TBACl.

UV-vis titration of 10^{-4} M solution of **R1** in DMSO/0.5% H_2O with 0.0075 M solution of TBA^+Cl^- (dissolved in the solution of receptor **R1**).

Stack of UV-vis spectra from the titration of R1 with TBACl in DMSO/0.5% H_2O

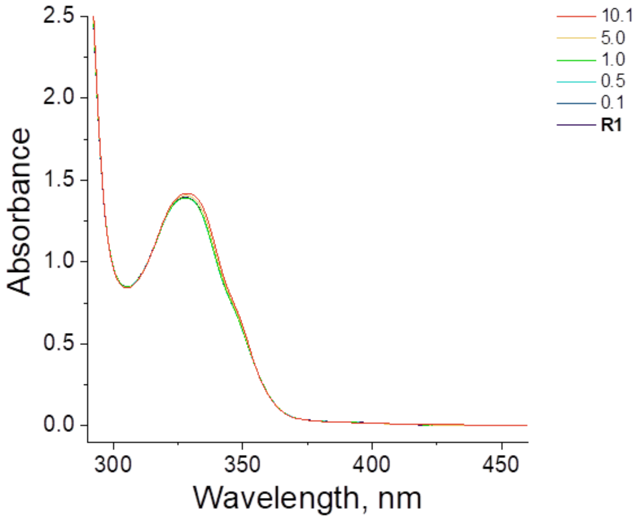

Figure S28. Stack of UV-vis spectra obtained during the titration of 10^{-4} M solution of receptor **R1** in DMSO/0.5% H_2O with 0.0075 M solution of TBACl (dissolved in the solution of receptor **R1**).

UV-vis titration of 10^{-4} M solution of **R1** in DMSO/10% H_2O with 0.075 M solution of TBA^+Cl^- (dissolved in the solution of receptor **R1**).

Stack of UV-vis spectra from the titration of R1 with TBACl in DMSO/10% H_2O

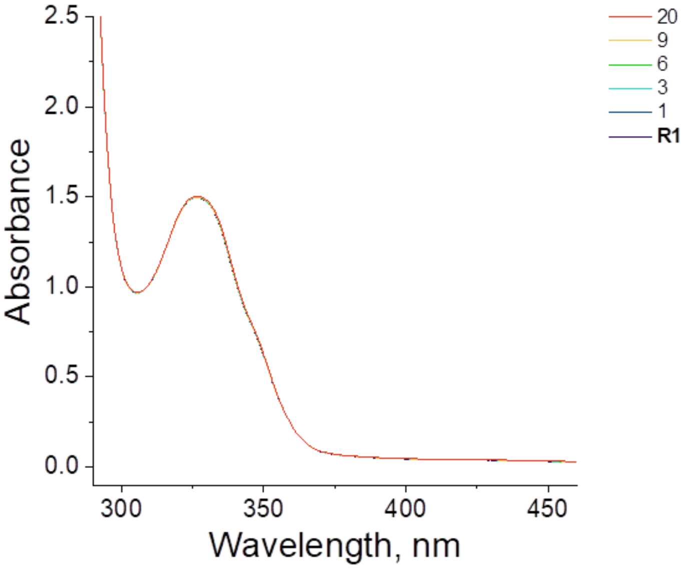

Figure S29. Stack of UV-vis spectra obtained during the titration of 10^{-4} M solution of receptor **R1** in DMSO/10% H_2O with 0.075 M solution of TBACl (dissolved in the solution of receptor **R1**).

3.7 UV-vis titration of **R1** with TBAPhCOO.

UV-vis titration of 10^{-4} M solution of **R1** in DMSO/0.5% H_2O with 0.0075 M solution of TBA^+PhCOO^- (dissolved in the solution of receptor **R1**).

Stack of UV-vis spectra from the titration of R1 with TBAPhCOO in DMSO/0.5% H_2O

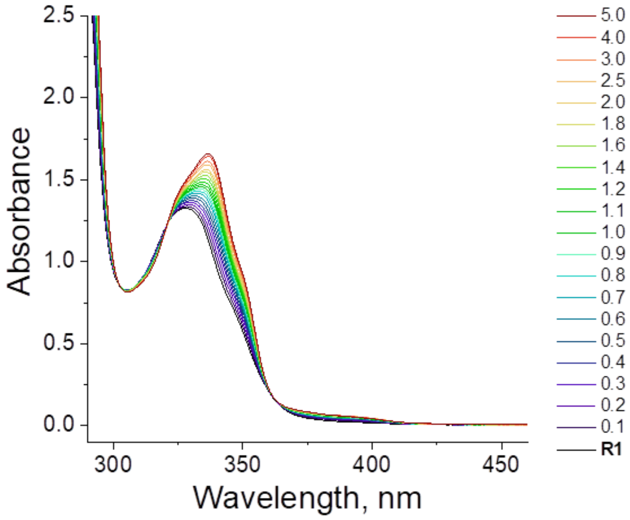

Figure S30. Stack of UV-vis spectra obtained during the titration of 10^{-4} M solution of receptor **R1** in DMSO/0.5% H_2O with 0.0075 M solution of TBAPhCOO (dissolved in the solution of receptor **R1**).

Raw Data

Added volume of titrant solution [µl]	Equivalents of TBAPhCOO	Wavelength [nm]					
		336	337	338	339	340	341
		Absorbance					
0.0	0.0	1.161	1.113	1.061	1.009	0.958	0.909
4.0	0.1	1.197	1.154	1.104	1.056	1.005	0.955
8.0	0.2	1.231	1.191	1.145	1.097	1.047	0.997
12.0	0.3	1.268	1.231	1.188	1.142	1.092	1.040
16.0	0.4	1.296	1.262	1.221	1.176	1.126	1.073
20.0	0.5	1.325	1.295	1.256	1.212	1.163	1.110
24.0	0.6	1.353	1.325	1.288	1.246	1.196	1.142
28.5	0.7	1.383	1.358	1.323	1.282	1.233	1.178
32.5	0.8	1.404	1.381	1.348	1.307	1.259	1.203
36.5	0.9	1.424	1.404	1.372	1.333	1.285	1.229
40.5	1.0	1.442	1.422	1.393	1.355	1.306	1.250
44.5	1.1	1.459	1.442	1.414	1.376	1.328	1.271
49.0	1.2	1.480	1.464	1.438	1.401	1.352	1.295
57.0	1.4	1.505	1.492	1.467	1.431	1.383	1.325
65.5	1.6	1.527	1.516	1.492	1.458	1.410	1.351
74.0	1.8	1.548	1.540	1.518	1.485	1.437	1.378
82.0	2.0	1.562	1.556	1.535	1.502	1.454	1.395
103.5	2.5	1.593	1.589	1.571	1.539	1.493	1.433
125.0	3.0	1.613	1.611	1.594	1.563	1.516	1.455
169.0	4.0	1.641	1.642	1.628	1.598	1.552	1.490
215.5	5.0	1.658	1.659	1.645	1.615	1.568	1.505

Exemplary UV-vis titration curve (absorbance at 338 nm)

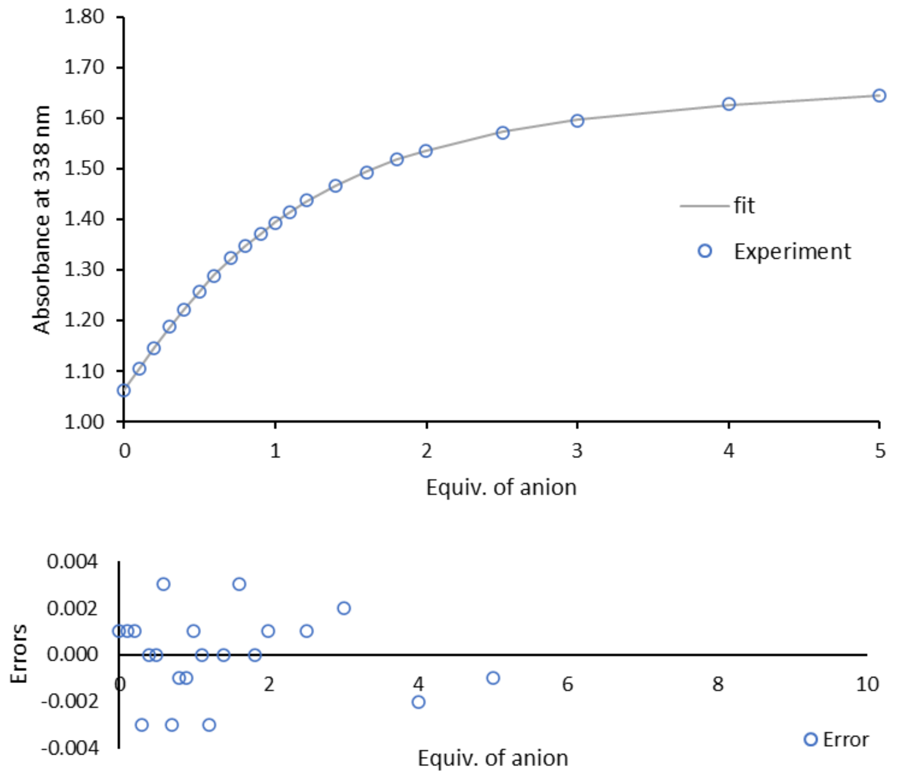

Figure S31. Fitting of 1:1 (receptor:anion) model to the results of the titration of 10^{-4} M solution of receptor **R1** in DMSO/0.5% H_2O with 0.0075 M solution of TBAPhCOO (dissolved in the solution of receptor **R1**).

a) Binding constant K derived from simultaneous fitting of 1:1 model to the six selected wavelengths using HypSpec:

log K = 4.3282 ± 0.0005

b) Binding constant K derived from the experiment repeated according to the same methodology:

log K = 4.3349 ± 0.0003

c) Binding constant averaged from the two experiments:

log K = 4.332

UV-vis titration of 10^{-4} M solution of **R1** in DMSO/10% H_2O with 0.075 M solution of TBA^+PhCOO^- (dissolved in the solution of receptor **R1**).

Stack of UV-vis spectra from the titration of R1 with TBAPhCOO in DMSO/10% H_2O

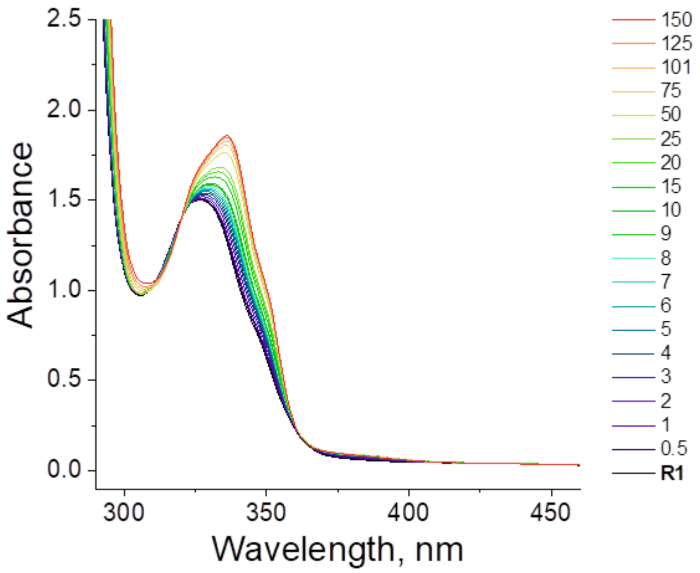

Figure S32. Stack of UV-vis spectra obtained during the titration of 10^{-4} M solution of receptor **R1** in DMSO/10% H_2O with 0.075 M solution of TBAPhCOO (dissolved in the solution of receptor **R1**).

Raw Data

Added volume of titrant solution [µl]	Equivalents of TBAPhCOO	Wavelength [nm]					
		337	338	339	340	341	342
		Absorbance					
0	0.0	1.214	1.155	1.096	1.041	0.985	0.938
2	0.5	1.235	1.178	1.120	1.066	1.010	0.961
4	1.0	1.258	1.201	1.144	1.089	1.034	0.984
8	2.0	1.294	1.241	1.184	1.131	1.073	1.022
12	3.0	1.328	1.276	1.221	1.167	1.109	1.055
16	4.0	1.356	1.306	1.252	1.197	1.139	1.084
20	5.0	1.384	1.335	1.281	1.226	1.167	1.110
24	6.0	1.408	1.360	1.308	1.252	1.193	1.135
28	6.9	1.430	1.383	1.332	1.277	1.215	1.156
32	7.9	1.451	1.405	1.354	1.299	1.237	1.176
37	9	1.475	1.431	1.380	1.324	1.261	1.200
41	10	1.490	1.447	1.396	1.341	1.277	1.215
62	15	1.560	1.521	1.472	1.416	1.350	1.283
82	20	1.607	1.571	1.523	1.466	1.399	1.330
104	25	1.645	1.610	1.564	1.508	1.438	1.366
214	50	1.746	1.717	1.672	1.615	1.542	1.465
334	75	1.792	1.766	1.723	1.664	1.589	1.509
465	101	1.820	1.794	1.752	1.694	1.617	1.536
600	125	1.838	1.814	1.771	1.713	1.635	1.553
750	150	1.850	1.826	1.783	1.725	1.647	1.563

Exemplary UV-vis titration curve (absorbance at 340 nm)

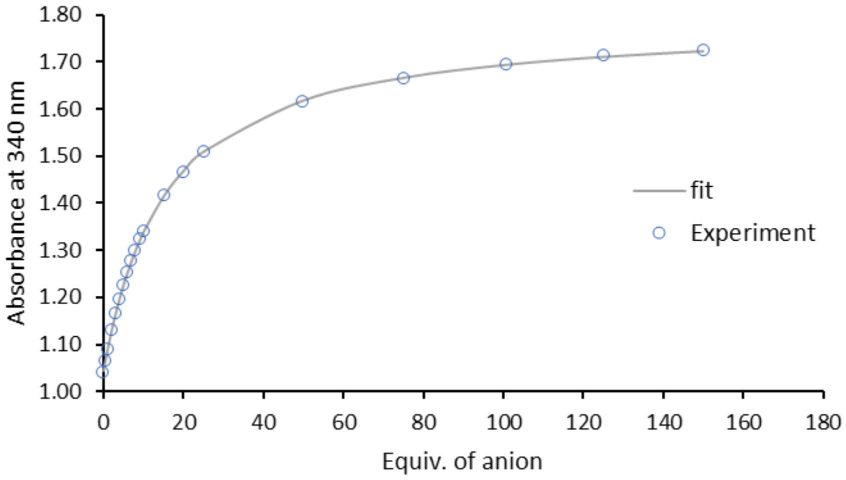

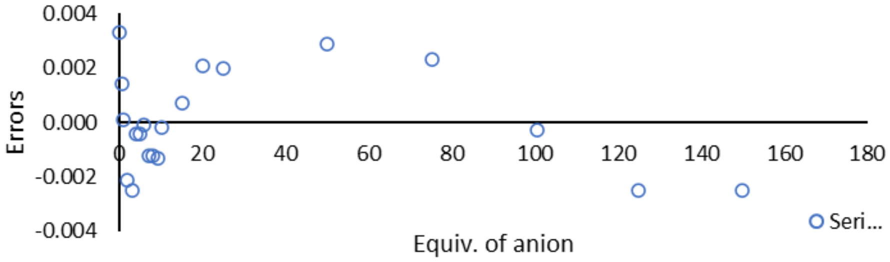

Figure S33. Fitting of 1:1 (receptor:anion) model to the results of the titration of 10^{-4} M solution of receptor **R1** in DMSO/10% H_2O with 0.075 M solution of TBAPhCOO (dissolved in the solution of receptor **R1**).

a) Binding constant K derived from simultaneous fitting of 1:1 model to the six selected wavelengths using HypSpec:

log K = 2.8328 ± 0.0001

b) Binding constant K derived from the experiment repeated according to the same methodology:

log K = 2.7902 ± 0.0002

c) Binding constant averaged from the two experiments:

log K = 2.812

3.8 UV-vis titration of **R1** with $TBACH_3COO$.

UV-vis titration of 10^{-4} M solution of **R1** in DMSO/0.5% H_2O with 0.0075 M solution of $TBA^+CH_3COO^-$ (dissolved in the solution of receptor **R1**).

Stack of UV-vis spectra from the titration of R1 with $TBACH_3COO$ in DMSO/0.5% H_2O

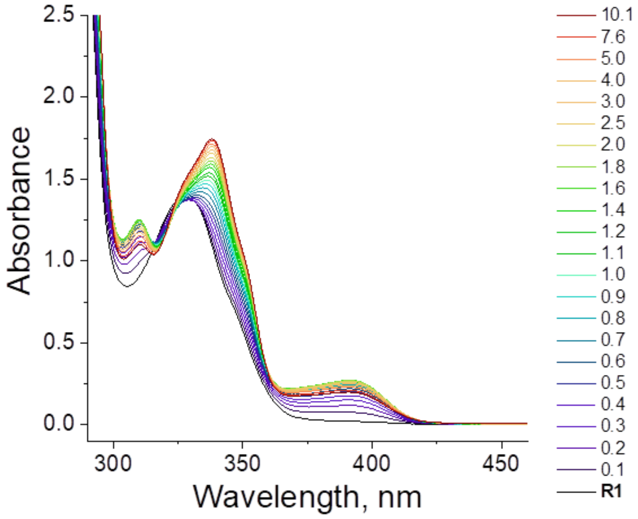

Figure S34. Stack of UV-vis spectra obtained during the titration of 10^{-4} M solution of receptor **R1** in DMSO/0.5% H_2O with 0.0075 M solution of $TBACH_3COO$ (dissolved in the solution of receptor **R1**).

Raw Data

Added volume of titrant solution [µl]	Equivalents of TBACH$_3$COO	Wavelength [nm]					
		340	341	342	343	350	351
		Absorbance					
0.0	0.0	1.219	1.219	1.108	1.052	0.995	0.941
4.0	0.1	1.25	1.250	1.152	1.101	1.047	0.995
8.0	0.2	1.286	1.286	1.201	1.154	1.104	1.053
12.0	0.3	1.324	1.324	1.252	1.210	1.162	1.112
16.0	0.4	1.359	1.359	1.298	1.261	1.216	1.167
20.0	0.5	1.396	1.396	1.346	1.312	1.270	1.222
24.0	0.6	1.432	1.432	1.391	1.361	1.321	1.273
28.5	0.7	1.467	1.467	1.436	1.409	1.372	1.325
32.5	0.8	1.498	1.498	1.475	1.451	1.415	1.369
36.5	0.9	1.517	1.517	1.501	1.479	1.445	1.399
40.5	1.0	1.538	1.538	1.527	1.508	1.474	1.429
44.5	1.1	1.558	1.558	1.552	1.533	1.501	1.456
49.0	1.2	1.578	1.578	1.576	1.559	1.528	1.483
57.0	1.4	1.61	1.610	1.616	1.601	1.571	1.527
65.5	1.6	1.633	1.633	1.643	1.630	1.602	1.556
74.0	1.8	1.651	1.651	1.664	1.651	1.625	1.580
82.0	2.0	1.663	1.663	1.678	1.668	1.640	1.596
103.5	2.5	1.687	1.687	1.705	1.695	1.669	1.625
125.0	3.0	1.699	1.699	1.721	1.711	1.684	1.640
169.0	4.0	1.719	1.719	1.742	1.734	1.708	1.663
215.5	5.0	1.73	1.730	1.755	1.748	1.721	1.677
340.5	7.6	1.219	1.219	1.108	1.052	0.995	0.941
465.5	10.1	1.25	1.250	1.152	1.101	1.047	0.995

Exemplary UV-vis titration curve (absorbance at 340 nm)

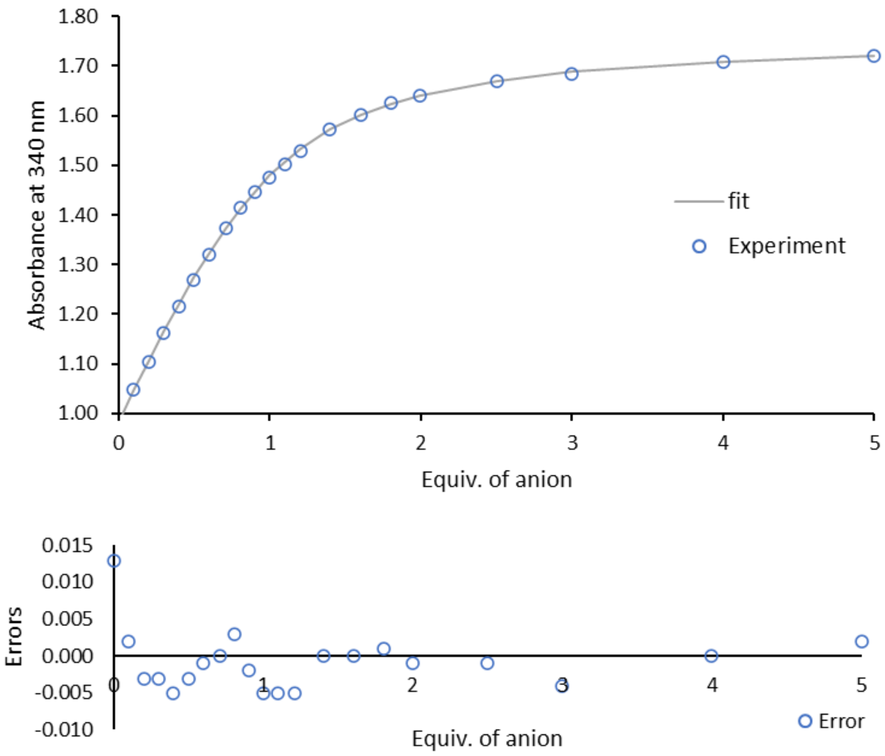

Figure S35. Fitting of 1:1 (receptor:anion) model to the results of the titration of 10^{-4} M solution of receptor **R1** in DMSO/0.5% H_2O with 0.0075 M solution of $TBACH_3COO$ (dissolved in the solution of receptor **R1**).

a) Binding constant K derived from simultaneous fitting of 1:1 model to six selected wavelengths using HypSpec:

log K = 4.6014 ± 0.0012

b) Binding constant K derived from the experiment repeated according to the same methodology:

log K = 4.7021 ± 0.0007

c) Binding constant averaged from the two experiments:

log K = 4.652

UV-vis titration of 10^{-4} M solution of **R1** in DMSO/10% H_2O with 0.075 M solution of $TBA^+CH_3COO^-$ (dissolved in the solution of receptor **R1**).

Stack of UV-vis spectra from the titration of R1 with $TBACH_3COO$ in DMSO/10% H_2O

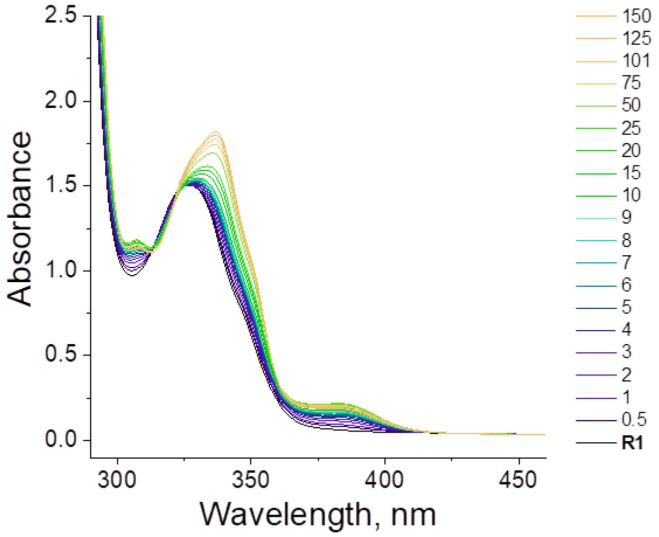

Figure S36. Stack of UV-vis spectra obtained during the titration of 10^{-4} M solution of receptor **R1** in DMSO/10% H_2O with 0.075 M solution of $TBACH_3COO$ (dissolved in the solution of receptor **R1**).

Raw Data

Added volume of titrant solution [µl]	Equivalents of $TBACH_3COO$	Wavelength [nm]					
		338	339	340	341	342	343
		Absorbance					
0	0.0	1.155	1.098	1.042	0.987	0.938	0.895
2	0.5	1.185	1.129	1.075	1.020	0.972	0.928
4	1.0	1.205	1.150	1.098	1.043	0.995	0.951
8	2.0	1.238	1.186	1.134	1.081	1.031	0.986
12	3.0	1.267	1.217	1.166	1.112	1.062	1.015
16	4.0	1.292	1.243	1.193	1.139	1.089	1.040
20	5.0	1.316	1.268	1.218	1.164	1.113	1.063
24	6.0	1.337	1.290	1.241	1.187	1.135	1.084
28	6.9	1.356	1.310	1.262	1.207	1.155	1.102
32	7.9	1.374	1.329	1.281	1.226	1.173	1.120
37	9.1	1.394	1.351	1.303	1.248	1.194	1.140
41	10.1	1.408	1.366	1.318	1.264	1.209	1.155
62	15.2	1.473	1.434	1.387	1.332	1.276	1.217
82	20.0	1.520	1.483	1.438	1.381	1.324	1.262
104	25.1	1.559	1.524	1.479	1.422	1.363	1.299
214	49.9	1.668	1.637	1.594	1.536	1.472	1.401
334	75.1	1.726	1.698	1.655	1.595	1.528	1.454
465	100.6	1.759	1.732	1.690	1.629	1.561	1.484
600	125.0	1.787	1.760	1.718	1.656	1.586	1.509
750	150.0	1.805	1.778	1.735	1.673	1.603	1.524

Exemplary UV-vis titration curve (absorbance at 340 nm)

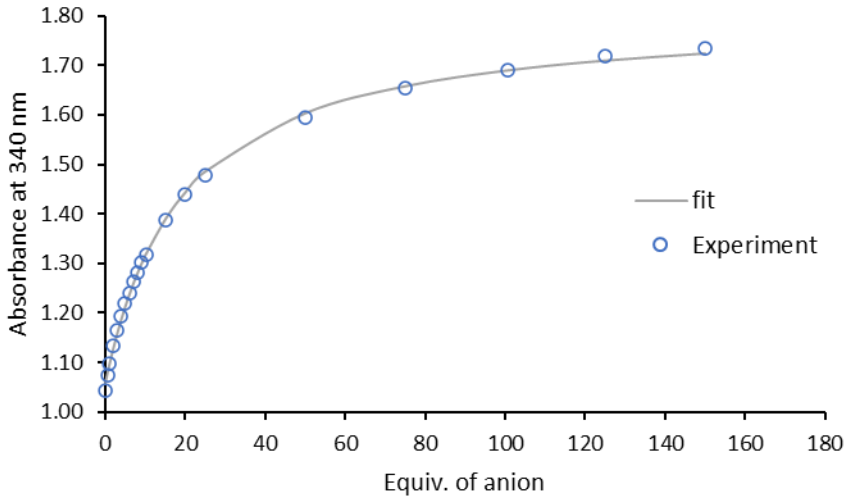

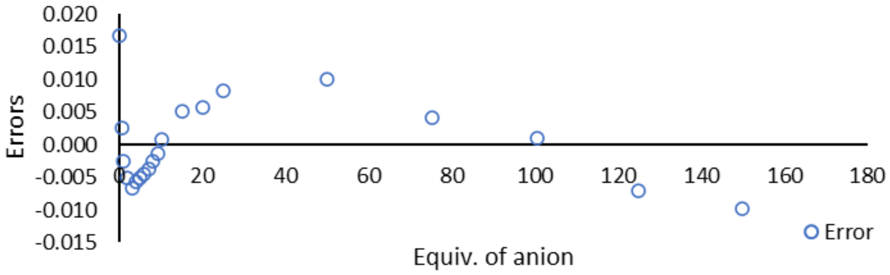

Figure S37. Fitting of 1:1 (receptor:anion) model to the results of the titration of 10^{-4} M solution of receptor **R1** in DMSO/10% H_2O with 0.075 M solution of $TBACH_3COO$ (dissolved in the solution of receptor **R1**).

a) Binding constant K derived from simultaneous fitting of 1:1 model to six selected wavelengths using HypSpec:

log K = 2.7366 ± 0.0005

b) Binding constant K derived from the experiment repeated according to the same methodology:

log K = 2.6776 ± 0.0004

c) Binding constant averaged from the two experiments:

log K = 2.707

3.9 UV-vis titration of **R1** with $TBAH_2PO_4$.

UV-vis titration of 10^{-4} M solution of **R1** in DMSO/0.5% H_2O with 0.0075 M solution of $TBA^+H_2PO_4^-$ (dissolved in the solution of receptor **R1**).

Stack of UV-vis spectra from the titration of R1 with $TBAH_2PO_4$ in DMSO/0.5% H_2O

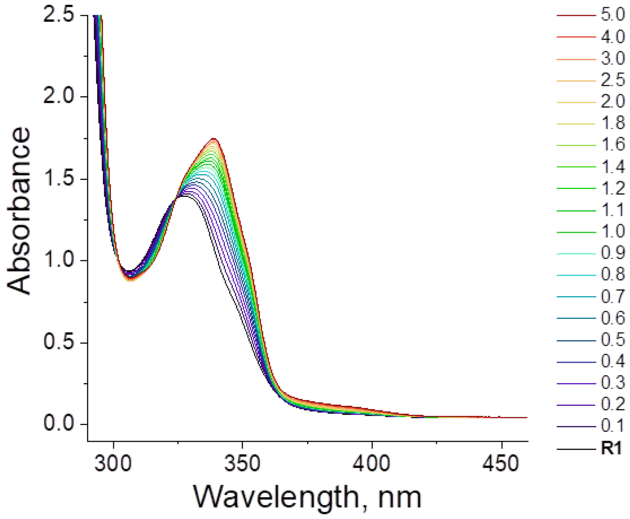

Figure S38. Stack of UV-vis spectra obtained during the titration of 10^{-4} M solution of receptor **R1** in DMSO/0.5% H_2O with 0.0075 M solution of $TBAH_2PO_4$ (dissolved in the solution of receptor **R1**).

Raw Data

Added volume of titrant solution [µl]	Equivalents of $TBAH_2PO_4$	Wavelength [nm]					
		338	339	340	341	342	350
		Absorbance					
0.0	0.0	1.111	1.057	1.000	0.947	0.898	0.577
4.0	0.1	1.170	1.120	1.067	1.016	0.967	0.625
8.0	0.2	1.234	1.190	1.140	1.091	1.043	0.677
12.0	0.3	1.298	1.258	1.213	1.166	1.118	0.729
16.0	0.4	1.355	1.319	1.278	1.233	1.185	0.775
20.0	0.5	1.408	1.378	1.340	1.297	1.249	0.820
24.0	0.6	1.459	1.433	1.398	1.356	1.309	0.862
28.5	0.7	1.513	1.490	1.459	1.419	1.372	0.906
32.5	0.8	1.556	1.536	1.507	1.469	1.422	0.941
36.5	0.9	1.591	1.575	1.548	1.512	1.465	0.971
40.5	1.0	1.623	1.609	1.584	1.548	1.503	0.996
44.5	1.1	1.647	1.636	1.613	1.578	1.532	1.018
49.0	1.2	1.674	1.664	1.644	1.608	1.564	1.039
57.0	1.4	1.707	1.700	1.680	1.648	1.603	1.068
65.5	1.6	1.728	1.723	1.706	1.674	1.629	1.088
74.0	1.8	1.745	1.741	1.724	1.693	1.648	1.101
82.0	2.0	1.759	1.756	1.739	1.709	1.665	1.114
103.5	2.5	1.779	1.779	1.765	1.735	1.691	1.133
125.0	3.0	1.790	1.790	1.777	1.749	1.705	1.145
169.0	4.0	1.802	1.803	1.792	1.763	1.720	1.159
215.5	5.0	1.809	1.812	1.801	1.773	1.730	1.166
340.5	7.6	1.816	1.818	1.809	1.781	1.739	1.178
465.5	10.1	1.820	1.823	1.813	1.787	1.746	1.184

Exemplary UV-vis titration curve (absorbance at 340 nm)

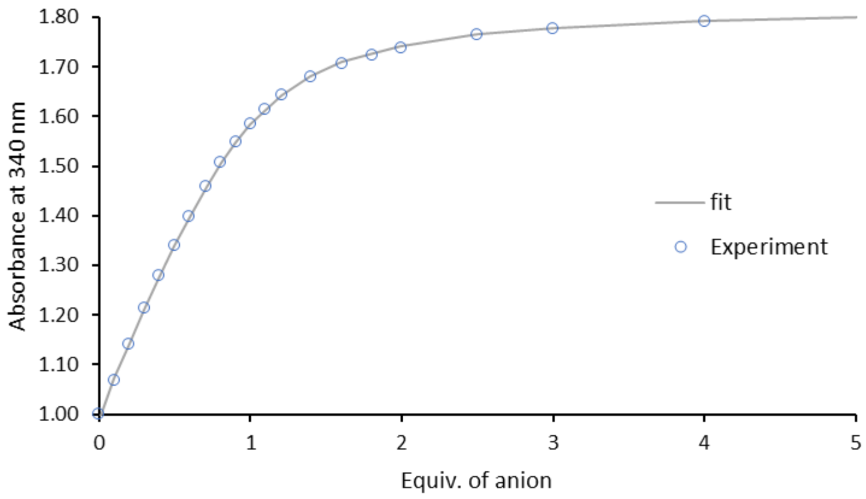

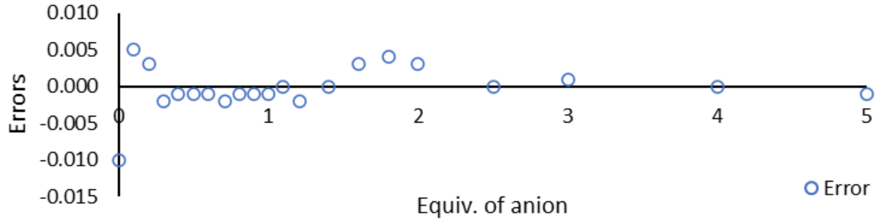

Figure S39. Fitting of 1:1 (receptor:anion) model to the results of the titration of 10^{-4} M solution of receptor **R1** in DMSO/0.5% H_2O with 0.0075 M solution of $TBAH_2PO_4$ (dissolved in the solution of receptor **R1**).

a) Binding constant K derived from simultaneous fitting of 1:1 model to the six selected wavelengths using HypSpec:

log K = 4.9617 ± 0.0015

b) Binding constant K derived from the experiment repeated according to the same methodology:

log K = 4.9211 ± 0.0002

c) Binding constant averaged from the two experiments:

log K = 4.941

UV-vis titration of 10^{-4} M solution of **R1** in DMSO/0.5% H_2O with 0.075 M solution of $TBA^{+}H_2PO_4^{-}$ (dissolved in the solution of receptor **R1**).

Stack of UV-vis spectra from the titration of R1 with $TBAH_2PO_4$ in DMSO/10% H_2O

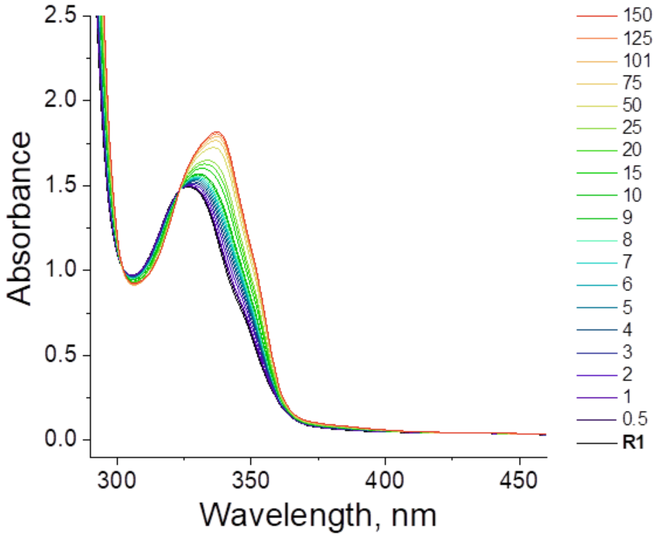

Figure S40. Stack of UV-vis spectra obtained during the titration of 10^{-4} M solution of receptor **R1** in DMSO/10% H_2O with 0.075 M solution of $TBAH_2PO_4$ (dissolved in the solution of receptor **R1**).

Raw Data

Added volume of titrant solution [µl]	Equivalents of $TBAH_2PO_4$	Wavelength [nm]					
		340	341	342	355	356	357
		Absorbance					
0	0.0	1.033	0.978	0.931	0.387	0.349	0.313
2	0.5	1.060	1.006	0.958	0.399	0.359	0.321
4	1.0	1.083	1.029	0.981	0.411	0.370	0.330
8	2.0	1.128	1.075	1.027	0.433	0.388	0.344
12	3.0	1.168	1.116	1.067	0.451	0.404	0.357
16	4.0	1.204	1.152	1.103	0.468	0.418	0.369
20	5.0	1.237	1.185	1.135	0.484	0.432	0.379
24	6.0	1.265	1.214	1.164	0.496	0.442	0.387
28	6.9	1.292	1.241	1.190	0.509	0.453	0.396
32	7.9	1.315	1.264	1.214	0.519	0.461	0.403
37	9.1	1.342	1.291	1.241	0.532	0.472	0.411
41	10.1	1.361	1.311	1.260	0.541	0.479	0.417
62	15.2	1.443	1.395	1.343	0.579	0.511	0.442
82	20.0	1.498	1.450	1.398	0.604	0.532	0.460
104	25.1	1.543	1.495	1.442	0.625	0.550	0.474
214	49.9	1.659	1.614	1.560	0.681	0.597	0.512
334	75.1	1.713	1.669	1.615	0.708	0.621	0.532
465	100.6	1.742	1.698	1.644	0.723	0.633	0.542
600	125.0	1.761	1.719	1.665	0.733	0.643	0.549
750	150.0	1.776	1.734	1.680	0.743	0.651	0.557

Exemplary UV-vis titration curve (absorbance at 340 nm)

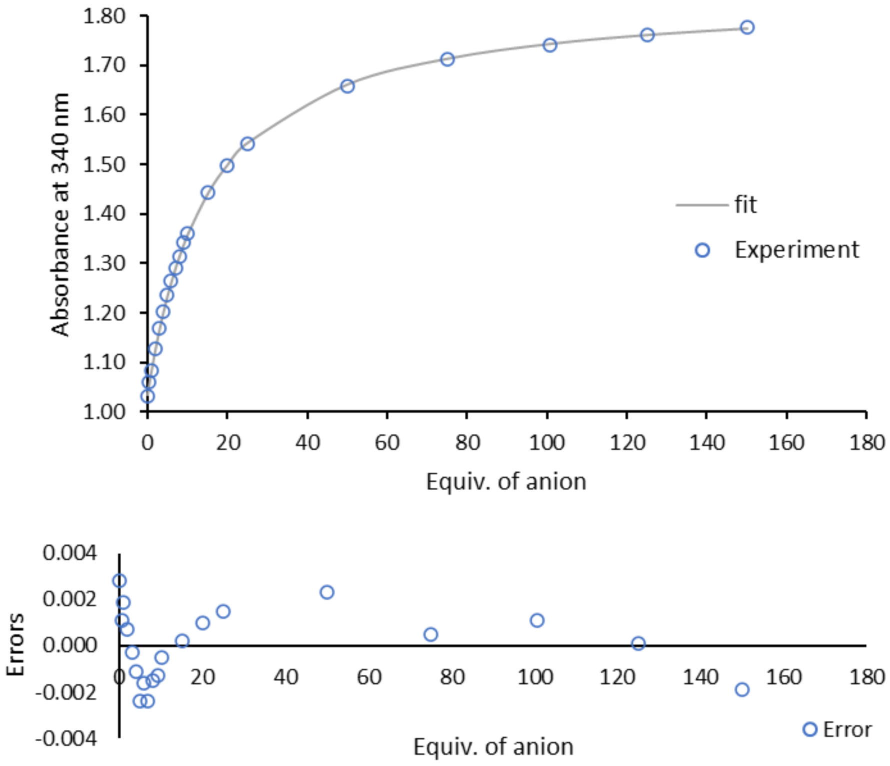

Figure S41. Fitting of 1:1 (receptor:anion) model to the results of the titration of 10^{-4} M solution of receptor **R1** in DMSO/10% H_2O with 0.075 M solution of $TBAH_2PO_4$ (dissolved in the solution of receptor **R1**).

a) Binding constant K derived from simultaneous fitting of 1:1 model to the six selected wavelengths using HypSpec:

log K = 2.8381 ± 0.0002

b) Binding constant K derived from the experiment repeated according to the same methodology:

log K = 2.8207 ± 0.0005

c) Binding constant averaged from the two experiments:

log K = 2.829

3.10 UV-vis titration of **R1** with $(TBA)_2SO_4$.

UV-vis titration of 10^{-4} M solution of **R1** in DMSO/0.5% H_2O with 0.0075 M solution of $(TBA^+)_2SO_4^{2-}$ (dissolved in the solution of receptor **R1**).

Stack of UV-vis spectra from the titration of R1 with TBA_2SO_4 in DMSO/0.5% H_2O

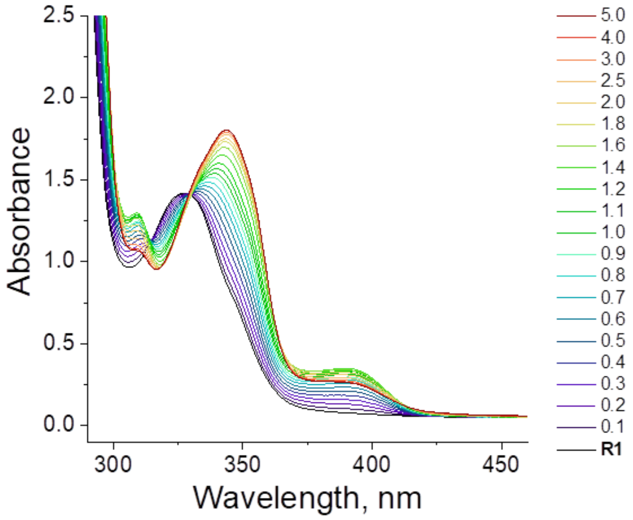

Figure S42. Stack of UV-vis spectra obtained during the titration of 10^{-4} M solution of receptor **R1** in DMSO/0.5% H_2O with 0.0075 M solution of $(TBA)_2SO_4$ (dissolved in the solution of receptor **R1**).

Raw Data

Added volume of titrant solution [µl]	Equivalents of $(TBA)_2SO_4$	Wavelength [nm]					
		346	347	338	349	556	357
		Absorbance					
0.0	0.0	0.788	0.754	0.717	0.677	0.390	0.353
4.0	0.1	0.831	0.795	0.757	0.717	0.420	0.380
8.0	0.2	0.892	0.855	0.816	0.775	0.464	0.421
12.0	0.3	0.955	0.917	0.877	0.836	0.511	0.463
16.0	0.4	1.027	0.988	0.947	0.905	0.565	0.512
20.0	0.5	1.096	1.057	1.016	0.973	0.619	0.561
24.0	0.6	1.166	1.127	1.085	1.042	0.678	0.615
28.5	0.7	1.235	1.197	1.156	1.113	0.739	0.672
32.5	0.8	1.294	1.256	1.215	1.173	0.795	0.722
36.5	0.9	1.357	1.320	1.280	1.237	0.850	0.775
40.5	1.0	1.410	1.375	1.336	1.294	0.906	0.829
44.5	1.1	1.462	1.428	1.390	1.349	0.956	0.877
49.0	1.2	1.518	1.485	1.448	1.408	1.014	0.930
57.0	1.4	1.598	1.569	1.535	1.496	1.102	1.015
65.5	1.6	1.661	1.634	1.601	1.563	1.165	1.075
74.0	1.8	1.704	1.678	1.645	1.607	1.205	1.112
82.0	2.0	1.724	1.700	1.668	1.628	1.223	1.128
103.5	2.5	1.753	1.730	1.697	1.658	1.246	1.149
125.0	3.0	1.768	1.742	1.709	1.670	1.253	1.155
169.0	4.0	1.777	1.752	1.719	1.679	1.259	1.160
215.5	5.0	1.781	1.756	1.723	1.684	1.262	1.162

Exemplary UV-vis titration curves at 349 nm and 385 nm

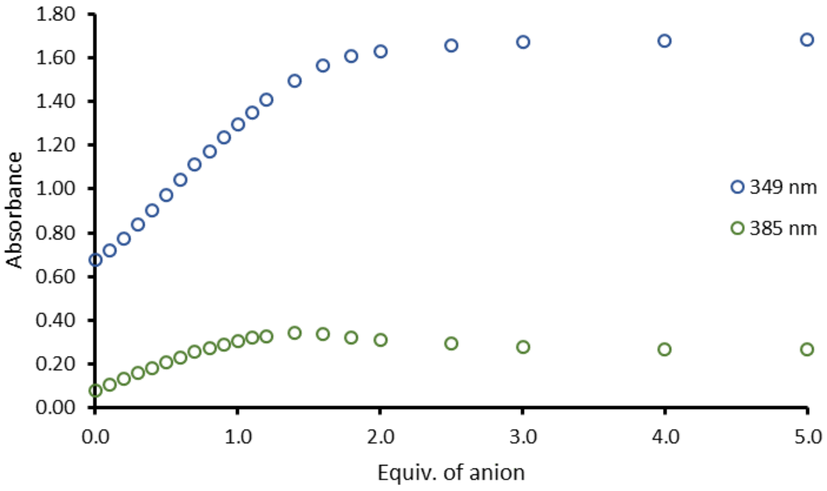

Figure S43. Binding isotherms from the titration of 10^{-4} M solution of receptor **R1** in DMSO/0.5% H_2O with 0.0075 M solution of $(TBA)_2SO_4$ (dissolved in the solution of receptor **R1**), for two wavelengths 349 nm and 385 nm.

UV-vis titration of 10^{-4} M solution of **R1** in DMSO/10% H_2O with 0.075 M solution of $(TBA^+)_2SO_4^{2-}$ (dissolved in the solution of receptor **R1**).

Stack of UV-vis spectra from the titration of R1 with TBA_2SO_4 in DMSO/10% H_2O

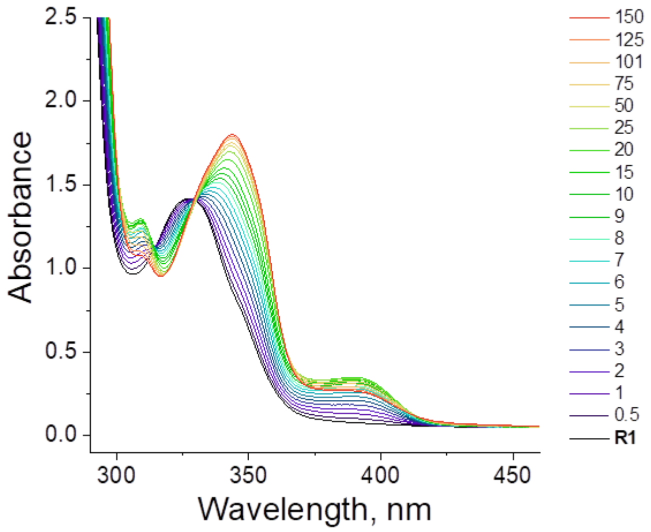

Figure S44. Stack of UV-vis spectra obtained during the titration of 10^{-4} M solution of receptor **R1** in DMSO/10% H_2O with 0.075 M solution of $(TBA)_2SO_4$ (dissolved in the solution of receptor **R1**).

Raw Data

Added volume of titrant solution [μl]	Equivalents of $(TBA)_2SO_4$	Wavelength [nm]					
		338	339	340	341	349	385
		Absorbance					
0	0.0	1.117	1.060	1.006	0.953	0.621	0.059
2	0.5	1.131	1.077	1.024	0.971	0.758	0.092
4	1.0	1.148	1.094	1.042	0.989	0.893	0.109
8	2.0	1.176	1.124	1.072	1.019	1.098	0.129
12	3.0	1.202	1.151	1.101	1.048	1.213	0.137
16	4.0	1.229	1.179	1.129	1.076	1.278	0.140
20	5.0	1.249	1.201	1.151	1.098	1.320	0.143
24	6.0	1.270	1.222	1.173	1.119	1.347	0.147
28	6.9	1.289	1.243	1.194	1.140	1.371	0.146
32	7.9	1.308	1.263	1.214	1.160	1.391	0.145
37	9.1	1.329	1.285	1.236	1.182	1.407	0.144
41	10.1	1.344	1.301	1.253	1.198	1.418	0.144
62	15.2	1.408	1.368	1.321	1.266	1.450	0.142
82	20.0	1.455	1.417	1.371	1.316	1.468	0.140
104	25.1	1.495	1.458	1.413	1.356	1.480	0.137
214	49.9	1.605	1.573	1.529	1.471	1.508	0.131
334	75.1	1.662	1.633	1.590	1.531	1.522	0.127
465	100.6	1.701	1.673	1.630	1.570	1.535	0.125
600	125.0	1.727	1.699	1.657	1.596	1.545	0.124
750	150.0	1.754	1.726	1.683	1.621	1.553	0.124

The absorbance intensity from the UV-vis titration curves at 349 nm and 385 nm

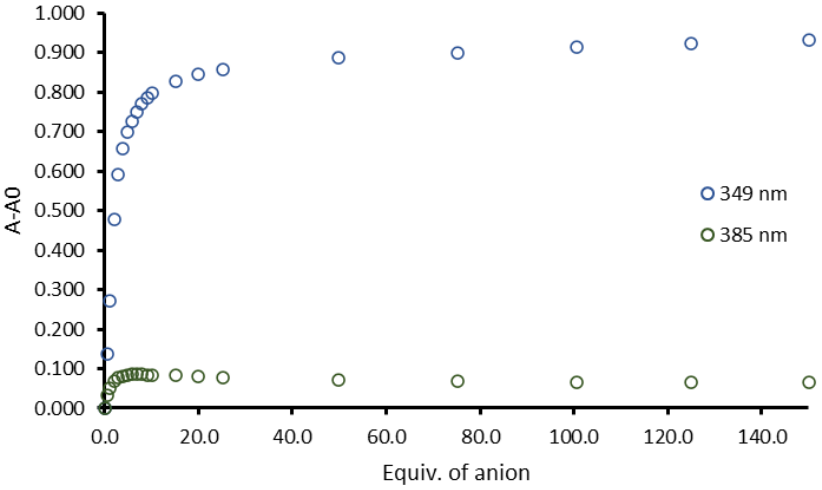

Figure S45. The absorbance intensity from the titration of 10^{-4} M solution of receptor **R1** in DMSO/10% H_2O with 0.075 M solution of $(TBA)_2SO_4$ (dissolved in the solution of receptor **R1**), for two wavelengths 349 nm and 385 nm.

3.11 UV-vis titration of **R1** with TBAOH.

UV-vis titration of 10^{-4} M solution of **R1** in DMSO/0.5% H_2O with 0.0075 M solution of TBA^+OH^- (dissolved in the solution of **R1**).

Stack of UV-vis spectra from the titration of R1 with TBAOH in DMSO/0.5% H_2O

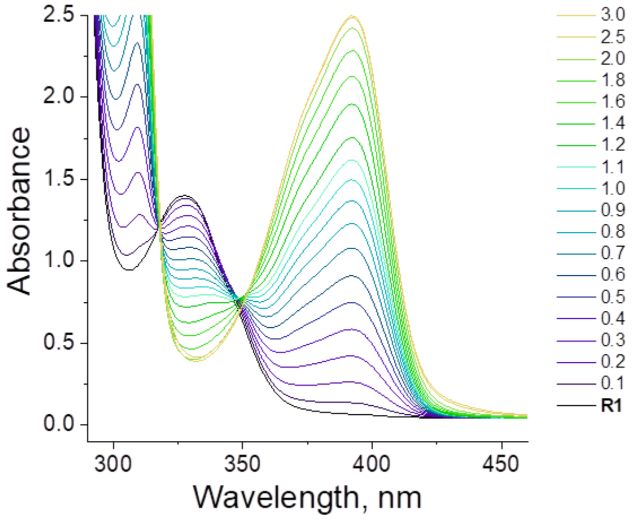

Figure S46. Stack of UV-vis spectra obtained during the titration of 10^{-4} M solution of receptor **R1** in DMSO/0.5% H_2O with 0.0075 M solution of TBAOH (dissolved in the solution of receptor **R1**).

UV-vis titration of 10^{-4} M solution of **R1** in DMSO/10% H_2O with 0.075 M solution of TBA^+OH^- (dissolved in the solution of **R1**).

Stack of UV-vis spectra from the titration of R1 with TBAOH in DMSO/10% H_2O

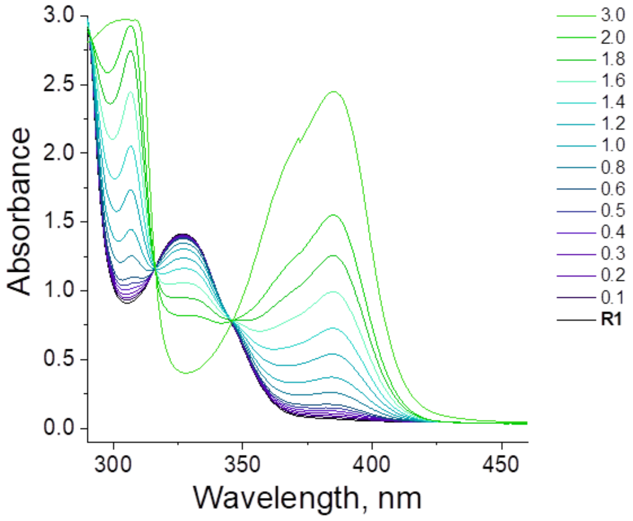

Figure S47. Stack of UV-vis spectra obtained during the titration of 10^{-4} M solution of receptor **R1** in DMSO/10% H_2O with 0.075 M solution of TBAOH (dissolved in the solution of receptor **R1**).

3.12 UV-vis titration of **R1** with $TBAHSO_4$.

UV-vis titration of 10^{-4} M solution of **R1** in DMSO/0.5% H_2O with 0.0075 M solution of $TBA^+HSO_4^-$ (dissolved in the solution of receptor **R1**).

Stack of UV-vis spectra from the titration of R1 with $TBAHSO_4$ in DMSO/0.5%H_2O

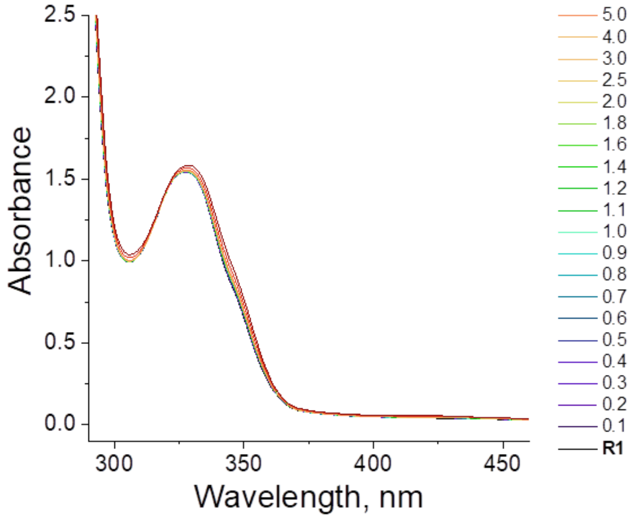

Figure S48. Stack of UV-vis spectra obtained during the titration of 10^{-4} M solution of receptor **R1** in DMSO/0.5% H_2O with 0.0075 M solution of $TBAHSO_4$ (dissolved in the solution of receptor **R1**).

3.13 UV-vis titration of **R2** with $TBAH_2PO_4$.

UV-vis titration of 10^{-4} M solution of **R2** in DMSO/0.5% H_2O with 0.0075 M solution of $TBA^+H_2PO_4^-$ (dissolved in the solution of receptor **R2**).

Stack of UV-vis spectra from the titration of R2 with $TBAH_2PO_4$ in DMSO/0.5%H_2O

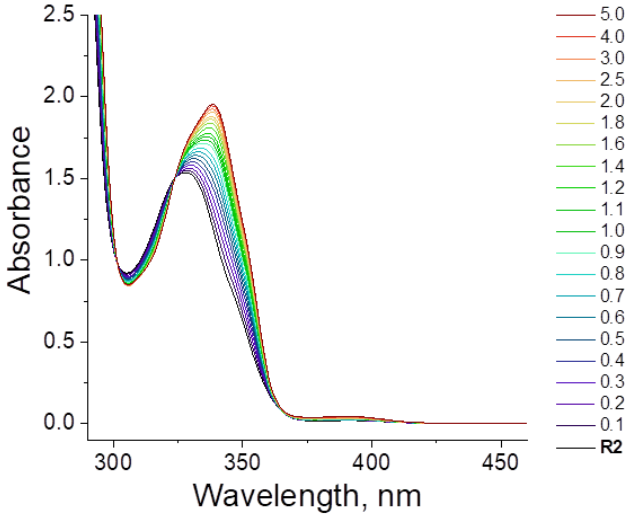

Figure S49. Stack of UV-vis spectra obtained during the titration of 10^{-4} M solution of receptor **R2** in DMSO/0.5% H_2O with 0.0075 M solution of $TBAH_2PO_4$ (dissolved in the solution of receptor **R2**).

Raw Data

Added volume of titrant solution [µL]	Equivalents of TBAPhCOO	Wavelength [nm]					
		338	339	340	341	342	350
		Absorbance					
0	0.0	1.222	1.163	1.103	1.043	0.990	0.623
4	0.1	1.281	1.227	1.171	1.113	1.060	0.671
8	0.2	1.341	1.291	1.238	1.183	1.130	0.718
12	0.3	1.400	1.355	1.306	1.252	1.199	0.765
16	0.4	1.459	1.419	1.373	1.321	1.268	0.812
20	0.5	1.508	1.472	1.428	1.378	1.326	0.851
24	0.6	1.557	1.525	1.484	1.436	1.384	0.891
28	0.7	1.602	1.573	1.534	1.488	1.436	0.926
32	0.8	1.643	1.617	1.581	1.536	1.484	0.960
37	0.9	1.691	1.669	1.636	1.591	1.540	0.999
41	1.0	1.720	1.699	1.668	1.625	1.574	1.023
45	1.1	1.746	1.728	1.698	1.656	1.605	1.044
49	1.2	1.770	1.753	1.726	1.684	1.633	1.064
57	1.4	1.809	1.796	1.770	1.731	1.680	1.097
65	1.6	1.841	1.830	1.806	1.767	1.716	1.122
74	1.8	1.864	1.855	1.833	1.795	1.745	1.143
82	2.0	1.881	1.874	1.853	1.815	1.766	1.158
103	2.5	1.908	1.903	1.884	1.847	1.798	1.182
125	3.0	1.926	1.922	1.904	1.869	1.820	1.198
169	4.0	1.945	1.943	1.926	1.891	1.843	1.216
214	5.0	1.955	1.954	1.938	1.905	1.856	1.228

Exemplary UV-vis titration curve (absorbance at 340 nm)

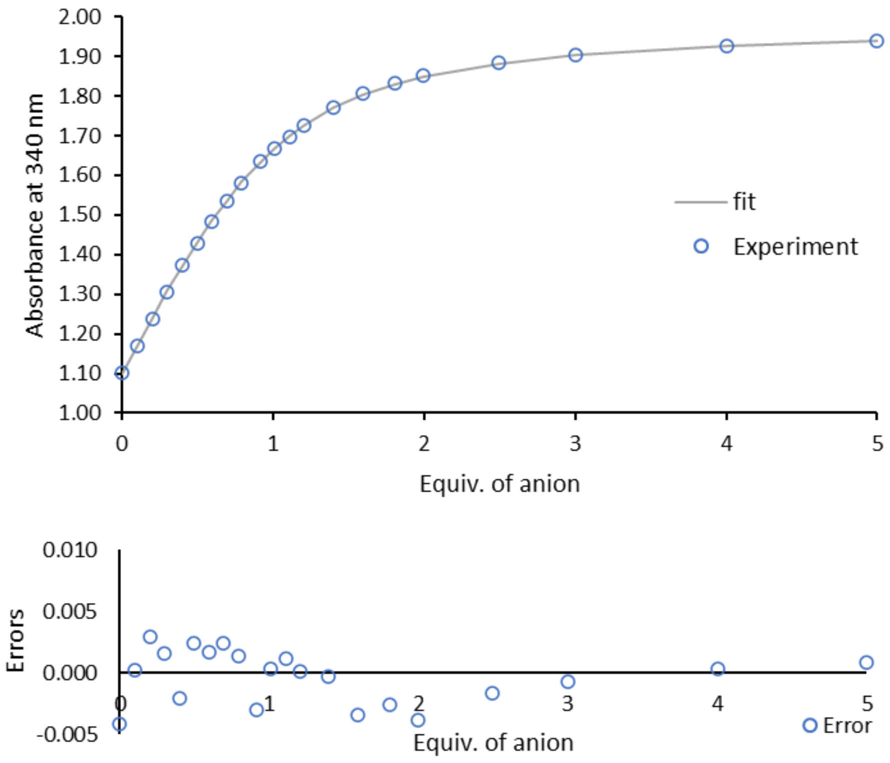

Figure S50. Fitting of 1:1 (receptor:anion) model to the results of the titration of 10^{-4} M solution of receptor **R2** in DMSO/0.5%H_2O with 0.0075 M solution of $TBAH_2PO_4$ (dissolved in the solution of receptor **R2**).

a) Binding constant K derived from simultaneous fitting of 1:1 model to the six selected wavelengths using HypSpec:

log K = 4.6986 ± 0.0005

b) Binding constant K derived from the experiment repeated according to the same methodology:

log K = 4.7325 ± 0.0005

c) Binding constant averaged from the two experiments:

log K = 4.716

3.14 UV-vis titration of **R2** with TBAPhCOO.

UV-vis titration of 10^{-4} M solution of **R2** in DMSO/0.5% H_2O with 0.0075 M solution of TBA^+PhCOO^- (dissolved in the solution of receptor **R2**).

Stack of UV-vis spectra from the titration of R2 with TBAPhCOO in DMSO/0.5%H_2O

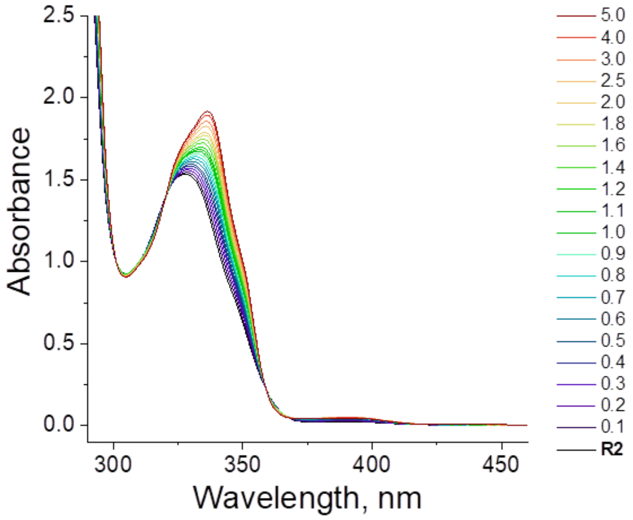

Figure S51. Stack of UV-vis spectra obtained during the titration of 10^{-4} M solution of receptor **R2** in DMSO/0.5% H_2O with 0.0075 M solution of TBAPhCOO (dissolved in the solution of receptor **R2**).

Raw Data

Added volume of titrant solution [µL]	Equivalents of TBAPhCOO	Wavelength [nm]					
		336	337	338	339	340	341
		Absorbance					
0	0.0	1.334	1.278	1.220	1.161	1.101	1.042
4	0.1	1.371	1.319	1.262	1.205	1.146	1.085
8	0.2	1.409	1.360	1.307	1.251	1.191	1.130
12	0.3	1.444	1.399	1.347	1.293	1.233	1.171
16	0.4	1.477	1.434	1.385	1.331	1.271	1.207
20	0.5	1.503	1.463	1.416	1.363	1.304	1.239
24	0.6	1.538	1.502	1.457	1.405	1.345	1.279
28	0.7	1.566	1.532	1.488	1.437	1.377	1.311
32	0.8	1.595	1.564	1.523	1.473	1.413	1.345
37	0.9	1.620	1.591	1.551	1.502	1.442	1.372
41	1.0	1.644	1.617	1.578	1.530	1.471	1.401
45	1.1	1.662	1.636	1.600	1.552	1.493	1.421
49	1.2	1.681	1.657	1.622	1.575	1.515	1.444
57	1.4	1.714	1.694	1.660	1.615	1.555	1.482
65	1.6	1.743	1.725	1.693	1.649	1.588	1.515
74	1.8	1.769	1.753	1.723	1.679	1.619	1.544
82	2.0	1.789	1.775	1.747	1.704	1.644	1.568
103	2.5	1.829	1.818	1.792	1.750	1.689	1.612
125	3.0	1.859	1.851	1.827	1.785	1.725	1.646
169	4.0	1.897	1.892	1.870	1.830	1.769	1.689
214	5.0	1.919	1.916	1.896	1.856	1.795	1.714
462	10.0	1.967	1.967	1.949	1.911	1.850	1.767

Exemplary UV-vis titration curve (absorbance at 338 nm)

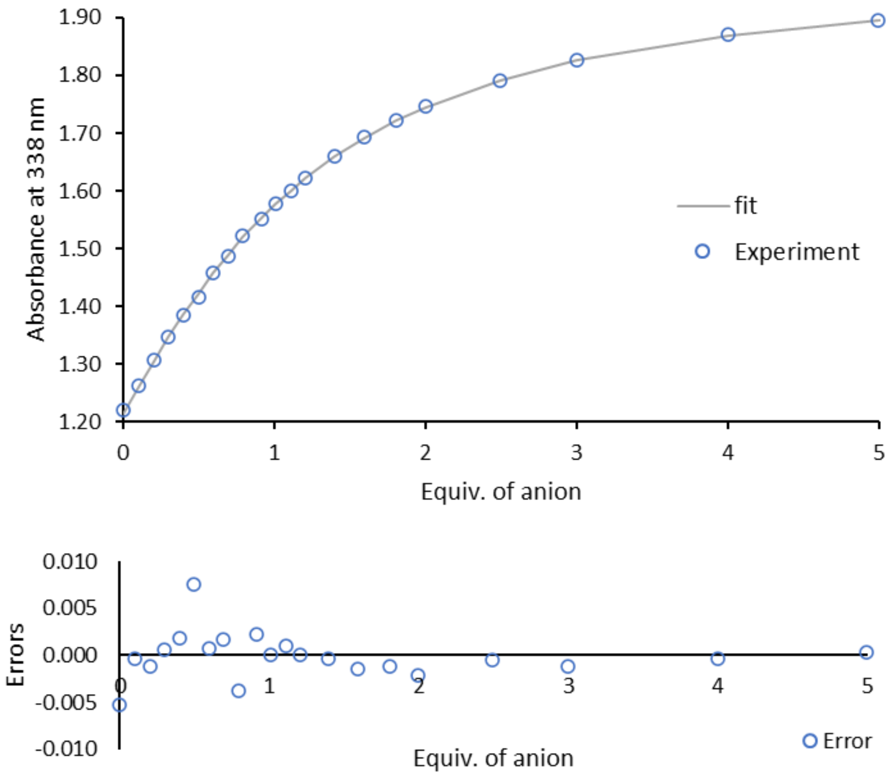

Figure S52. Fitting of 1:1 (receptor:anion) model to the results of the titration of 10^{-4} M solution of receptor **R2** in DMSO/0.5%H_2O with 0.0075 M solution of TBAPhCOO (dissolved in the solution of receptor **R2**).

a) Binding constant K derived from simultaneous fitting of 1:1 model to the six selected wavelengths using HypSpec:

log K = 4.1921 ± 0.0004

a) Binding constant K derived from the experiment repeated according to the same methodology:

log K = 4.2120 ± 0.0003

b) Binding constant averaged from the two experiments:

log K = 4.202

3.15 UV-vis titration of **R3** with $TBAH_2PO_4$.

UV-vis titration of 10^{-4} M solution of **R3** in DMSO/0.5% H_2O with 0.0075 M solution of $TBA^+H_2PO_4^-$ (dissolved in the solution of receptor **R3**).

Stack of UV-vis spectra from the titration of R3 with $TBAH_2PO_4$ in DMSO/0.5%H_2O

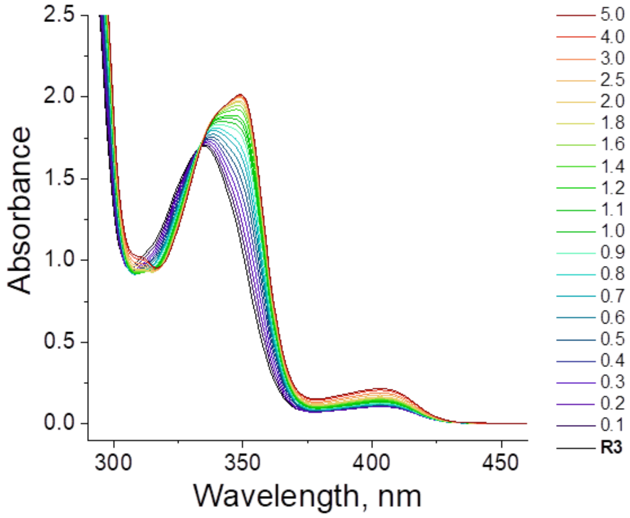

Figure S53. Stack of UV-vis spectra obtained during the titration of 10^{-4} M solution of receptor **R3** in DMSO/0.5% H_2O with 0.0075 M solution of $TBAH_2PO_4$ (dissolved in the solution of receptor **R3**).

Raw Data

Added volume of titrant solution [μL]	Equivalents of TBAPhCOO	Wavelength [nm]					
		338	339	340	341	342	350
		Absorbance					
0	0.0	1.662	1.634	1.599	1.558	1.515	1.043
4	0.1	1.681	1.657	1.628	1.592	1.553	1.124
8	0.2	1.702	1.683	1.657	1.625	1.591	1.203
12	0.3	1.722	1.708	1.687	1.661	1.632	1.295
16	0.4	1.741	1.731	1.715	1.694	1.670	1.382
20	0.5	1.757	1.752	1.740	1.724	1.705	1.463
24	0.6	1.774	1.773	1.766	1.754	1.739	1.537
28	0.7	1.796	1.798	1.794	1.786	1.776	1.611
32	0.8	1.807	1.812	1.812	1.808	1.801	1.670
37	0.9	1.821	1.830	1.834	1.834	1.832	1.742
41	1.0	1.831	1.842	1.849	1.852	1.852	1.786
45	1.1	1.839	1.853	1.861	1.867	1.869	1.824
49	1.2	1.844	1.859	1.870	1.877	1.881	1.853
57	1.4	1.853	1.871	1.884	1.894	1.902	1.901
65	1.6	1.859	1.878	1.893	1.905	1.914	1.931
74	1.8	1.862	1.883	1.900	1.913	1.924	1.954
82	2.0	1.863	1.884	1.902	1.916	1.928	1.966
103	2.5	1.862	1.886	1.905	1.920	1.933	1.986
125	3.0	1.861	1.884	1.905	1.921	1.935	1.995
169	4.0	1.857	1.881	1.902	1.920	1.935	2.005
214	5.0	1.855	1.880	1.901	1.919	1.935	2.010
462	10.0	1.853	1.879	1.901	1.920	1.936	2.020

Exemplary UV-vis titration curve (absorbance at 340 nm)

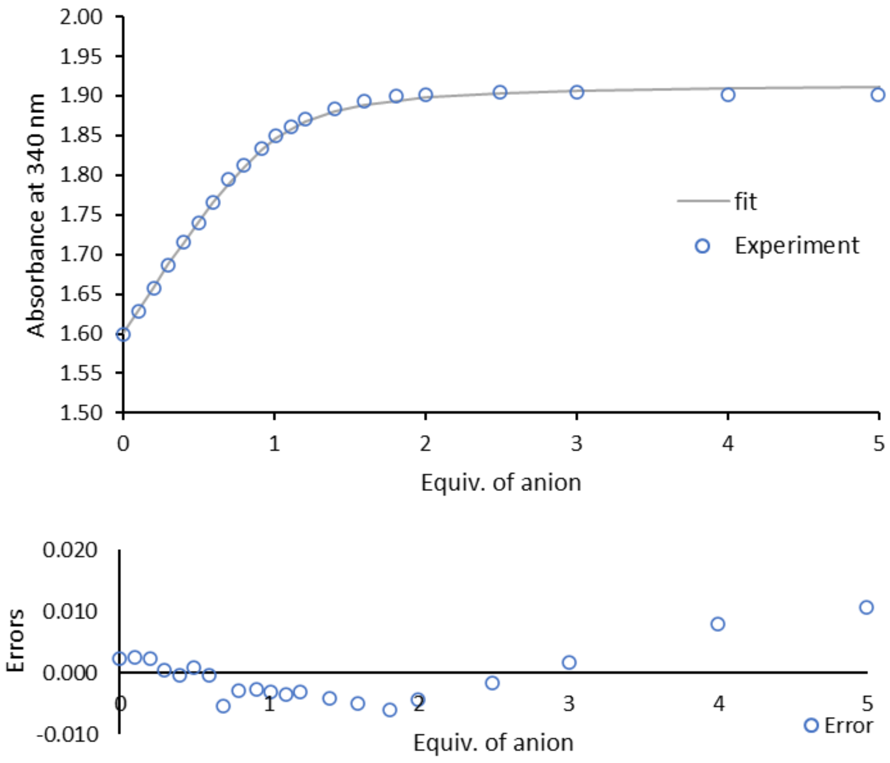

Figure S54. Fitting of 1:1 (receptor:anion) model to the results of the titration of 10^{-4} M solution of receptor **R3** in DMSO/0.5%H_2O with 0.0075 M solution of $TBAH_2PO_4$ (dissolved in the solution of receptor **R3**).

a) Binding constant K derived from simultaneous fitting of 1:1 model to the six selected wavelengths using HypSpec:

log K = 5.1614 ± 0.0024

b) Binding constant K derived from the experiment repeated according to the same methodology:

log K = 5.2184 ± 0.0041

c) Binding constant averaged from the two experiments:

log K = 5.190

3.16 UV-vis titration of **R3** with TBAPhCOO.

UV-vis titration of 10^{-4} M solution of **R3** in DMSO/0.5% H_2O with 0.0075 M solution of TBA^+PhCOO^- (dissolved in the solution of receptor **R3**).

Stack of UV-vis spectra from the titration of R3 with TBAPhCOO in DMSO/0.5%H_2O

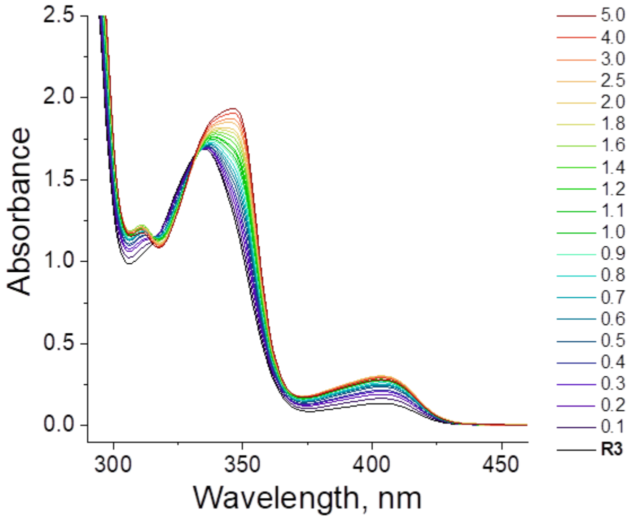

Figure S55. Stack of UV-vis spectra obtained during the titration of 10^{-4} M solution of receptor **R3** in DMSO/0.5% H_2O with 0.0075 M solution of TBAPhCOO (dissolved in the solution of receptor **R3**).

Raw Data

Added volume of titrant solution [μL]	Equivalents of TBAPhCOO	Wavelength [nm]					
		336	337	338	339	340	341
		Absorbance					
0	0.0	1.688	1.676	1.654	1.627	1.593	1.553
4	0.1	1.686	1.676	1.658	1.634	1.603	1.566
8	0.2	1.694	1.687	1.671	1.651	1.624	1.591
12	0.3	1.695	1.691	1.679	1.661	1.637	1.608
16	0.4	1.703	1.700	1.690	1.674	1.653	1.626
20	0.5	1.705	1.705	1.698	1.686	1.668	1.645
24	0.6	1.713	1.716	1.711	1.701	1.686	1.666
28	0.7	1.716	1.720	1.717	1.710	1.697	1.680
32	0.8	1.723	1.729	1.729	1.724	1.713	1.698
37	0.9	1.732	1.740	1.743	1.739	1.732	1.720
41	1.0	1.734	1.744	1.748	1.746	1.740	1.730
45	1.1	1.744	1.755	1.760	1.761	1.756	1.748
49	1.2	1.743	1.756	1.763	1.764	1.761	1.754
57	1.4	1.752	1.766	1.776	1.779	1.779	1.775
65	1.6	1.760	1.776	1.787	1.792	1.793	1.792
74	1.8	1.765	1.783	1.796	1.803	1.806	1.807
82	2.0	1.771	1.790	1.804	1.813	1.817	1.819
103	2.5	1.783	1.804	1.820	1.831	1.838	1.843
125	3.0	1.791	1.813	1.831	1.843	1.852	1.859
169	4.0	1.806	1.830	1.849	1.863	1.874	1.883
214	5.0	1.820	1.845	1.865	1.880	1.893	1.903
462	10.0	1.859	1.887	1.908	1.924	1.938	1.950

Exemplary UV-vis titration curve (absorbance at 338 nm)

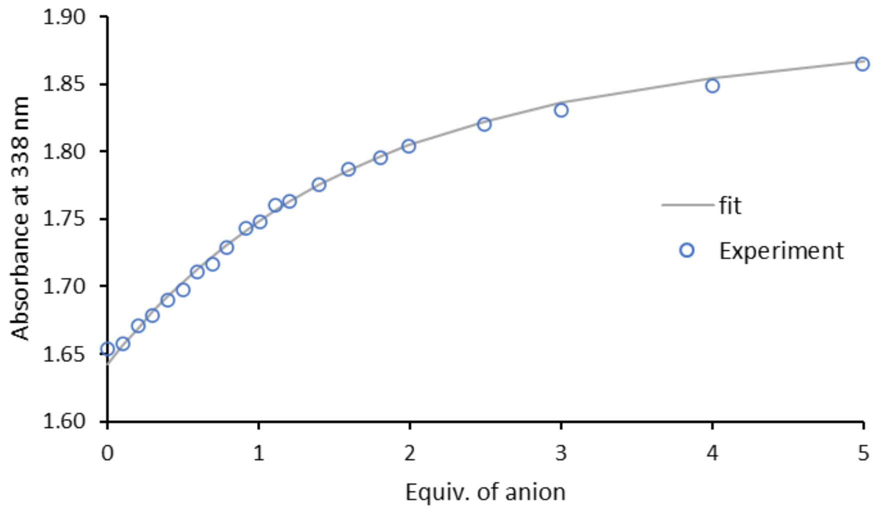

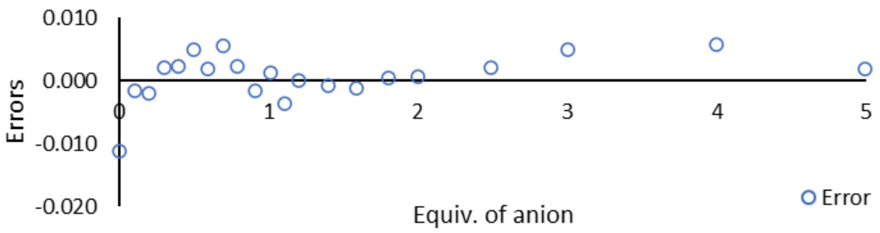

Figure S56. Fitting of 1:1 (receptor:anion) model to the results of the titration of 10^{-4} M solution of receptor **R3** in DMSO/0.5%H_2O with 0.0075 M solution of TBAPhCOO (dissolved in the solution of receptor **R3**).

a) Binding constant K derived from simultaneous fitting of 1:1 model to the six selected wavelengths using HypSpec:

log K = 3.9909 ± 0.0026

b) Binding constant K derived from the experiment repeated according to the same methodology:

log K = 4.0325 ± 0.0030

c) Binding constant averaged from the two experiments:

log K = 4.012

4 Fluorescence studies of R1

To a solution of a host (2.5 ml, 10^{-5} M) in a screw-cap fluorescence cuvette made of SUPRASIL quartz (optical path length: 10 mm), appropriate aliquots of titrant (dissolved in the solution of the host to avoid dilution) were added using a 25 µl gas-tight microsyringe. Fluorescence spectra were obtained on Hitachi F-7000 spectrofluorometer. Excitation wavelength: 324 nm, scan speed: 1200 nm/min, temperature: 25°C.

Stack of fluorescence spectra from the titration of R1 with various TBA salts in DMSO/0.5% H_2O

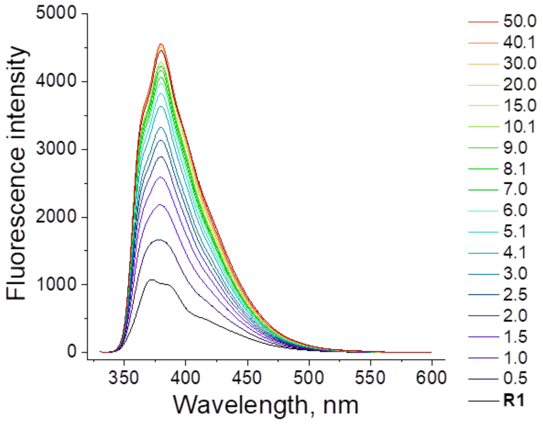

Anion: $H_2PO_4^-$, Excitation: 324 nm.

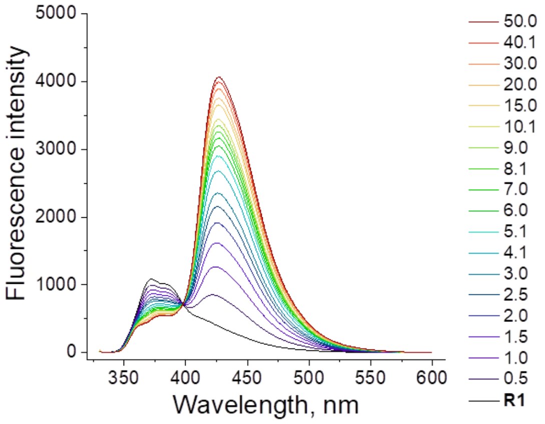

Anion: CH_3COO^-, Excitation: 324 nm.

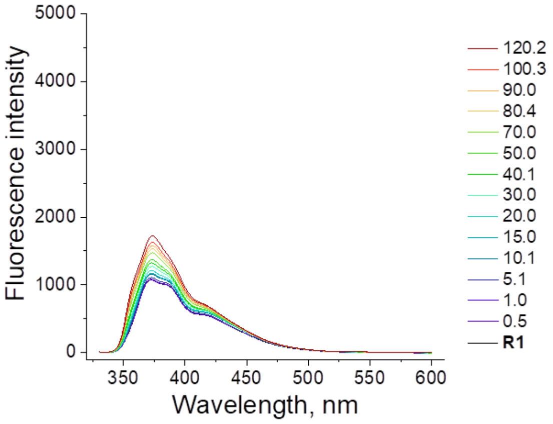

Anion: Cl^-, Excitation: 324 nm.

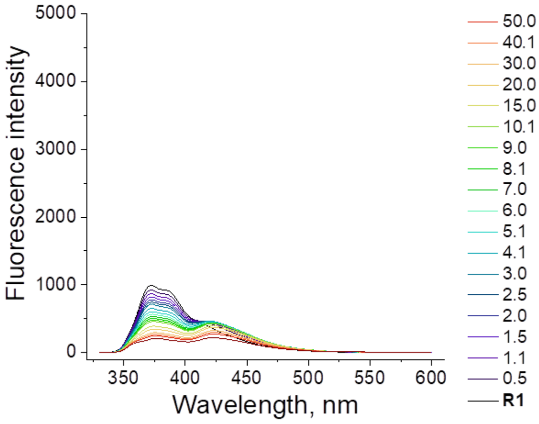

Anion: $PhCOO^-$, Excitation: 324 nm.

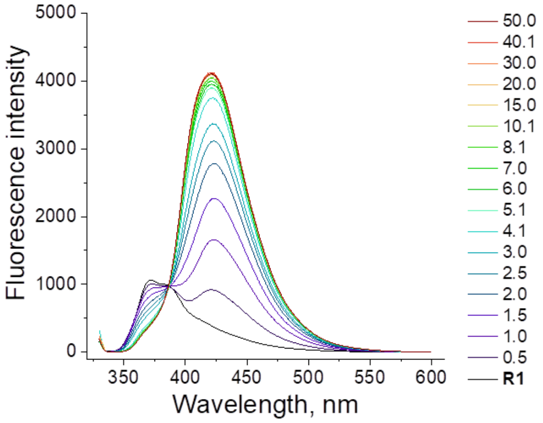

Anion: SO_4^{2-}, Excitation: 324 nm.

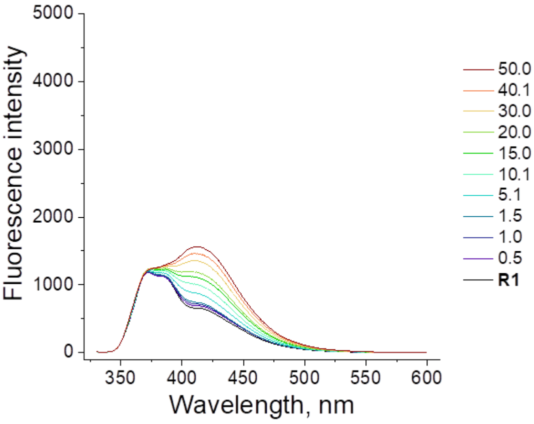

Anion: HSO_4^-, Excitation: 324 nm.

Figure S57. Stack of fluorescence spectra obtained during the titration of 10^{-5} M solution of receptor **R1** in DMSO/0.5% H_2O with 0.00375 M solutions of various TBA salts (dissolved in the solution of receptor **R1**).

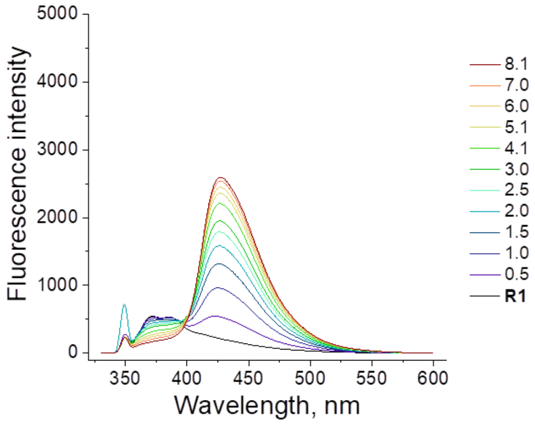

Anion: OH⁻, Excitation: 324 nm.

Figure S58. Fluorescence spectra obtained during the titration of 10^{-5} M solution of receptor **R1** in DMSO/0.5% H_2O with 0.00375 M solutions of TBAOH salt (dissolved in the solution of receptor **R1**).

5 Determination of $H_2PO_4^-$ in water using R1

A stock solution of sodium dihydrogen phosphate ($NaH_2PO_4 \times 2H_2O$, 5 ml, 2.5 mM) was prepared in fresh Milli-Q water. This stock solution was then used to prepare 10 ml each of four diluted solutions, with concentrations of 0.05, 0.1, 0.25, and 0.5 mM (C_1-C_4). A mixture of DMSO (2.7 ml), aqueous $H_2PO_4^-$ solutions of varying concentrations (0.3 ml each, or pure Milli-Q water for the C_{H2PO4} = 0 mM), and 30 µL of **R1** in DMSO, was placed in a quartz cuvette to achieve a final concentration of **R1** = 10^{-5} M. The fluorescence intensity of each mixture was measured at 380 nm upon excitation at EX WL = 324 nm, and the obtained values were used to construct the calibration curve shown below:

Calibration curve for the determination of H_2PO_4 in water using R1 in DMSO

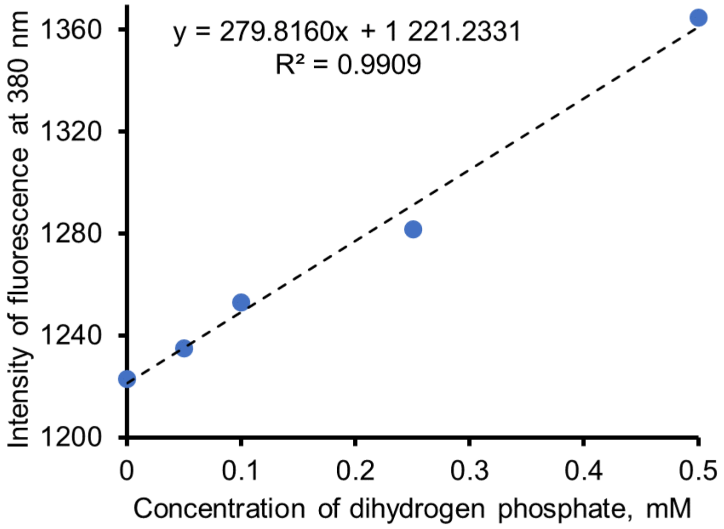

Figure S59. Calibration curve for dihydrogen phosphate solutions based on fluorescence emission at 380 nm for 10^{-5} M of **R1**.

A sample solution of dihydrogen phosphate in Milli-Q water, having a known concentration of 0.075 mM, was analyzed using the DMSO solution of **R1** and the calibration curve shown above.

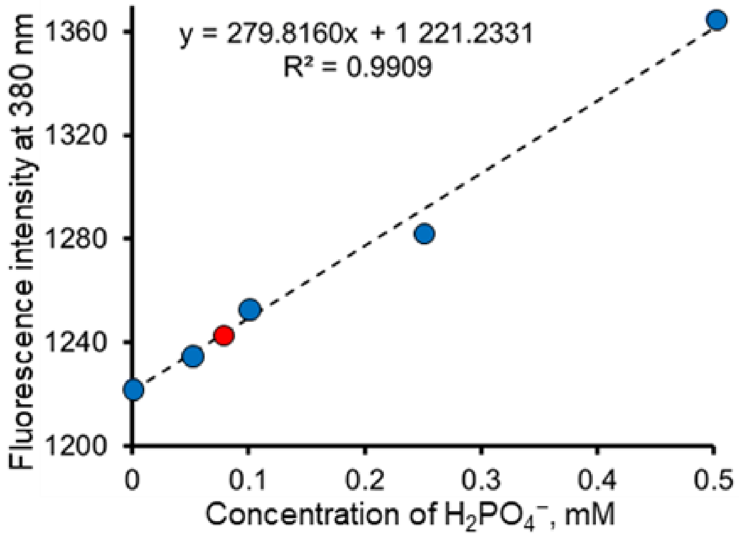

Figure S60. Calibration curve for dihydrogen phosphate solutions based on fluorescence emission at 380 nm for 10^{-5} M of **R1**.

The absolute error of the method was calculated using the following equation:

$$\%_{error} = \frac{C_c - C_r}{C_r} \cdot 100\%,$$

where C_c is the concentration of $H_2PO_4^-$ calculated from the curve, and C_r is the real concentration of the sample.

$$\%_{error} = 3.7\%.$$

Raw data

Sample	Fluorescence Intensity at 380 nm	$H_2PO_4^-$ Concentration (mM)
C_0	1223	0
C_1	1235	0.05
C_2	1253	0.10
C_3	1282	0.25
C_4	1365	0.50
Sample	1243	0.075 (*actual*)<br>0.078 (*calculated*)

The detection limit for $H_2PO_4^-$ was determined using the equation:

$$LOD = \frac{3SD}{a},$$

where SD is the standard deviation from three blank measurements, and a is the slope of the fluorescence intensity versus the sample concentration. The obtained detection limit was:

LOD = 0.02 mM.

Standard deviation calculation:

$$SD = \sqrt{\frac{\sum(x_i - \bar{x})^2}{N-1}},$$

where:
$\bar{x}$ is the sample mean,
x_i is the individual sample value,
N is number of samples

5.1 Determination of solubility of receptors **R1**, **R2**, **R3** and **R5**.

All the receptors (approximately 2 mg) were weighted separately on a Mettler Toledo Excellence XA105DU analytical balance (readability 0.01 mg) in vials. Solvent was then added to each vial until a homogeneous solution was achieved in room temperature. The weight of the solvent used was recorded, and the solubility was calculated as the amount dissolved per 100 g of solvent.

Solvent	Solubility for 100 g of solvent [mg]			
	**R1**	**R2**	**R3**	**R5**
Acetonitrile	13.8	4.9	5.6	3.2
Ethyl acetate	13.6	10.8	33.4	4.4
